# Comparative proteomic profiling reveals mechanisms for early spinal cord vulnerability in CLN1 disease

**DOI:** 10.1038/s41598-020-72075-7

**Published:** 2020-09-16

**Authors:** Hemanth R. Nelvagal, Maica Llavero Hurtado, Samantha L. Eaton, Rachel A. Kline, Douglas J. Lamont, Mark S. Sands, Thomas M. Wishart, Jonathan D. Cooper

**Affiliations:** 1 Division of Genetics and Genomic Medicine, Department of Pediatrics, Washington University in St Louis, School of Medicine, 660 S Euclid Ave, St Louis, MO 63110 USA; 2 Department of Genetics, Washington University in St Louis, School of Medicine, 660 S Euclid Ave, St Louis, MO 63110 USA; 3 Department of Neurology, Washington University in St Louis, School of Medicine, 660 S Euclid Ave, St Louis, MO 63110 USA; 4 Department of Medicine, Washington University in St Louis, School of Medicine, 660 S Euclid Ave, St Louis, MO 63110 USA; 5 Department of Basic and Clinical Neuroscience, Institute of Psychiatry, Psychology and Neuroscience, King’s College London, London, UK; 6 The Roslin Institute and Royal (Dick) School of Veterinary Studies, University of Edinburgh, Easter Bush, Midlothian, UK; 7 FingerPrints Proteomics Facility, College of Life Sciences, University of Dundee, Dundee, UK

**Keywords:** Lipid-storage diseases, Neurodegeneration

## Abstract

CLN1 disease is a fatal inherited neurodegenerative lysosomal storage disease of early childhood, caused by mutations in the *CLN1* gene, which encodes the enzyme Palmitoyl protein thioesterase-1 (PPT-1). We recently found significant spinal pathology in Ppt1-deficient (*Ppt1*^*−/−*^) mice and human CLN1 disease that contributes to clinical outcome and precedes the onset of brain pathology. Here, we quantified this spinal pathology at 3 and 7 months of age revealing significant and progressive glial activation and vulnerability of spinal interneurons. Tandem mass tagged proteomic analysis of the spinal cord of *Ppt1*^*−/−*^and control mice at these timepoints revealed a significant neuroimmune response and changes in mitochondrial function, cell-signalling pathways and developmental processes. Comparing proteomic changes in the spinal cord and cortex at 3 months revealed many similarly affected processes, except the inflammatory response. These proteomic and pathological data from this largely unexplored region of the CNS may help explain the limited success of previous brain-directed therapies. These data also fundamentally change our understanding of the progressive, site-specific nature of CLN1 disease pathogenesis, and highlight the importance of the neuroimmune response. This should greatly impact our approach to the timing and targeting of future therapeutic trials for this and similar disorders.

## Introduction

The neuronal ceroid lipofuscinoses (NCLs) are a group of fatal inherited neurodegenerative lysosomal storage disorders affecting children and young adults^1,2^. CLN1 disease or infantile NCL is a rapidly progressing form caused by mutations in the *CLN1* gene, which encodes a lysosomal de-palmitoylating enzyme, Palmitoyl Protein Thioesterase-1 (PPT-1)^3,4^. Despite the recent development an FDA-approved disease-limiting therapy for CLN2 disease, there no curative therapy for CLN1 disease^5^.

This lack of therapeutic efficacy may be due to other disease loci not targeted by brain-directed therapies^6–8^. We recently demonstrated the existence of significant pathology in the spinal cords of human CLN1 patients at autopsy and in Ppt1-deficient (*Ppt1*^*−/−*^) mice^9^. The onset of spinal pathology in *Ppt1*^*−/−*^ mice preceded similar changes in the brain, and therapeutically targeting the spinal cord of *Ppt1*^*−/−*^ mice either alone or in combination with the brain, significantly ameliorated pathology and improved lifespan^9^. However, the exact nature and extent of spinal pathology, particularly before the brain is affected is yet to be defined, and the mechanisms underlying such regional vulnerability are yet to be elucidated.

In this study, we characterized pathological changes in the spinal cords of *Ppt1*^*−/−*^ mice at 3 months, the timepoint when we first saw significant neuron loss, and at disease end-stage at 7 months^9^. This revealed significant changes in spinal cord volume, glial activation and interneuron survival at 3 months of age. We then used comparative proteomic profiling strategies to help elucidate the underlying mechanisms of this regional pathology. Our findings indicate the alteration of distinct cell-signalling pathways, developmental processes, mitochondrial dysfunction and most notably, a significant neuroinflammatory response in the spinal cords of *Ppt1*^*−/−*^ mice at 3 months of age that is not observed in their cortex at the same age. Together, these data highlight the significant and early extent of disease in the CLN1 disease spinal cord, identifying vulnerable spinal cell populations and region-specific disease mechanisms that may underlie spinal cord vulnerability in this disease.

## Results

### Early and progressive pathological changes in *Ppt1*^*−/−*^ spinal cord

**Figure 1:**
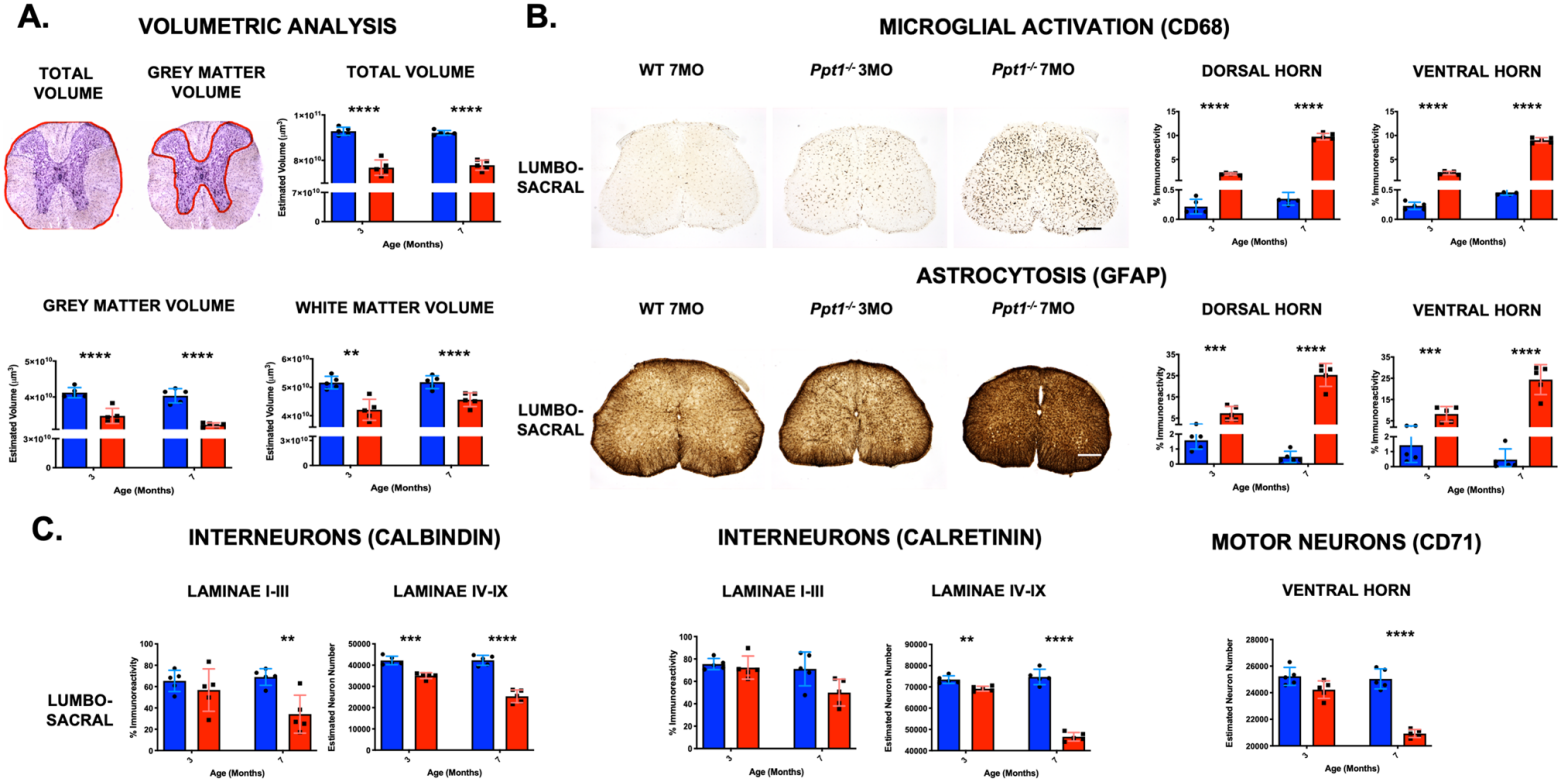
Early and progressive spinal cord pathology in *Ppt1*^*−/−*^ mice. Stereological analysis of regional volume (**A**) in the spinal cord reveals significant reduction in total, grey and white matter in whole *Ppt1*^*−/−*^ cords at 3 and 7 months of age compared to wildtype (WT) controls. Thresholding imaging analysis (**B**) of sections stained for microglia (CD68) and astrocytes (GFAP) show a significant increase in both markers in the dorsal and ventral horns of the lumbo-sacral cord at early (3MO) and late (7MO) disease states in *Ppt1*^*−/−*^ cords compared to WT. Counts of neuron number in the lumbo-sacral cord (**C**) revealed a significant loss of *Ppt1*^*−/−*^ mouse spinal interneurons stained with calbindin and calretinin in laminae IV-IX as early as 3 months of age, compared to WT. However, spinal motor neurons in the ventral horns (CD-71) were only significantly lost at 7 months of age in *Ppt1*^*−/−*^ mouse spinal cords. Scale bars 200 µm. p values—**p ≤ 0.01, ***p ≤ 0.001, ****p ≤ 0.0001; multiple two-tailed, unpaired, parametric t test with Bonferroni–Dunn correction. Values shown are mean ± SEM. (n = 5 mice/group).

Stereological estimates of Nissl-stained sections of 3 and 7 month old wildtype and *Ppt1*^*−/−*^ mouse spinal cords^10,11^ revealed significantly lower total volume, grey matter and white matter volumes at 3 months of age in *Ppt1*^*−/−*^ mice, which worsened by 7 months of age (Fig. 1A). In marked contrast, the brains of these mice show little white matter pathology any stage of disease progression, and no significant regional atrophy until 5 months of age^10^. Analyzing the cervical and lumbo-sacral regions of the *Ppt1*^−/−^ cord separately revealed similar changes at both levels, with only cervical white matter in *Ppt1*^*−/−*^ mice at 3 months of age not showing significantly reduced volume. This is indicative of little rostro-caudal difference in these volumetric changes in the *Ppt1*^*−/−*^ spinal cord (Supplementary Figure S1).

We have previously qualitatively described dramatic increases in astrocytosis and microglial activation in the grey matter of cervical and lumbar spinal cords of *Ppt1*^*−/−*^ mice^9^. Quantifying similarly stained sections for GFAP (astrocytes) and CD68 (microglia), showed a significant and progressive increase in both markers from 3 months of age that equally affected the dorsal and ventral horns, and occurred both in the cervical and lumbo-sacral regions (Fig. 1B, Supplementary Figure S1). The similar effects within dorsal and ventral horns suggest that these glial changes are not confined to either sensory or motor pathways, unlike the regional specificity observed in the brain and cerebellum^12,13^. We previously showed significant loss of Nissl-stained neurons as early as 3 months of age in the cervical and lumbar spinal cords of *Ppt1*^*−/−*^ mice^9^ that occurs before any neuron loss in the brain^12^. To further characterize the nature of neuron loss in the spinal cord, we analyzed sections stained for interneuron markers (calbindin, calretinin) and motor neurons (CD-71), which represent major neuronal populations in the spinal grey matter^14,15^. This unbiased stereological analysis revealed a significant loss of interneurons in both the cervical and lumbo-sacral cords of *Ppt1*^*−/−*^ mice at 3 months of age, whereas motor neurons were only significantly decreased at 7 months of age (Fig. 1C; Supplementary Figure S1). Therefore, the Nissl stained neuron loss seen in these mice^9^ is likely due to interneuron loss rather than loss of motor neurons. Importantly, these interneuron populations are potentially more vulnerable to CLN1 disease and show a progressive decline in number between 3 and 7 months, as they do in the brain^12^.

Together, these data establish the spinal cord as a particularly vulnerable region of the CNS in CLN1 disease, with significant pathological changes evident earlier than the brain regions we have previously characterized^11–13^. The loss of interneurons before motor neurons is indicative of these being vulnerable neuronal populations, as seen in the forebrain^12^. However, the lack of rostro-caudal differences or differences between the dorsal and ventral horns, shows there to be a more widespread and less regionally selective pathology in the spinal cord.

### Quantitative proteomic profiling of whole tissue lysates spinal cord and cortex at early and late stages (3 and 7 months)

**Figure 2:**
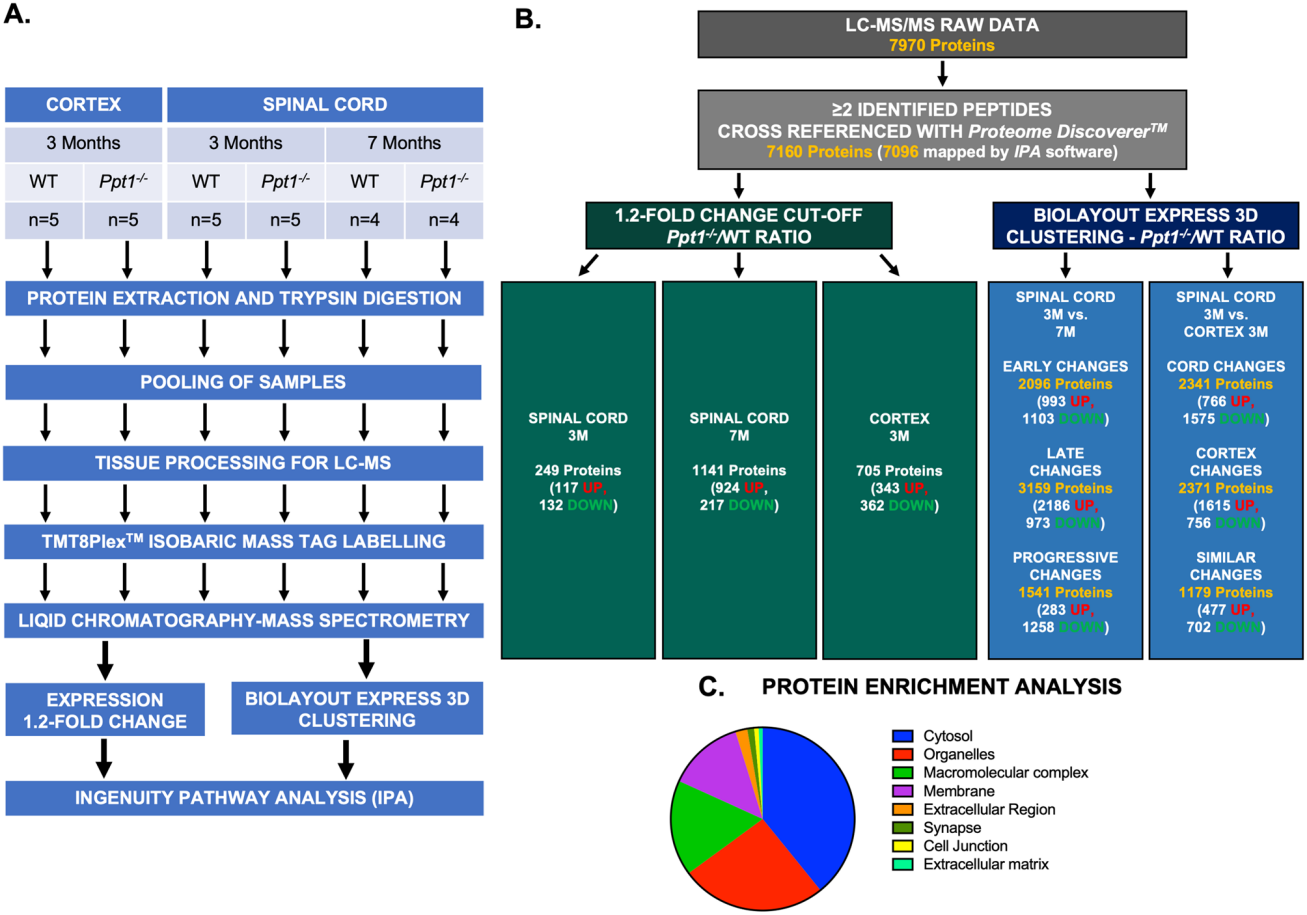
Experimental design workflow and sample validation. Schematic (**A**) diagram representing the various stages of experimental workflow for LC–MS/MS and in silico analysis of proteomic data set. (**B**) Schematic depicting the filtration of raw LC–MS data by first identifying those with ≥ 2 peptides, cross-references with *Proteome Discoverer*. These were then filtered using a 1.2-fold cut off for *Ppt1*^*−/−*^/WT ratios for each individual comparison or without a cut-off by using *BioLayout Express 3D* clustering. The numbers of resulting proteins used for each comparison are referenced below. Filtered proteins were then input for IPA analysis. Pie chart diagram (**C**) of the main biological function/subcellular compartments identified by *Panther* enrichment analysis showing a lack of any particular enrichment of proteins.

To further characterize the changes occurring in the spinal cord in CLN1  disease, we processed whole wildtype and *Ppt1*^*−/−*^ cortical and spinal cord extracts using Tandem mass tagging (TMT) based quantitative proteomics^16,17^. We analyzed 6 groups in detail- 3 month cortices and spinal cords as well as 7 month spinal cords from WT and *Ppt1*^−/−^ mice respectively (Fig. 2A, https://doi.org/10.7488/ds/2750 (2020)). The 7,970 protein raw data outputs from LC–MS were filtered to include proteins with ≥ 2 peptides identified, considered more reliable observations^18^. These proteins were then cross-referenced with mouse (*Mus musculus*) protein sequences from *UniProtKB/Swiss-Prot* using the MASCOT search engine (Matrix Science, Version 2.2) through *Proteome Discoverer* (Version 1.4, ThermoFisher) for a total of 7,160 identified proteins (https://doi.org/10.7488/ds/2750 (2020)) (Fig. 2B). We then used the *PANTHER* gene ontology database^19^ (Fig. 2C) and the Database for Annotation, Visualization and Integrated Discovery (*DAVID*)^20,21^ to confirm that the list of proteins identified was representative of whole tissue lysates and not enriched in any particular sub-cellular compartment (Supplementary David Ontology File). LC–MS data for markers shown to be altered from spinal cord immunohistochemistry (Fig. 1) were identified showing an increase in CD68 ratios. However, GFAP was not greatly increased in the 3-month-old spinal cord ratios, presumably due to its high expression in the white matter of the cord, even in WT tissue, but this increased at 7 months, and was also increased in the cortex at 3 months. There was little change in the expression of interneuron markers calbindin and calretinin, possibly due to the relatively low abundance of cells expressing these markers (Supplementary Table S1). We also validated these data using quantitative fluorescent western blotting (QFWB) of representative proteins from samples used for LC–MS (Supplementary Figure S2). Representative proteins that showed increased, decreased or relatively unaltered *Ppt1*^*−/−/*^WT abundance ratios, together with markers relevant to CLN1 disease pathology were chosen to appropriately validate the LC–MS data, including markers for synaptic proteins^22^, astrocytes, interneurons^11,12^ and for oligodendrocytes (MBP). Overall our QFWB analysis showed similar trends to the predicted LC–MS data (Supplementary Table). SNAP25 which was significantly downregulated in the spinal cord as compared to the cortex, and synaptophysin showed little change across all groups. Little overall change in protein abundance was also observed in QFWB data for calbindin and cytochrome oxidase subunit IV (COX IV), consistent with LC–MS data (Supplementary Figure S2). These synaptic markers, calbindin and COX IV all showed an overall increased abundance in the cortex as compared to the spinal cord, as is expected given the higher density of cells expressing these markers in the cortex^15,23^. We also probed astrocyte markers—GFAP was significantly up regulated in the cortex at 3 months and spinal cord at 7 months, with only a trend to increased values at 3 months in the spinal cord, while glutamine synthetase did not show any significant change across groups, again consistent with the LC–MS data (Supplementary Figure S2). Lastly, Myelin basic protein (MBP) was more abundant in the spinal cord as compared to the cortex, which contains less white matter, but was decreased in 3 and 7 month *Ppt1*^*−/−*^ cords (Supplementary Figure S2). Functional proteomic analysis was carried out by filtering for proteins with increased stringency- either including those proteins with 1.2 fold (20%) expression change in *Ppt1*^*−/−*^ tissues, compared to wildtype^18,24^ or by *BioLayout Express 3D* to cluster proteins that show similar changes in expression^16–18,25–28^ (Fig. 2B). These data were then analyzed using the ingenuity pathway analysis (*IPA*) (Ingenuity systems) software to reveal the cellular pathways that may be altered in these different regions^16,18,26–28^.

#### Significant neuroimmune and inflammatory responses in *Ppt1*^*−/−*^ spinal cords at 3 months of age

**Figure 3:**
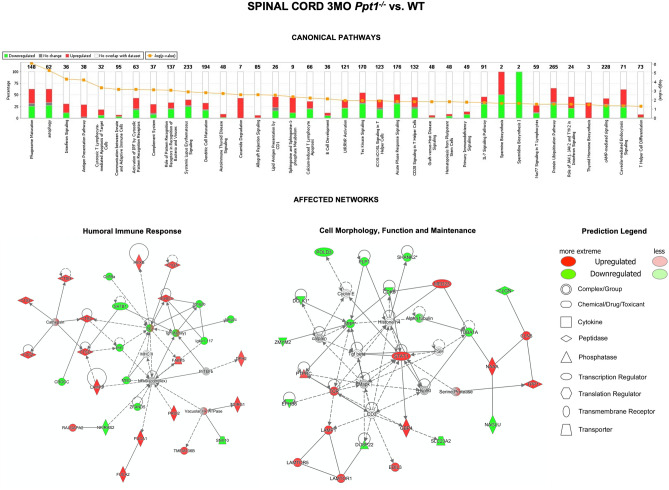
Early proteomic changes in the spinal cord of *Ppt1*^*−/−*^ mice. Analysis of the differentially expressed proteins in the spinal cord of *Ppt1*^*−/−*^ mice at 3 months of age. Proteins that were differentially expressed by 1.2 fold (20%) were linked using *Ingenuity Pathway Analysis* (*IPA*). The top affected canonical pathways (**A**) and two of the most significantly affected networks (**B**)—humoral immune response (*IPA* Score = 52, 28 focus molecules) and cell morphology, function and maintenance (*IPA* Score = 42, 24 focus molecules) are shown here. (See Supplementary Tables S2, S3 and Supplementary Excel File for further details.)

The expression ratios (*Ppt1*^*−/−*^/WT) from spinal cord comparisons at 3 months identified a total of 7,160 proteins and were evaluated using *IPA* software, which mapped the majority of the proteins to the Ingenuity Knowledge database (7,096). We applied 1.2-fold (20%) expression change (*Ppt1*^*−/−*^/WT) as a cut-off, which yielded 249 (117 up- and 132 downregulated) (Fig. 2B) differentially expressed proteins. *IPA* analysis of affected cellular (canonical) pathways showed significant changes in the inflammatory pathways in the 3 month *Ppt1*^*−/−*^ spinal cord. The top canonical pathways affected included phagosome maturation (p = 7.98E−07) and autophagy (p = 5.37E−06) (Fig. 3, Supplementary Table S2), which are anticipated given the intrinsic link between lysosomal function and autophagy^29–31^ and the significant microglial activation observed by immunohistochemistry (Fig. 1). Furthermore, interferon signalling (p = 4.48E−05), antigen presentation (p = 5.86E−05) and T-lymphocyte mediated apoptosis (p = 4.08E−04) were also affected indicative of a pronounced humoral immune system involvement at this earlier time point (Fig. 3).

**Figure 4:**
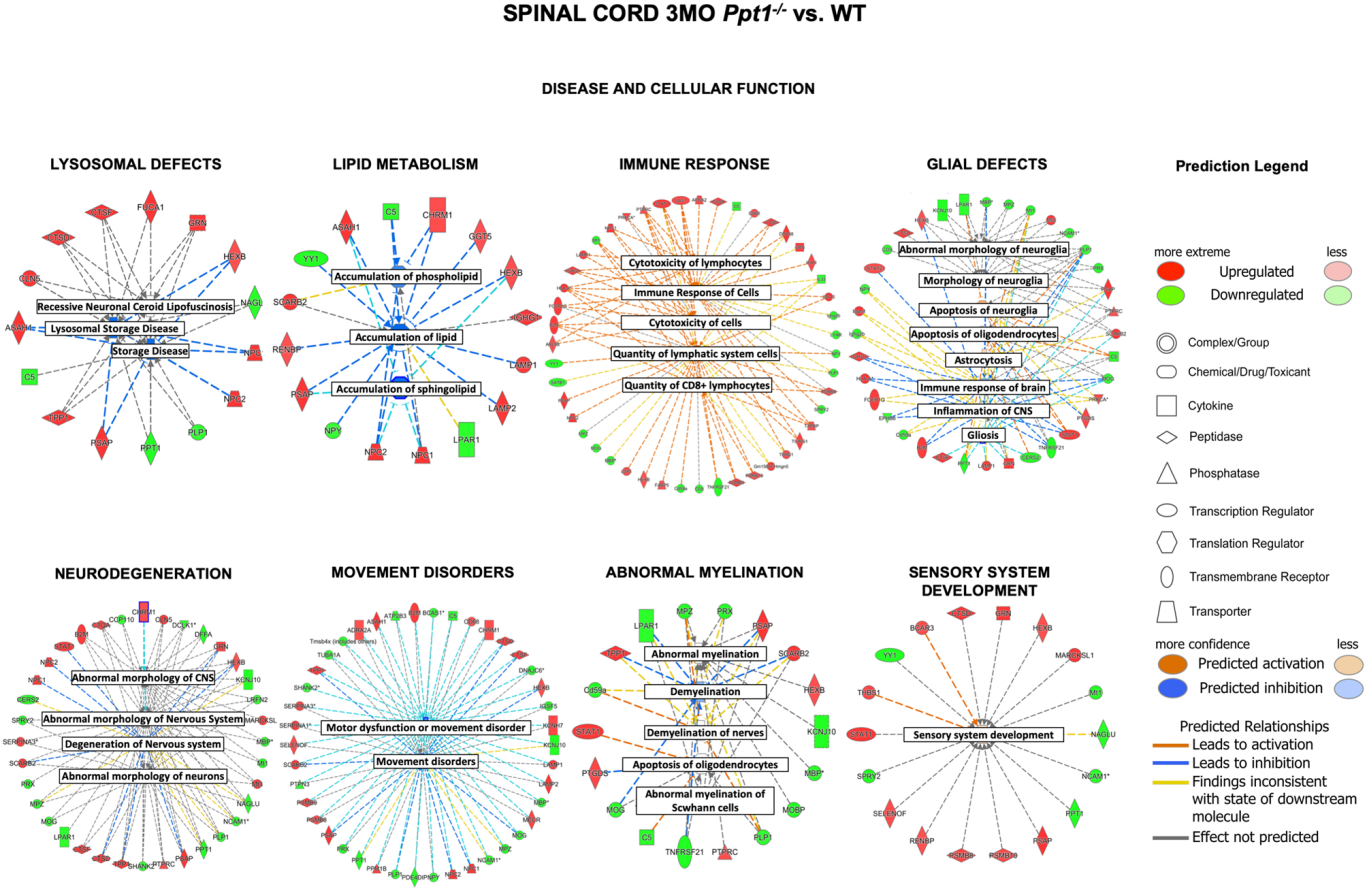
Early disease and/or cellular function changes in the spinal cord of *Ppt1*^*−/−*^ mice. Analysis of the differentially expressed proteins by 1.2 fold (20%) in the spinal cord of *Ppt1*^*−/−*^ mice at 3 months of age, linked using *Ingenuity Pathway Analysis* (*IPA*). Various processes affected included—lysosomal protein defects (p = 8.98E−12, z = − 2.193), accumulation of lipid (p = 1.38E4, z = − 3.229), immune response of cells and quantity of CD8 positive cells (p = 4.3E5, z = 3.252), glial morphology (p = 1.21E−09, z = − 0.68), neurodegenerative changes (p = 8.03E5, z = 0.813), movement disorders (p = 5.93E6, z = − 1.664), abnormal myelination (p = 8.71E−10, z = 0.898) and sensory system development (p = 7.39E4, z = 0.83). (Further details available including larger versions of images available in Supplementary Information, Supplementary Table S4 and Supplementary Excel File).

*IPA* software was utilized to determine which cellular networks are most affected by interrogating curated list of known networks of protein interactions. This analysis further highlighted the significant inflammatory response, as the most enriched networks involved—Humoral Immune Response, Inflammatory Response and Nutritional Disease (Fig. 3, Supplementary Table S2, *IPA*-Score = 52, 28 focus molecules). The next most enriched networks were Cell Morphology, Cellular Assembly and Organization (*IPA*-Score = 42, 24 focus molecules) (Fig. 3, Supplementary Table S3) and Cell-To-Cell Signalling and Interaction, Nervous System Development, Cell Morphology (*IPA*-Score = 35, 21 focus molecules). The top predicted upstream regulators also included a predicted activation of interferon gamma (p = 8.37E−12, activation z-score (z) = 4.11) and Interferon alpha/beta receptor (IfNAR, p = 6.51E−14, z = 3.831), which are linked with a humoral immune response. There was also a predicted activation of STAT1 (p = 2.25E−06, z = 3.795), interferon regulatory factor 7 (IRF7, p = 8.85E−10, z = 2.837) and inhibition of TRIM 24 (p = 3.33E−13, z = − 3.819). These predicted regulatory alterations emphasize the findings of affected canonical pathways and highlight the extent of inflammatory changes in the *Ppt1*^*−/−*^ spinal cord at 3 months of age. Using the *IPA* analysis to further dissect which pathological disease processes are most affected, we found expected changes in lysosomal proteins (p = 8.98E−12, z = − 2.193), with other NCL proteins being upregulated as previously described^32,33^ (Fig. 4). Other disease-associated processes included accumulation of lipid (p = 1.38E4, z = − 3.229), immune response of cells and quantity of CD8 positive cells (p = 4.3E5, z = 3.252), glial morphology (p = 1.21E−09, z = − 0.68), neurodegenerative changes (p = 8.03E5, z = 0.813), movement disorders (p = 5.93E6, z = − 1.664), abnormal myelination (p = 8.71E−10, z = 0.898) and sensory system development (p = 7.39E4, z = 0.83) (Fig. 4, Supplementary Table S4).

Overall, analyzing proteins whose expression changed by 1.2-fold or more at 3 months in *Ppt1*^*−/−*^ spinal cord revealed significant inflammatory changes, particularly a large humoral immune response. While the effect of infiltrating lymphocytes on the *Ppt1*^*−/−*^ mouse brain has been well documented^34,35^, this only occurred much later in disease progression.

To validate this proteomic finding of comparatively early spinal peripheral lymphocyte infiltration, we immunostained WT and *Ppt1*^*−/−*^ spinal cords sections at 3 and 7 months simultaneously for peripheral lymphocyte markers CD4 and CD8^35^. This staining showed positive lymphocytes in in the grey and white matter of *Ppt1*^*−/−*^ spinal cords, even at the early 3 month timepoint (Supplementary Figure S1), confirming the changes seen in the proteomic analysis. This is significantly earlier than similar changes are observed in the brains of these mice^35^. Furthermore, proteomic analysis revealed protein changes associated with abnormal myelination, which we confirmed by QFWB analysis of MBP (Supplementary Figure S2), as well as other pathophysiological mechanisms including nervous system development and movement disorders that have not been previously described for this disease. These serve to not only demonstrate the relatively early onset of pathology in this region of the CNS, but may also help understand the cause of such vulnerability.

#### Early vs. late changes in the *Ppt1*^*−/−*^ spinal cord

**Figure 5:**
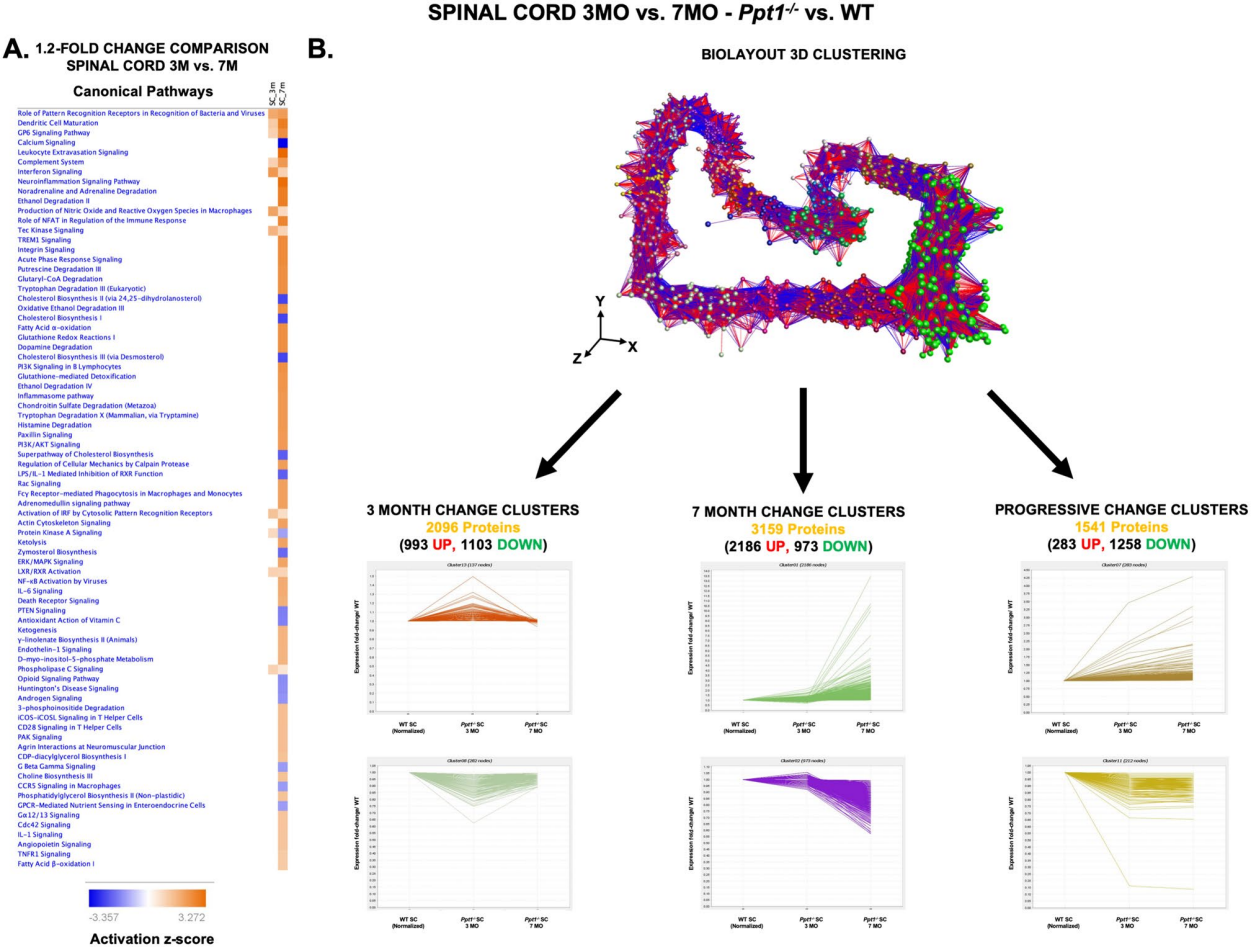
Early vs. late proteomic changes in the spinal cord of *Ppt1*^*−/−*^ mice. (**A**) Comparative proteomic profiling of differentially expressed proteins by 1.2 fold (20%) in the spinal cord of *Ppt1*^*−/−*^ mice at 3 (early) and 7 (late) months of age respectively revealed an overall increase in the number and degree of change in the later timepoints. However, some pathways were more affected at 3 months of age including interferon signalling (3 month SC z = 2.236–3 month SC, 7 month SC z = 1.0), Production of Nitric Oxide and Reactive Oxygen Species in Macrophages (3 month SC z = 2.0-, 7 month SC z = 0.78-), Protein Kinase A signalling (3 month SC z = 0.81, 7 month SC z = − 1.22) and TEC Kinase signalling (3 month SC z = 1.63, 7 month SC z = 0.94). (**B**) *BioLayout Express 3D* clustering representation of proteomic expression data for 3- and 7-month-old *Ppt1*^*−/−*^ spinal cords, and a normalised WT group. Each sphere or node represents a single protein, and edges or lines represent the relatedness of proteins to one another, in this case, based on similarity in expression profile. Applying the Markov Clustering Algorithm in *BioLayout Express 3D* clusters these nodes based on similarity in expression profile. As a result, proteins exhibiting similar expression trends to one another are not only grouped closely in spatial proximity within the graph but also are coloured identically to represent membership within the same cluster. The expression profile behind each cluster can then be individually analysed to identify and isolate those that show more change at 3 months of age, 7 months of age or those that show a similar/progressive change.

We next analyzed *Ppt1*^*−/−*^ vs. wildtype spinal cords at 7 months, using the 1.2-fold cut-off as above. This yielded 1,441 (924 up- and 217 downregulated) (Fig. 2B) differentially expressed proteins (compared to 249 at 3 months), indicative of the progressive worsening phenotype of CLN1 disease in the spinal cord. This analysis revealed that the major canonical pathways and cellular networks affected at disease end-stage differ from those identified at the early symptomatic stage at 3 months, including Integrin signalling (p = 3.80E−11), cholesterol biosynthesis (p = 3.21E−10), Rho GTPase signalling (p = 2.83E−07), ILK Signaling (p = 3.66 E−07) and opioid signalling pathways (p = 4.67E−07) (Supplementary Figure S3, Supplementary Table S10). In order to compare affected pathways between 3 and 7 month timepoints, we used *IPA* software to generate a comparative heatmap of altered canonical pathways revealing many more affected pathways with disease progression (Fig. 5A). While this might be expected, several processes including interferon signalling (3 month z = 2.236, 7 month z = 1.0), Production of Nitric Oxide and Reactive Oxygen Species in Macrophages (3 month z = 2.0, 7 month z = 0.78), Protein Kinase A signalling (3 month z = 0.81-, 7 month z = − 1.22) and TEC Kinase signalling (3 month z = 1.63, 7 month SC z = 0.94) were all unexpectedly more pronounced at 3 months of age. These data indicate that there is not simply a worsening of common phenotypes with progressing disease, but rather that there are certain pathological processes that are more florid at early symptomatic stages in the *Ppt1*^*−/−*^ cord, and whose involvement decreases with age.

Studying those proteins whose expression ratio changed by 1.2-fold (20%) or more highlights the most significant pathological changes at each time point. However, to dissect which of these processes are distinct or common between 3 and 7 months in *Ppt1*^*−/−*^ cords, we also analyzed these datasets using *BioLayout Express 3D*^16–18,25–28^. We input the expression ratios (*Ppt1*^*−/−*^/WT) of 7,160 proteins from LC–MS without a filtering cut-off for 3 and 7 month-old spinal cords (Fig. 2B). Individual protein clusters were grouped based on trends of expression as those that showed more changes at (a) 3 months (2,096 proteins); (b) 7 months (3,159 proteins); or (c) clusters that showed progressive or similar changes between timepoints (1,541 proteins) (Fig. 5B). *IPA* software was then used for a functional cellular pathway analysis of each group.

**Figure 6:**
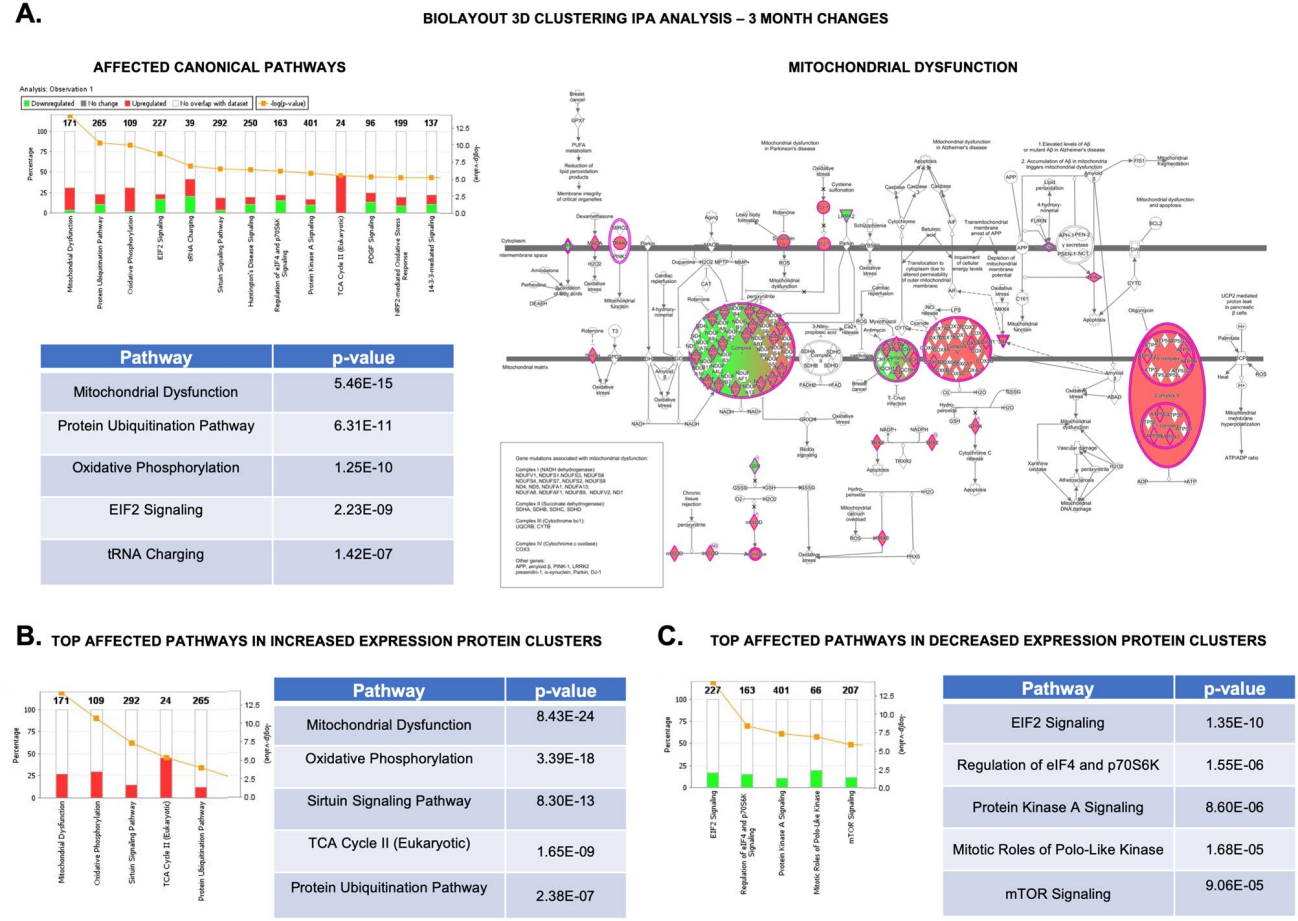
*Biolayout express 3D* clustering analysis of changes in the spinal cord of *Ppt1*^*−/−*^ mice at 3 months of age. Top affected canonical pathways from the analysis of (**A**) the differentially expressed protein clusters that showed increased and decreased expression at 3-month-old *Ppt1*^*−/−*^ cords, compared to normalized WT and 7-month-old cord. Mitochondrial dysfunction, the top affected pathway is highlighted to show affected proteins (red/green). IPA analysis of only those protein clusters showing increased expression (**B**) or decreased expression (**C**) at 3-month-old *Ppt1*^*−/−*^ spinal cords, compared to normalized WT and 7-month-old spinal cord revealed differentially affected canonical pathways. (See Supplementary Tables S5, S6, S7 and Supplementary Excel File for further details.)

*IPA* analysis of changes of *BioLayout Express 3D* clusters predominant at the 3 month timepoint, revealed a surprising enrichment of proteins associated with mitochondrial dysfunction (p = 5.46E−15), involving complexes I, III, IV and V (Fig. 6A, Supplementary Table S5), protein ubiquitination (p = 6.31E−11), oxidative phosphorylation (p = 1.25E−10), EIF2 signaling (p = 2.23E−09) and tRNA charging (p = 1.42E−07). We then looked at which cellular functions and diseases were enriched within this dataset—revealing a predicted increase in organismal death (p = 1.11E−12, z = 3.644) and mortality (p = 3.98E−12, z = 3.565), dysfunction of neurons, (p = 4.29E−05, z = 2.164), benign lesions (p = 0.000307, z = 2.0) and a decrease in development of cytoplasm (p = 2.81E−12, z = − 3.095) and cytoskeleton (p = 2.03E−09, z = − 2.85), formation of filaments (p = 2.03E−09, z = − 2.85), potentiation of the synapse (p = 5.01E−08, z = − 2.521) and microtubule dynamics (p = 1.95E−14, z = − 2.124) (Supplementary Figure S4A, Supplementary Table S11). There were also changes involving development of the CNS (p = 1.23E−6, z = − 2.035) and development of the synapse (p = 3.63E−08, z = 0.451).

Further dividing the *BioLayout 3D* clusters predominantly altered in the 3 month *Ppt1*^*−/−*^ spinal cord into those proteins that showed increased (993 proteins) or decreased expression (1,103 proteins) revealed that while mitochondrial dysfunction and oxidative phosphorylation changes were the main canonical pathways associated with increased expression in the 3 month *Ppt1*^*−/−*^ spinal cord in these protein clusters (p = 8.43E−24, 3.39E−18, respectively) (Fig. 6B, Supplementary Table S6), the main pathways associated with decreased expression were EIF2 signalling, eIF4 and p70S6K regulation, Protein Kinase A Signalling, Polo-Like Kinase and mTOR signalling (p = 1.35E−10, 1.55E−06, 8.60E−06, 1.68E−05, 9.06E−05, respectively) (Fig. 6C, Supplementary Table S6). These findings are of particular importance as they highlight early and distinct disease-related changes in the CLN1 spinal cord that decrease with age. These changes are possibly indicative of either altered postnatal maturation processes^32^, and/or increased cell turnover due to cell loss in the 3 month old *Ppt1*^*−/−*^ spinal cord (Fig. 1).

Similarly, *IPA* analysis of *BioLayout 3D* clusters with progressive or similar changes at 3 and 7 months showed RAC signalling, Axonal guidance, Rho family GTPase signalling, Stathmin1 regulation of breast cancer and ARP-WASP complex1 to be significantly altered (p = 7.09E−10, 1.19E−09, 2.91E−09, 3.84E−08, 1.03E−07, respectively) (Supplementary Figure S4B, Supplementary Table S11). Interestingly, these clusters were primarily comprised of proteins that were downregulated. *IPA* analysis of protein clusters that show changes at 7 months of age (late in the disease) showed that the integrin signalling, Rho family GTPase signalling, FAK signalling, Rho GDI signalling and insulin receptor signalling were significantly altered (p = 1.87E−17, 4.00E−12 31.0, 1.26E−11, 1.91E−11, 2.00E−11 respectively) (Supplementary Figure S4C, Supplementary Table S12).

Together, our comparative proteomic analyses reveal multiple pathological processes occurring in the *Ppt1*^*−/−*^ mouse spinal cord at 3 months, highlighting its significant involvement relatively early in disease. Interestingly, dissecting up- and down-regulated processes revealed the involvement of distinct early disease-related changes including mitochondrial dysfunction, cancer pathways, mTOR signalling and developmental processes. While these have previously been shown to be linked to PPT1 function in vitro^33,36^, and our data confirms this alteration in vivo for the first time at a significantly earlier timepoint than previously predicted. Early involvement of these cellular processes that play a role in spinal cord development and/or function may further help explain spinal cord vulnerability in CLN1 disease.

#### Comparing *Ppt1*^***−/−***^ cortical and spinal cord changes highlights conserved and divergent mechanisms

Although significant pathology is already underway in their spinal cord at 3 months, the cortex of *Ppt1*^*−/−*^ mice shows no significant glial activation, accumulation of storage material or neuron loss at this timepoint^11,12^. Therefore, having elucidated various altered cellular processes associated with spinal cord pathology, we compared the proteomic changes that occur in the spinal cord at 3 months with changes seen in the cortex at this age. Despite the obvious cytoarchitectural and biochemical differences between these tissues, such a comparison would reveal common and distinct mechanisms responsible for regional CLN1 disease progression.

**Figure 7:**
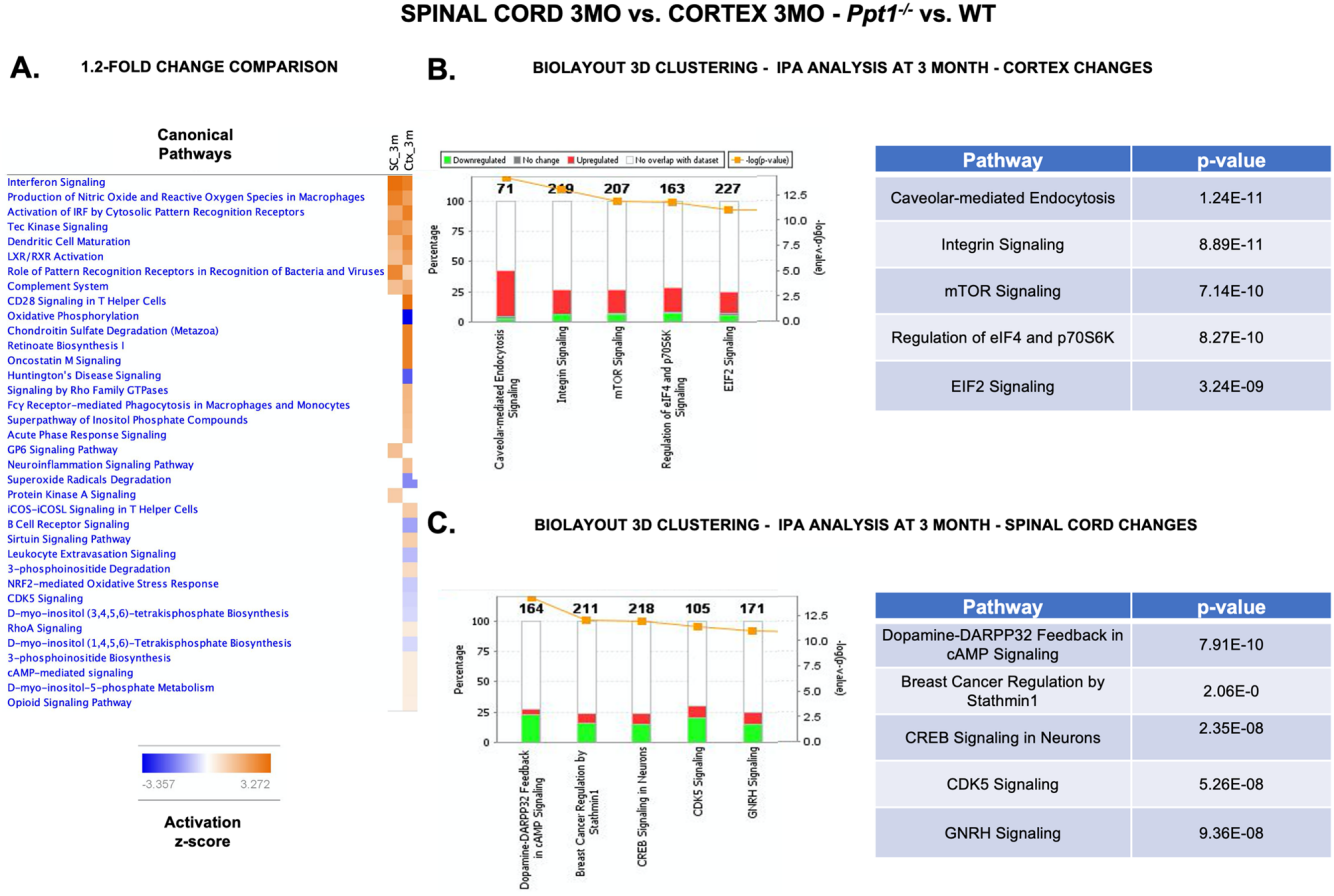
Cortical vs. spinal cord proteomic changes in 3-month-old *Ppt1*^*−/−*^ mice. (A) Analysis of the differentially expressed proteins by 1.2 fold (20%) in the spinal cord and cortex of *Ppt1*^*−/−*^ mice revealed a greater number of affected pathways in the cortex, with some pathways more affected in the spinal cord including—interferon signalling (z = 2.236-SC, 2.0-Ctx), Production of nitric oxide and reactive oxygen species in macrophages (z = 2.0-SC, 1.5-Ctx), pattern recognition receptors for bacteria and viruses (z = 1.89-SC, 0.7-Ctx), Tec Kinase signalling (z = 1.63-SC, 1.41-Ctx), GP6 Signalling Pathway (z = 1 SC, 0-Ctx) and complement system (z = 1-SC,0-Ctx). *BioLayout Express 3D* clustering analysis of the expression trends between 3-month-old *Ppt1*^*−/−*^ mouse cortex, spinal cord and normalized WT control (see methods) revealed the top affected canonical pathways in the cortex (**B**) and spinal cord (**C**), respectively. (See Supplementary Tables S8, S9 and Supplementary Excel File for further details.)

We first filtered proteins from the cortex at 3 months that showed a 1.2-fold (20%) change in *Ppt1*^*−/−*^ tissue. This yielded 705 proteins (343 up- and 362 downregulated) (compared to 249 proteins with a similar change in the 3 month cord) (Fig. 2B). While this may be due to greater coverage of cortical proteins with LC–MS and *IPA* analysis, there was a significant overlap of affected cellular processes between the cortex and spinal cord. Top affected canonical pathways included LXR/RXR Activation, autophagy, mitochondrial dysfunction, oxidative phosphorylation and phagosome maturation (p = 1.04E−06, 2.78E−06, 6.33E−06, 6.87E−06 and 1.46E−05, respectively) (Supplementary Figure S5, Supplementary Table S13). Similarly, the cellular networks most affected included Cell Morphology, Cellular Assembly and Organization, Carbohydrate Metabolism (*IPA*-score = 52), Metabolic Disease, Cellular Compromise, Embryonic Development (*IPA*-score = 42), Energy Production, Molecular Transport, Nucleic Acid Metabolism (*IPA*-score = 39), Cardiovascular System Development and Function, Embryonic Development, Organ Development (*IPA*-score = 39) and Cellular Development, Post-Translational Modification, Hair and Skin Development (*IPA*-score = 39) (Supplementary Figure S5, Supplementary Table S14). These data highlight the great similarity in the most affected pathways in the cortex and spinal cord, with the cortex showing a higher expression fold change. The degree of change in cortical protein expression was particularly striking as there is little histologically detectable pathology observed at this stage in this brain region^11,12^. However, a marked exception to this broad similarity between regions was the degree of altered inflammatory pathways, especially the humoral immune response pathways, revealing a potential cause for spinal cord vulnerability. This was further highlighted when we compared affected canonical pathways between 3 month cortices and spinal cords (Fig. 7A), where affected pathways including interferon signalling (z = 2.236-SC, 2.0-Ctx), Production of nitric oxide and reactive oxygen species in macrophages (z = 2.0-SC, 1.5-Ctx), pattern recognition receptors for bacteria and viruses (z = 1.89-SC, 0.7-Ctx), Tec Kinase signalling (z = 1.63-SC, 1.41-Ctx), GP6 Signalling Pathway (z = 1-SC, 0-Ctx) and complement system (z = 1-SC,0-Ctx) were all more affected in the cord than in the cortex of *Ppt1*^*−/−*^ mice at 3 months.

Using *BioLayoutExpress 3D*^16–18,25–28^, we inputted expression ratios (*Ppt1*^*−/−*^/WT) of 7,160 proteins without a cut-off for the cortex and spinal cord at 3 months respectively (Fig. 2B). We then clustered proteins based on whether these changes occurred more in the spinal cord (2,341 Proteins), cortex (2,371 Proteins) or both regions (1,179 Proteins). Analysis of clusters most impacted in the cortex revealed affected canonical pathways included caveolar-mediated endocytosis signalling, Integrin signalling, mTOR signalling, regulation of eIF4 and p70S6K signalling and EIF 2 signalling (p = 1.24E−1, 8.89E−11, 7.14E−10, 8.27E−10, 3.24E−09, respectively) (Fig. 7B, Supplementary Table S8). Many of these processes were also shown to be altered in the 3 month spinal cord, but to a lesser extent, highlighting the common pathological processes between these regions. Analyzing protein clusters that showed more changes in the spinal cord revealed affected canonical pathways-Dopamine-DARPP32 Feedback in cAMP Signaling (p = 7.91E−10), Breast Cancer Regulation by Stathmin1 (p = 2.06E−08), CREB Signaling in Neurons (p = 2.35E−08), CDK5 Signaling (p = 5.26E−08), GNRH Signaling (p = 9.36E−08), indicating that these cell signaling pathways may be uniquely dysregulated in the spinal cord (Fig. 7C, Supplementary Table S10).We then analyzed clusters for similar protein expression changes between the spinal cord and cortex, revealing common pathways—Synaptic Long Term Potentiation (p = 6.10E−07), G Beta Gamma Signaling (p = 1.05E−06), Opioid signaling Pathway (p = 6.19E−06), CREB Signaling in Neurons (p = 1.11E−05) and adrenergic Signaling (p = 2.46E−05), further highlighting the synaptic defects observed early in disease in these mice^34,35^, also revealing novel cell-signalling pathways (Supplementary Figure S6, Supplementary Table S15).

Overall, these analyses show similarities in the cellular pathways impacted by disease in the cortex and spinal cord of *Ppt1*^*−/−*^ mice. Despite a lesser extent of histopathological phenotypes, the cortex showed a greater degree of change in terms of protein expression in these shared pathways as compared to the spinal cord. We cannot discount the differences in cytoarchitecture or cellular function between the cortex and spinal cord as influencing these data. Nevertheless, the extent of overlap of changes seen between the cortex and spinal cords of *Ppt1*^*−/−*^ mice demonstrate the significance of cellular processes that precede neurodegeneration. However, the most conspicuous difference we detected was the degree of inflammatory changes, particularly humoral immune response in the *Ppt1*^*−/−*^ spinal cord as compared to the cortex at this relatively early stage of disease, in addition to revealing the presence of unique cell signalling pathways within the spinal cord.

## Discussion

CLN1 disease is a rapidly progressing form of NCL with no curative therapy available^1,5^. Despite the success of enzyme replacement therapy for CLN2 disease^37^, brain-directed therapies have not been as effective for CLN1 disease^1,5^. We recently showed significant spinal pathology in CLN1 disease patients and *Ppt1*^*−/−*^ mice, which contributes to disease outcome and unexpectedly precedes the onset of brain pathology^9^. Therefore, in this study, we firstly characterized the nature and progression of CLN1 disease in the spinal cords in more detail, showing significant early changes in regional volume, glial activation and interneuron number in these mice at 3 months of age. We then elucidated disease mechanisms that may explain the regional differences in the CNS of *Ppt1*^*−/−*^ mice.

Histopathological analysis of *Ppt1*^*−/−*^ mouse spinal cords at 3 and 7 months of age revealed progressive changes in regional volume that affect both the grey and white matter, a significant activation of astrocytes and microglia, and a selective loss of interneuron populations before motor neurons. This spinal pathology affected both dorsal and ventral horns and also affected different levels of the spinal cord to a similar extent.

The mouse spinal cord is fully developed by 3 months^38^. However, as the *Ppt1*^*−/−*^ spinal cord is already smaller in volume at this age, it raises the possibility that the spinal cord may not have developed normally. Whether this is due to defects in embryonic development or post-natal maturation is yet to be determined and may help explain this regional vulnerability.

The activation of microglia and astrocytes is a more accurate predictor of neuron loss in the NCLs than the accumulation of storage material^12,39^. Given the inherent defects of *Ppt1*^*−/−*^ mouse cortical astrocytes and microglia in vitro and their impact on neuron survival^40^, as well as the importance of neuroinflammatory changes in other neurodegenerative pathologies^41,42^, our data provide evidence for a distinct pathogenic role of glial activation in regional vulnerability in CLN1 disease. However, this awaits further experimental validation in vivo and it will be important to characterize the inflammatory networks involved in the early pathogenesis of CLN1 disease. To achieve this goal a comprehensive analysis of early cytokine and chemokine changes, in addition to defining the activation states of astrocytes and microglia and the role they play in regional neurodegeneration is currently ongoing.

The *Ppt1*^*−/−*^ mouse brain shows also selective neuron loss, with interneurons and large complex neurons such as lamina V cortical neurons and cerebellar Purkinje cells particularly vulnerable^12,13^. While *Ppt1*^*−/−*^ mouse spinal cords show significant overall neuron loss at 3 months^9^, our data now show spinal interneurons are lost significantly before motor neurons in the *Ppt1*^*−/−*^ spinal cord. To date this is the earliest significant cell loss seen in these mice, highlighting the severity of spinal pathology and revealing a different pattern of cellular vulnerability to other NCLs^43^ and to diseases such as amyotrophic lateral sclerosis (ALS) and other motor neuron diseases (MNDs)^44–47^. Therefore, it will be critical to study the precise relationship between glial activation and neuron loss in the CLN1 disease spinal cord, and investigations into neuron-glial interactions in this especially vulnerable region of the CNS are currently ongoing. Together, our histopathological characterization has emphasized the extent and significant progression of spinal pathology in the cord of *Ppt1*^*−/−*^ mice, that precedes brain pathology and has identified spinal interneurons and glia as being particularly involved early in this disease.

We next undertook a comparative proteomic investigation of *Ppt1*^*−/−*^ and wildtype mouse spinal cords to investigate the mechanisms driving spinal cord vulnerability in CLN1 disease. Our comparison of proteins altered in 3 month old cords revealed significant alterations in autophagy, phagocytosis and neuroinflammatory processes. In particular, there was a profound humoral immune response in the *Ppt1*^*−/−*^ spinal cord which is progressive until 7 months. The status of the blood brain barrier (BBB) has previously been detailed in CLN1 disease^35,48^, with CD4 and CD8 + ve lymphocytes, seen infiltrating the *Ppt1*^*−/−*^ mouse optic nerve and brain as early as 3 months^35^. Taking into account our proteomic findings, we validated these findings by immunostaining 3 and 7 month spinal cord sections at the same time for both CD4 and CD8 + ve lymphocytes. This revealed increased infiltration of these cells at 3 months of age, significantly earlier than similar changes occur in the brains of these mice^35^. Unlike the BBB, the blood-spinal cord-barrier (BSCB) is relatively more porous and therefore potentially more vulnerable to lymphocyte infiltration^49–51^. However, the BSCB has yet to be studied in any form of NCL, and our data suggest performing a detailed investigation. Interestingly, the expression of PPT1 is most abundant in CD8 + ve dendritic cells and microglia in mice, with comparable expression in humans (www.biogps.org (2020))^52^. Therefore, as we have documented inherent defects in microglia and astrocytes^40^, peripheral blood lymphocytes may be also vulnerable to CLN1 disease and require further characterization.

We also demonstrated alterations in a variety of cellular processes including mitochondrial dysfunction, oxidative phosphorylation and TCA Cycle function, EIF2, EIF4 and p70SK, mTOR pathway. These alterations have previously been described in CLN1 disease either in vitro in neuroblastoma cells^33^ and cancer cell lines^36^ or in a CLN1 mouse model that bears a different disease-causing mutation (*Ppt1*^*△ex4*^)^32^. Our data confirm these shared pathological processes also occur in the more commonly used *Ppt1*^*−/−*^ mice, and that these pathological changes are ongoing at this relatively early time point in the spinal cord. Despite having specific disease-causing mutations and the unique pathomechanisms discussed here, the *Ppt1*^*−/−*^ mouse cortex and spinal cord also show alterations in biological processes that are significantly affected in a host of neurodegenerative conditions^53–56^. Such common pathogenic mechanisms may prove critical in understanding the downstream cellular consequences of lysosomal pathology and may also prove to be amenable to therapeutic intervention. Given the regional vulnerability of the spinal cord, cell populations within the cord may be more vulnerable to alterations in these processes than other cell types, however, this awaits experimental confirmation.

Given marked differences in the onset of histologically detectable pathology in the spinal cord and cortex of *Ppt1*^*−/−*^ mice^9,12^, proteomic profiling revealed surprisingly similar changes in protein expression in these two CNS regions at 3 months of age. Despite the cortex being relatively spared in terms of glial activation and neuron loss at 3 months, there were many similar processes altered in this brain region as in the spinal cord and many of these were to a greater extent than in the cord. While this may be in part due to inherent differences in cellular and biochemical constitution, these data reiterate the similarity in disease processes across the CNS regions and shift the focus upon regional and cell-type vulnerability to these alterations. However, the most conspicuous difference between the *Ppt1*^*−/−*^ mouse cortex and spinal cord at 3 months was the remarkable extent of immune response present in the cord at this early stage of disease, and work into characterizing the exact nature and progression of these changes in currently ongoing. As already discussed, whether this is due to inherent cell-type defects or as a reaction to regional cues is yet to be determined, but these data help us understand the cause of such regional differences in vulnerability. Furthermore, our data raise the possibility of spinal cord targeted regional therapeutic interventions such as anti-inflammatories or neuroprotective agents^57,58^ to improve disease outcome.

Taken together, this study provides a thorough characterization of early symptomatic spinal cord pathology in CLN1 disease, and using comparative proteomic profiling, elucidates significantly affected cellular mechanisms that contribute to this early regional vulnerability. In particular, we highlight spinal interneurons and glial cells as the earliest observed vulnerable cell populations. Early alterations in cell-signalling, developmental processes, mitochondrial dysfunction and a significant spinal cord immune response may play a major role in disease progression and regional vulnerability in *Ppt1*^*−/−*^ mice. It is now apparent that CLN1 disease does not just affect the brain. Characterizing and subsequently targeting the early inflammatory response observed in *Ppt1*^*−/−*^ spinal cords may therefore prove to be an effective strategy either alone or in combination with other therapies, as has become increasingly common across the NCLs^57,59,60^. Such characterization of the pathomechanisms of CLN1 disease and other forms of NCLs can directly inform therapeutic efforts in order to significantly improve disease outcomes.

## Methods

### Animals

The *Ppt1*^*−/−*^ mouse was created by targeted gene disruption that eliminates the last exon of the *Ppt1/Cln1* gene^61^. These mice were backcrossed with C57BL/6 mice for more than 10 generations and maintained as a homozygous breeding stock. *Ppt1*^*−/−*^ and age-matched C57BL/6 control mice were bred and housed in a barrier facility at the Institute of Psychiatry, Psychology and Neuroscience, King’s College London (London, UK). All procedures were approved by the Denmark Hill Animal Welfare and Ethics Body and carried out under the Animals (Scientific) Procedures Act, 1986 (UK) (Project Licences 70/6567 and 70/7364).

### Immunohistochemistry

#### Histological tissue processing

Mice at 3 and 7 months of age were anesthetized with sodium pentobarbitone (100 mg/kg) and perfused transcardially with heparinised PBS followed by 4% formaldehyde in PBS, pH 7.4. Whole mice were post-fixed in this 4% formaldehyde solution for a further 48 h before being transferred to a 50 mM Tris solution (TBS). Spinal cords were separated from the brainstem from the dorsal aspect, just below the foramen magnum. The entire spinal column was then dissected out from the surrounding tissue, and the spinal cord was dissected from the column by initial dorsal laminectomy followed by separation and removal of individual vertebrae. The spinal cords were cryoprotected in 30% sucrose in 50 mM TBS, pH 7.6. 40 µm coronal sections were then cut on a Microm HM430 freezing microtome (Microm International GmbH, Wallendorf, Germany).

#### Cresyl fast violet staining (Nissl staining)

Cresyl fast violet staining was performed by mounting a one in 24 series of sections on to chrome-gelatin coated slides and leaving them to air-dry overnight at room temperature. The slides were then incubated in 0.1% cresyl fast violet solution with 0.05% acetic acid (VWR) overnight, before being differentiated by passing through a graded series of Industrial Methylated Spirit (IMS) solutions (70%, 80%, 90% and 2 × 100%), before being cleared in Xylene (VWR) and coverslipped in DPX (VWR).

#### Immunohistochemical staining

A one in 24 series of spinal cord sections from each animal were immunostained free-floating for markers of astrocytosis (rabbit anti-GFAP, 1:8,000 dilution, Dako Ltd); microglial activation (rat anti-mouse CD68, 1:2,000 dilution, AbD Serotec); interneurons (rabbit anti-Calbindin, 1:10,000 dilution, Swant; rabbit anti-Calretinin, 1:5,000 dilution, Swant), motor neurons (rat anti-CD71, 1:200 dilution, AbD Serotec); and peripheral lymphocyte markers (rat anti-CD4, 1:50 dilution, AbD Serotec, Rat anti-CD8, 1:100 dilution, AbD Serotec). Endogenous peroxidase activity in the sections was quenched with 1% H_2_O_2_ in TBS with 0.3% Triton-X100 (TBS-T), washed and then blocked in 15% normal serum (Vector Laboratories) diluted in TBS-T. The species of normal serum was directed against the host species of the secondary antibody. After blocking, sections were incubated overnight at 4 °C in primary antibody diluted in 10% normal serum in TBS-T. Sections were then washed and incubated at room temperature in biotinylated secondary antibody at 1:1,000 dilution (biotinylated swine anti-rabbit IgG, DAKO; biotinylated rabbit anti-rat IgG, Vector Laboratories), followed by washing and incubation in Vectastain Elite ABC kit (1:1,000, Vector Laboratories) before visualization with 3,3′-diaminobenzidine tetrahydrochloride (DAB) (Sigma). Sections were then mounted on to chrome-gelatin coated slides, air-dried overnight, cleared in xylene (VWR) and coverslipped in DPX.

#### Measurement of regional volume

Regional volumes were measured as previously described^10^, where whole spinal cord volumes and spinal cord grey matter volumes were measured in a one in 24 series of sections for each spinal cord. Using the Cavalieri estimator, estimates of volume in cubic µm were obtained by superimposing a 125 μm grid on Nissl-stained sections and using *Stereo Investigator* software (Microbrightfield Inc, Williston, Vermont) linked to a Zeiss Axioskop 2 MOT (Zeiss, Germany) with a DAGE-MTI CCD-100 camera (Dage-MTI, Michigan City, Iowa). White matter volumes were obtained as a subtractive value of the whole volume and grey matter volume of each spinal cord section, with reference to a spinal cord atlas^15^.

#### Thresholding image analysis

To analyze glial activation in the grey matter of sections stained for GFAP and CD68, as well as neuron density in Laminae I-III of sections stained for interneuron markers, we performed thresholding image analysis using *Image Pro Premier* software (Media Cybernetics, Chicago, IL, USA). Here, 30 non-overlapping images from 3 sections at 40× magnification for the glial markers and 50 non-overlapping images from 5 sections at 63× magnification for interneuron markers, were captured for each defined region, with all parameters of light intensity, camera setup and calibrations being kept constant. An appropriate threshold was applied to each set of images so as to select the positive foreground immunoreactivity over the background. Results were obtained as an average percentage area of positive staining per image^62^.

#### Interneuron and motor neuron counts

Due to the relatively low numbers of interneurons, stereological methods prove inefficient for counting them^63^. Counts for neurons in Laminae IV–X in a one in 24 series of sections immunostained for interneuron markers (Calbindin, Calretinin) and motor neuron markers (CD-71), was carried out manually using unbiased sampling. Each section was visualized at 10× magnification using a Axioplan microscope (Zeiss) and a live video camera (Luminera Infinity 3URM Colour camera), with *Image Pro Premier* software (Media Cybernetics, Chicago, IL, USA). Total estimates were obtained as the product of the counted cells, periodicity and the number of intervals.

### Proteomic processing

#### Protein extraction for liquid chromatography-with tandem mass tagged-mass spectrometry (LC–MS/MS)

Mice at 3 and 7 months of age were euthanized in a CO_2_ chamber, followed by isolation of the spinal column with scissors at the base of skull and at the pelvic bone. The skull was then dissected to remove the brain and the cortices were isolated. A 20 ml syringe with TBS and an 18G needle was then inserted into the rostral end of the vertebral column, ensuring a tight fit. The spinal cord was then extruded by hydraulic pressure into a glass petri dish containing TBS as previously described^64^. Collected cortices and spinal cords were then frozen at − 80 °C prior to proteomic analysis.

Sample preparation for Tandem–Mass–Tag proteomic analysis was carried out as previously described^16,17^. Briefly, samples were homogenized in extraction buffer [100 mM Tris–HCl (pH7.6) 4% (w/v) SDS] containing 1% protease cocktail inhibitor (Thermo Fisher, UK). Post homogenization, samples were spun at 300×*g* for 2 min and left on ice for 20 min. Homogenates were transferred to Lo-Bind 1.5 ml tubes (Sigma Aldrich) and centrifuged at 20,000×*g* for 20 min at 4 °C with the soluble fraction of each sample then transferred to new Lo-Bind tubes. Protein determination using the bicinchoninic acid assay (BCA) (Pierce, UK) was carried out according to manufacturer’s guidelines. Samples were then pooled into groups for a single tissue region at a single timepoint per genotype (n = 4 or 5 mice) as follows – 3 month wild type (WT) cortex, 3 month *Ppt1*^−/−^ cortex, 3 month WT spinal cord, 3 month *Ppt1*^−/−^ spinal cord, 7 month WT cortex, 7 month *Ppt1*^−/−^ cortex, 7 month WT spinal cord and 7 month *Ppt1*^−/−^ spinal cord (https://doi.org/10.7488/ds/2750 (2020)). Individual samples containing 20 μg of protein per sample within each group, i.e., equivalent amounts of protein from all samples—were pooled to generate a condition specific master sample of 100 μg protein.

TMT proteomic analysis of the pooled samples was performed by the FingerPrints Proteomics facilities at the University of Dundee. Pooling according to sample type allows a reduction in potential noise in the system generated through inter individual differences, subtle post-mortem handling differences and/or sample isolation. The inclusion of an equivalent proportion of each individual sample into a readily comparable pool allows the generation of a molecular fingerprint representative of each condition and enables subsequent analysis of individual patient variability in the resulting validatory work (as a deviation from the population signal, as previously described^17,18,65,66^). Indeed, we have previously demonstrated that pooling can enable the reliable identification of up to 3× more proteins in complex samples^26^.

Sample processing was carried out as follows: protein samples were thawed, trypsinized and desalted at room temperature. 100 μg of desalted tryptic peptides per sample were dissolved in 100 μl of 100 mM tetraethylammonium bromide (TEAB). The 8 different tandem mass tag (TMT) labels comprising the TMT8plex kit (Thermo Fisher Scientific) were dissolved in 41 μl anhydrous acetonitrile. Each dissolved label was added to a different sample—3 month WT cortex (Tag 127N), 3 month *Ppt1*^−/−^ cortex (Tag 127C), 3 month WT spinal cord (Tag 129N), 3 month *Ppt1*^−/−^ spinal cord (Tag 129C), 7 month WT cortex (Tag 128N), 7 month *Ppt1*^−/−^ cortex (Tag 128C), 7 month WT spinal cord (Tag 130N )and 7 month *Ppt1*^−/−^ spinal cord (130C) (https://doi.org/10.7488/ds/2750 (2020)). The sample-label mixture was incubated for 1 h at room temperature. Labelling reaction was stopped by adding 8 μl of 5% hydroxylamine per pooled sample.

As per^16,17^, following labelling with TMT, pooled samples were desalted, and dried in a speed-vac at 30 °C, re-dissolved in 200 μl ammonium formate (10 mM, pH 10) and peptides were fractionated using an Ultimate 3,000 High Performance Liquid Chromatography column (Thermo-Scientific) containing an XBridge C18 column (XBridge peptide BEH, 130 Å, 3.5 μm, 2.1 × 150 mm) (Waters, Ireland) with an XBridge guard column (XBridge, C18, 3.5 μm, 2.1 × 10mm) (Waters, Ireland). Buffers A (10 mM ammonium formate in milliQ water) and B (10 mM ammonium formate with 90% acetonitrile) were adjusted to pH 10 with ammonia. Fractions were collected using a WPS-3000FC auto-sampler (Thermo-Scientific) at 1 min intervals. Column and guard column were equilibrated for 20 min at a constant flow rate of 0.2 ml/min. 175 μl per sample was loaded onto the column at a rate of 0.2 ml/min, and the separation gradient was started 1 min after sample was loaded onto the column. Peptides were eluted from the column with a gradient of 2–5% Buffer B in 6 min, and then from 5 to 60% Buffer B in 50 min. The column was washed for 16 min in Buffer B and re-equilibrated at 2%. The fraction collection started 1 min after injection and stopped after 80 min (total 80 fractions, 200 μl each). The total number of fractions concatenated was set to 15 and the content of the fractions was dried and suspended in 50 μl of 1% formic acid prior to analysis with LC–MS/MS.

#### LC–MS/MS analysis

Liquid chromatography–tandem mass spectrometry was performed by FingerPrints Proteomics Facilities at the University of Dundee, as per^16,17^. Briefly, analysis of peptide readout was performed on a *Q Exactive* HF Hybrid Quadrupole-Orbitrap Mass Spectrometer (Thermo Scientific) coupled with a Dionex Ultimate 3000 RS (Thermo Scientific). LC buffers were made up to the following: Buffer A [2% acetonitrile and 0.1% formic acid in Milli-Q water (v/v)] and Buffer B (80% acetonitrile and 0.08% formic acid in Milli-Q water (v/v). Aliquots of 15 μl per sample were loaded at a rate of 5 μl/min onto a trap column (100 μm × 2 cm, PepMap nanoViper C18 column, 5 μm, 100 Å, Thermo Scientific) which was equilibrated with 98% Buffer A. The trap column was washed for 6 min at the same flow rate and then the trap column was switched in-line with a resolving C18 column (Thermo Scientific) (75 μm × 50 cm, PepMap RSLC C18 column, 2 μm, 100 Å). Peptides were eluted from the column at a constant flow rate of 300 nl/min with a linear gradient from 95% Buffer A to 40% Buffer B in 122 min, and then to 98% Buffer B by 132 min. The resolving column was then washed with 95% Buffer B for 15 min and re-equilibrated in 98% Buffer A for 32 min. Q Exactive HF was used in data dependent mode. A scan cycle was comprised of a MS1 scan (m/z range from 335 to 1,800, with a maximum ion injection time of 50 ms, a resolution of 120,000 and automatic gain control (AGC) value of 3 × 106) followed by 15 sequential-dependent MS2 scans (with an isolation window set to 0.4 Da, resolution at 60,000, maximum ion injection time at 200 ms and AGC 1 × 105. To ensure mass accuracy, the mass spectrometer was calibrated on the first day that the runs were performed.

#### Protein identification

Raw MS data were searched against mouse (*Mus musculus*) protein sequences from *UniProtKB/Swiss-Prot* using the *MASCOT* search engine (Matrix Science, Version 2.2) through *Proteome Discoverer* software (Version 1.4, Thermo Fisher). Parameters for database search were as follows: MS1 Tolerance: 10 ppm; MS2 Tolerance: 0.06da; fixed modification: Carbamidomethyl (C) Variable Modification: Oxidation (M), Dioxidation (M), Acetyl (N-term), Gln- > pyro-Glu (N-term Q), TMT 10(N-term and K); maximum missed cleavage: 2; and target FDR 0.01. All identifications were quantified as relative ratios of expression of *Ppt1*^*−/−*^ to wildtype tissue at each time point for each tissue. Of these, six groups—3 month WT cortex, 3 month *Ppt1*^−/−^ cortex, 3 month WT spinal cord, 3 month *Ppt1*^−/−^ spinal cord, 7 month WT spinal cord and 7 month *Ppt1*^−/−^ spinal cord were taken forward for further analysis. Relative abundance ratios along with *UnitProtKB/Swiss-Prot* identifications were exported into *Microsoft Excel* as a raw data file containing ID, ratio of change in expression at each time point (Data available here: https://doi.org/10.7488/ds/2750 (2020)).

#### Quantitative fluorescent western blotting (QFWB)

QFWB was also done as previously described^18,67^. Briefly, samples were denatured in *NuPage* LDS Sample buffer 4X (Invitrogen, UK) at 98 °C and 10ug of protein loaded and run on 4–20% TGX Stain-free gels (Bio-Rad, Hercules, CA). Accuracy of loading and protein estimation was confirmed by total protein analysis of TGX stain-free gels after exposure to UV light in a *ChemiDoc* Imager as per manufacturer’s instructions (Bio-Rad) . Protein transfer to a polyvinylidene fluoride (PDVF) membrane was carried out using the *TransBlot Turbo* system using manufacturer recommended transfer packs *(Bio-Rad)*. Membranes were incubated with Odyssey blocking buffer (Li-Cor) for 1 h. Next, membranes were incubated in primary antibodies (Rabbit anti-SNAP25, 1:4,000 , Abcam; Rat anti-myelin basic protein (MBP), 1:500, EMD Millipore (18–20 kD band analyzed as per manufacturer’s product data sheet); Rabbit anti-COXIV, 1:2000, Abcam; Mouse anti-glial fibrillary acidic protein (GFAP), 1:2000, Sigma-Aldrich; Rabbit anti-calbindin, 1:2000,Swant; Mouse anti-synaptophysin, 1:8,000, Enzo Life sciences and Mouse anti-glutamine synthetase, 1:4,000, BD Biosciences) overnight at 4 °C and secondary antibodies (goat anti-rabbit 680 nm, 1:4,000, goat anti-rat 800 nm and goat anti-mouse 800 nm, RD) for 2 h at room temperature. Visualization and analysis was carried out with the *ChemiDoc* Imager and *Image Lab* software (Bio-Rad). Quantification was performed on single channels with the analysis software provided.

#### Biolayout express 3D

Unfiltered lists comprised of protein IDs with corresponding expression ratios at each time point relative to the equivalent timepoint wildtype value (available here: https://doi.org/10.7488/ds/2750 (2020)) were imported separately into *BioLayout Express 3D* and clustered based on relative expression profile in two separate comparisons—(a) 3 month *Ppt1*^*−/−*^ spinal cord vs. 7 month *Ppt1*^*−/−*^ spinal cord; and (b) or 3 month *Ppt1*^*−/−*^ spinal cord vs. 3 month *Ppt1*^*−/−*^ cortex. Algorithms in *BioLayout Express 3D* generate a visual network to represent each data set, in this study utilizing spatial proximity to represent the similarity in expression profile between nodes^25^. It was therefore possible to isolate clusters of proteins grouped by similarity in terms of expression profiles over time. The resultant visual networks were utilized to distinguish expression clusters that followed trends in expression that favored either group or both groups (3 vs. 7 month spinal cord analysis—altered only at 3 months, altered only at 7 months and similar/progressive alterations; 3 month cortex vs. 3 month spinal cord analysis—differential detection only in cortex, differential detection only in spinal cord and similar alterations in both cortex and spinal cord at 3 months). These clusters were analyzed and input into *Ingenuity Pathway Analysis* (Ingenuity Systems).

#### Ingenuity pathway analysis (IPA)

The *IPA* software (Ingenuity systems) contains a library of biological pathways published in the literature that are ranked by the significance of the association between the dataset and the canonical pathway. This significance is defined by two parameters: (a) the ratio of the number of proteins from the input dataset that are pertaining to a particular pathway divided by the total number of genes ascribed by the Ingenuity Knowledge Database to that canonical pathway and (b) a P value calculated using Fischer’s test that determines whether the probability of association between component proteins in the input dataset and the canonical pathway are due to chance. Prediction activation scores (z score) are a statistical measure of the match between an expected relationship direction and the observed protein expression within the input dataset. A positive z score indicates activation while a negative z score indicate inhibitiony^16,18,68^. A 1.2 fold-change (20%) threshold filter was applied in *IPA* to the datasets for 3 month cortex, 3 month spinal cord and 7 month spinal cord (*Ppt1*^*−/−*^ vs. wildtype respectively), analyzed and observed interactions were selected for this analysis.

#### Statistical analysis

All measurements for histological processing were performed blind to genotype. Statistical analysis for histological measurements of regional volumes, neuron counts, cell areas and thresholding image analysis as well as for QFWB analysis between two groups at a given time-point was calculated by multiple two-tailed, unpaired, parametric t test with Bonferroni-Dunn correction where p ≤ 0.05 was considered significant using GraphPad Prism version 8.0.0 for MacOS (GraphPad Software, San Diego, CA, www.graphpad.com (2020)).

## Supplementary information

Supplementary file1

Supplementary file2

Supplementary file3
