# Supplementary material for: Comparative proteomic profiling reveals mechanisms for early spinal cord vulnerability in CLN1 disease
Source: Sci Rep. 2020 Sep 16;10:15157. doi: 10.1038/s41598-020-72075-7 (PMC7495486; doi:10.1038/s41598-020-72075-7)
Supplement: Supplementary file 1 — Supplementary file1 [file 41598_2020_72075_MOESM1_ESM.pdf]

DAVID Bioinformatics Resources 6.8
Laboratory of Human Retrovirology and Immunoinformatics (LHRI)

*** Welcome to DAVID 6.8 ***
*** If you are looking for DAVID 6.7, please visit our development site. ***

Functional Annotation Chart

Help and Manual

Current Gene List: List_1
Current Background: Mus musculus
7237 DAVID IDs

Options

Thresholds: **Count** 2 **EASE** 0.1
Display: Fold Enrichment Bonferroni Benjamini FDR Fisher Exact LT,PH,PT # of Records 1000

Rerun Using Options | Create Sublist

387 chart records

Download File

Sublist	Category	Term	RT	Genes	Count	%	P-Value	Benjamini
	GOTERM_CC_DIRECT	cytoplasm	RT		3395	46.9	1.1E-246	1.5E-243
	GOTERM_CC_DIRECT	extracellular exosome	RT		1651	22.8	6.8E-202	4.3E-199
	GOTERM_CC_DIRECT	mitochondrion	RT		1160	16.0	6.7E-180	2.8E-177
	GOTERM_CC_DIRECT	cytosol	RT		1134	15.7	8.7E-146	2.8E-143
	GOTERM_CC_DIRECT	membrane	RT		3263	45.1	2.7E-139	6.9E-137
	GOTERM_CC_DIRECT	synapse	RT		368	5.1	3.0E-68	6.4E-66
	GOTERM_CC_DIRECT	cell-cell adherens junction	RT		254	3.5	2.1E-61	3.7E-59
	GOTERM_CC_DIRECT	intracellular ribonucleoprotein complex	RT		252	3.5	1.1E-57	1.7E-55
	GOTERM_CC_DIRECT	focal adhesion	RT		290	4.0	2.0E-56	2.8E-54
	GOTERM_CC_DIRECT	neuron projection	RT		305	4.2	5.9E-56	7.5E-54
	GOTERM_CC_DIRECT	mitochondrial inner membrane	RT		287	4.0	7.8E-56	9.1E-54
	GOTERM_CC_DIRECT	neuronal cell body	RT		364	5.0	1.3E-55	1.4E-53
	GOTERM_CC_DIRECT	myelin sheath	RT		172	2.4	2.7E-55	2.7E-53
	GOTERM_CC_DIRECT	endosome	RT		364	5.0	1.5E-52	1.3E-50
	GOTERM_CC_DIRECT	cell junction	RT		447	6.2	2.8E-51	2.4E-49
	GOTERM_CC_DIRECT	postsynaptic density	RT		192	2.7	2.2E-46	1.8E-44
	GOTERM_CC_DIRECT	axon	RT		264	3.6	4.3E-46	3.2E-44
	GOTERM_CC_DIRECT	perinuclear region of cytoplasm	RT		424	5.9	4.6E-46	3.3E-44
	GOTERM_CC_DIRECT	dendrite	RT		323	4.5	1.5E-44	1.0E-42
	GOTERM_CC_DIRECT	nucleoplasm	RT		956	13.2	1.2E-42	7.6E-41
	GOTERM_CC_DIRECT	cytoskeleton	RT		598	8.3	2.7E-39	1.6E-37
	GOTERM_CC_DIRECT	endoplasmic reticulum	RT		686	9.5	1.8E-38	1.0E-36
	GOTERM_CC_DIRECT	ribosome	RT		152	2.1	2.1E-37	1.2E-35
	GOTERM_CC_DIRECT	cytoplasmic vesicle	RT		375	5.2	1.3E-33	6.9E-32
	GOTERM_CC_DIRECT	Golgi apparatus	RT		613	8.5	2.8E-33	1.5E-31
	GOTERM_CC_DIRECT	mitochondrial matrix	RT		145	2.0	1.4E-31	7.1E-30
	GOTERM_CC_DIRECT	cell projection	RT		398	5.5	7.6E-31	3.6E-29
	GOTERM_CC_DIRECT	dendritic spine	RT		119	1.6	5.7E-29	2.6E-27
	GOTERM_CC_DIRECT	protein complex	RT		354	4.9	2.0E-28	8.9E-27
	GOTERM_CC_DIRECT	spliceosomal complex	RT		111	1.5	4.1E-28	1.8E-26
	GOTERM_CC_DIRECT	synaptic vesicle	RT		103	1.4	3.2E-26	1.3E-24
	GOTERM_CC_DIRECT	lamellipodium	RT		124	1.7	1.0E-25	4.1E-24
	GOTERM_CC_DIRECT	growth cone	RT		120	1.7	8.8E-25	3.4E-23
	GOTERM_CC_DIRECT	intracellular membrane-bounded organelle	RT		398	5.5	1.0E-24	3.8E-23
	GOTERM_CC_DIRECT	microtubule	RT		202	2.8	6.8E-23	2.5E-21
	GOTERM_CC_DIRECT	nucleus	RT		2410	33.3	7.5E-23	2.7E-21
	GOTERM_CC_DIRECT	postsynaptic membrane	RT		148	2.0	1.6E-21	5.5E-20
	GOTERM_CC_DIRECT	terminal bouton	RT		89	1.2	1.1E-20	3.6E-19
	GOTERM_CC_DIRECT	trans-Golgi network	RT		115	1.6	1.6E-19	5.2E-18
	GOTERM_CC_DIRECT	catalytic step 2 spliceosome	RT		77	1.1	7.9E-19	2.5E-17
	GOTERM_CC_DIRECT	proteasome complex	RT		58	0.8	7.5E-18	2.3E-16
	GOTERM_CC_DIRECT	cell-cell junction	RT		129	1.8	7.0E-17	3.3E-15
	GOTERM_CC_DIRECT	melanosome	RT		77	1.1	1.4E-16	3.3E-15
	GOTERM_CC_DIRECT	endoplasmic reticulum membrane	RT		354	4.9	2.2E-16	6.4E-15
	GOTERM_CC_DIRECT	nuclear membrane	RT		140	1.9	3.5E-16	9.4E-15
	GOTERM_CC_DIRECT	lysosomal membrane	RT		143	2.0	9.1E-16	2.5E-14
	GOTERM_CC_DIRECT	early endosome	RT		139	1.9	1.1E-15	2.7E-14
	GOTERM_CC_DIRECT	ruffle membrane	RT		63	0.9	3.2E-15	8.3E-14
	GOTERM_CC_DIRECT	late endosome	RT		91	1.3	3.9E-15	1.0E-13
	GOTERM_CC_DIRECT	mitochondrial outer membrane	RT		102	1.4	5.3E-15	1.4E-13
	GOTERM_CC_DIRECT	endosome membrane	RT		86	1.2	6.3E-15	1.6E-13
	GOTERM_CC_DIRECT	actin cytoskeleton	RT		125	1.7	1.0E-14	2.5E-13
	GOTERM_CC_DIRECT	presynaptic membrane	RT		60	0.8	1.2E-14	2.9E-13
	GOTERM_CC_DIRECT	lysosome	RT		184	2.5	2.8E-14	6.6E-13
	GOTERM_CC_DIRECT	nucleolus	RT		400	5.5	3.6E-14	8.4E-13
	GOTERM_CC_DIRECT	early endosome membrane	RT		66	0.9	4.7E-14	1.1E-12
	GOTERM_CC_DIRECT	respiratory chain	RT		49	0.7	1.0E-13	2.3E-12
	GOTERM_CC_DIRECT	mitochondrial membrane	RT		73	1.0	1.2E-13	2.6E-12
	GOTERM_CC_DIRECT	Golgi membrane	RT		214	3.0	1.2E-13	2.5E-12
	GOTERM_CC_DIRECT	nuclear pore	RT		52	0.7	3.1E-13	6.5E-12
	GOTERM_CC_DIRECT	membrane raft	RT		150	2.1	3.4E-13	7.2E-12
	GOTERM_CC_DIRECT	clathrin-coated vesicle	RT		49	0.7	3.6E-13	7.3E-12
	GOTERM_CC_DIRECT	extrinsic component of membrane	RT		63	0.9	1.1E-12	2.3E-11
	GOTERM_CC_DIRECT	SNARE complex	RT		42	0.6	3.2E-12	6.4E-11
	GOTERM_CC_DIRECT	dendritic shaft	RT		49	0.7	3.6E-12	7.1E-11
	GOTERM_CC_DIRECT	perikaryon	RT		95	1.3	4.5E-12	8.8E-11
	GOTERM_CC_DIRECT	nuclear envelope	RT		98	1.4	1.2E-11	2.3E-10
	GOTERM_CC_DIRECT	intracellular	RT		685	9.5	1.5E-11	2.9E-10
	GOTERM_CC_DIRECT	mitochondrial respiratory chain complex I	RT		40	0.6	1.8E-11	3.4E-10
	GOTERM_CC_DIRECT	cell body	RT		70	1.0	1.9E-11	3.5E-10

Sublist	Category	Term	RT	Genes	Count	%	P-Value	Benjamini
	GOTERM_CC_DIRECT	synaptic vesicle membrane	RT		48	0.7	2.4E-11	4.2E-10
	GOTERM_CC_DIRECT	nuclear speck	RT		119	1.6	3.3E-11	5.9E-10
	GOTERM_CC_DIRECT	recycling endosome	RT		76	1.1	7.4E-11	1.3E-9
	GOTERM_CC_DIRECT	actin filament	RT		51	0.7	1.1E-10	1.8E-9
	GOTERM_CC_DIRECT	peroxisome	RT		83	1.1	1.1E-10	1.9E-9
	GOTERM_CC_DIRECT	ruffle	RT		65	0.9	1.3E-10	2.3E-9
	GOTERM_CC_DIRECT	excitatory synapse	RT		33	0.5	1.4E-10	2.4E-9
	GOTERM_CC_DIRECT	late endosome membrane	RT		61	0.8	2.4E-10	3.9E-9
	GOTERM_CC_DIRECT	endoplasmic reticulum-Golgi intermediate compartment	RT		50	0.7	5.3E-10	8.5E-9
	GOTERM_CC_DIRECT	vesicle	RT		97	1.3	6.4E-10	1.0E-8
	GOTERM_CC_DIRECT	smooth endoplasmic reticulum	RT		27	0.4	1.5E-9	2.4E-8
	GOTERM_CC_DIRECT	filopodium	RT		54	0.7	1.6E-9	2.4E-8
	GOTERM_CC_DIRECT	autophagosome	RT		47	0.6	2.0E-9	3.2E-8
	GOTERM_CC_DIRECT	endomembrane system	RT		72	1.0	2.7E-9	4.1E-8
	GOTERM_CC_DIRECT	cell cortex	RT		86	1.2	3.4E-9	5.1E-8
	GOTERM_CC_DIRECT	presynapse	RT		46	0.6	4.2E-9	6.3E-8
	GOTERM_CC_DIRECT	polysome	RT		35	0.5	4.3E-9	6.3E-8
	GOTERM_CC_DIRECT	stress fiber	RT		48	0.7	5.0E-9	7.2E-8
	GOTERM_CC_DIRECT	Z disc	RT		76	1.1	6.3E-9	9.1E-8
	GOTERM_CC_DIRECT	presynaptic active zone	RT		27	0.4	7.5E-9	1.1E-7
	GOTERM_CC_DIRECT	extracellular vesicle	RT		38	0.5	8.5E-9	1.2E-7
	GOTERM_CC_DIRECT	cytoplasmic vesicle membrane	RT		73	1.0	2.0E-8	2.8E-7
	GOTERM_CC_DIRECT	recycling endosome membrane	RT		34	0.5	2.8E-8	3.8E-7
	GOTERM_CC_DIRECT	clathrin-coated pit	RT		43	0.6	3.6E-8	4.9E-7
	GOTERM_CC_DIRECT	caveola	RT		56	0.8	4.3E-8	5.8E-7
	GOTERM_CC_DIRECT	mitochondrial small ribosomal subunit	RT		25	0.3	4.6E-8	6.1E-7
	GOTERM_CC_DIRECT	synaptic membrane	RT		28	0.4	4.7E-8	6.1E-7
	GOTERM_CC_DIRECT	peroxisomal membrane	RT		39	0.5	5.8E-8	7.6E-7
	GOTERM_CC_DIRECT	microtubule organizing center	RT		88	1.2	6.1E-8	7.8E-7
	GOTERM_CC_DIRECT	microtubule cytoskeleton	RT		89	1.2	8.3E-8	1.1E-6
	GOTERM_CC_DIRECT	mitochondrial intermembrane space	RT		50	0.7	8.7E-8	1.1E-6
	GOTERM_CC_DIRECT	phagocytic vesicle	RT		32	0.4	1.3E-7	1.7E-6
	GOTERM_CC_DIRECT	axon terminus	RT		55	0.8	1.4E-7	1.8E-6
	GOTERM_CC_DIRECT	neuromuscular junction	RT		44	0.6	1.6E-7	2.0E-6
	GOTERM_CC_DIRECT	small ribosomal subunit	RT		25	0.3	1.8E-7	2.2E-6
	GOTERM_CC_DIRECT	intercalated disc	RT		37	0.5	2.5E-7	3.0E-6
	GOTERM_CC_DIRECT	midbody	RT		75	1.0	2.7E-7	3.2E-6
	GOTERM_CC_DIRECT	basolateral plasma membrane	RT		107	1.5	3.6E-7	4.2E-6
	GOTERM_CC_DIRECT	cytoplasmic, membrane-bounded vesicle	RT		84	1.2	4.1E-7	4.8E-6
	GOTERM_CC_DIRECT	axonal growth cone	RT		24	0.3	4.2E-7	4.9E-6
	GOTERM_CC_DIRECT	sarcolemma	RT		69	1.0	4.3E-7	5.0E-6
	GOTERM_CC_DIRECT	proteasome accessory complex	RT		17	0.2	5.9E-7	6.7E-6
	GOTERM_CC_DIRECT	membrane coat	RT		25	0.3	5.9E-7	6.7E-6
	GOTERM_CC_DIRECT	mitochondrial ribosome	RT		22	0.3	6.7E-7	7.5E-6
	GOTERM_CC_DIRECT	inclusion body	RT		18	0.2	1.4E-6	1.5E-5
	GOTERM_CC_DIRECT	cortical cytoskeleton	RT		25	0.3	1.7E-6	1.9E-5
	GOTERM_CC_DIRECT	mitochondrial large ribosomal subunit	RT		28	0.4	2.9E-6	3.1E-5
	GOTERM_CC_DIRECT	cytoplasmic ribonucleoprotein granule	RT		23	0.3	3.1E-6	3.4E-5
	GOTERM_CC_DIRECT	ubiquitin ligase complex	RT		54	0.7	3.2E-6	3.5E-5
	GOTERM_CC_DIRECT	centrosome	RT		199	2.7	3.4E-6	3.6E-5
	GOTERM_CC_DIRECT	mitochondrial proton-transporting ATP synthase complex	RT		17	0.2	3.5E-6	3.7E-5
	GOTERM_CC_DIRECT	nuclear matrix	RT		57	0.8	4.1E-6	4.3E-5
	GOTERM_CC_DIRECT	adherens junction	RT		37	0.5	4.2E-6	4.3E-5
	GOTERM_CC_DIRECT	cytosolic small ribosomal subunit	RT		36	0.5	4.2E-6	4.3E-5
	GOTERM_CC_DIRECT	extracellular matrix	RT		141	1.9	5.4E-6	5.5E-5
	GOTERM_CC_DIRECT	proteasome core complex	RT		18	0.2	6.2E-6	6.3E-5
	GOTERM_CC_DIRECT	cell leading edge	RT		37	0.5	7.7E-6	7.8E-5
	GOTERM_CC_DIRECT	cell	RT		114	1.6	9.2E-6	9.2E-5
	GOTERM_CC_DIRECT	viral nucleocapsid	RT		19	0.3	9.2E-6	9.1E-5
	GOTERM_CC_DIRECT	phagocytic vesicle membrane	RT		32	0.4	1.5E-5	1.5E-4
	GOTERM_CC_DIRECT	cytoplasmic mRNA processing body	RT		45	0.6	1.5E-5	1.5E-4
	GOTERM_CC_DIRECT	inhibitory synapse	RT		18	0.2	2.2E-5	2.1E-4
	GOTERM_CC_DIRECT	eukaryotic translation initiation factor 3 complex	RT		15	0.2	2.3E-5	2.2E-4
	GOTERM_CC_DIRECT	podosome	RT		24	0.3	2.4E-5	2.3E-4
	GOTERM_CC_DIRECT	axon cytoplasm	RT		24	0.3	2.4E-5	2.3E-4
	GOTERM_CC_DIRECT	heterotrimeric G-protein complex	RT		27	0.4	2.8E-5	2.7E-4
	GOTERM_CC_DIRECT	mitochondrial nucleoid	RT		31	0.4	2.9E-5	2.8E-4
	GOTERM_CC_DIRECT	HOPS complex	RT		13	0.2	3.0E-5	2.8E-4
	GOTERM_CC_DIRECT	microtubule associated complex	RT		21	0.3	4.1E-5	3.8E-4
	GOTERM_CC_DIRECT	endocytic vesicle	RT		35	0.5	4.6E-5	4.2E-4
	GOTERM_CC_DIRECT	nuclear periphery	RT		17	0.2	5.1E-5	4.6E-4
	GOTERM_CC_DIRECT	U2 snRNP	RT		17	0.2	5.1E-5	4.6E-4
	GOTERM_CC_DIRECT	COP9 signalosome	RT		24	0.3	5.3E-5	4.8E-4
	GOTERM_CC_DIRECT	apical part of cell	RT		62	0.9	5.6E-5	5.0E-4
	GOTERM_CC_DIRECT	lipid particle	RT		40	0.6	5.6E-5	5.0E-4
	GOTERM_CC_DIRECT	cortical actin cytoskeleton	RT		29	0.4	5.6E-5	5.0E-4
	GOTERM_CC_DIRECT	eukaryotic 48S preinitiation complex	RT		14	0.2	5.7E-5	5.1E-4
	GOTERM_CC_DIRECT	eukaryotic 43S preinitiation complex	RT		14	0.2	5.7E-5	5.1E-4
	GOTERM_CC_DIRECT	dendrite cytoplasm	RT		18	0.2	6.4E-5	5.6E-4
	GOTERM_CC_DIRECT	AP-3 adaptor complex	RT		12	0.2	8.0E-5	6.9E-4
	GOTERM_CC_DIRECT	COPI vesicle coat	RT		12	0.2	8.0E-5	6.9E-4
	GOTERM_CC_DIRECT	cullin-RING ubiquitin ligase complex	RT		12	0.2	8.0E-5	6.9E-4
	GOTERM_CC_DIRECT	BAF-type complex	RT		15	0.2	8.7E-5	7.5E-4
	GOTERM_CC_DIRECT	precatalytic spliceosome	RT		21	0.3	9.5E-5	8.2E-4
	GOTERM_CC_DIRECT	blood microparticle	RT		69	1.0	9.8E-5	8.4E-4
	GOTERM_CC_DIRECT	AMPA glutamate receptor complex	RT		22	0.3	1.0E-4	8.6E-4

Sublist	Category	Term	RT	Genes	Count	%	P-Value	Benjamini
	GOTERM_CC_DIRECT	Golgi stack	RT		24	0.3	1.1E-4	9.1E-4
	GOTERM_CC_DIRECT	axolemma	RT		16	0.2	1.2E-4	9.7E-4
	GOTERM_CC_DIRECT	NuRD complex	RT		16	0.2	1.2E-4	9.7E-4
	GOTERM_CC_DIRECT	T-tubule	RT		35	0.5	1.3E-4	1.1E-3
	GOTERM_CC_DIRECT	voltage-gated potassium channel complex	RT		45	0.6	1.4E-4	1.1E-3
	GOTERM_CC_DIRECT	cell surface	RT		265	3.7	1.5E-4	1.2E-3
	GOTERM_CC_DIRECT	cytoplasmic side of plasma membrane	RT		30	0.4	1.7E-4	1.4E-3
	GOTERM_CC_DIRECT	cytosolic large ribosomal subunit	RT		50	0.7	1.8E-4	1.5E-3
	GOTERM_CC_DIRECT	cis-Golgi network	RT		27	0.4	2.0E-4	1.6E-3
	GOTERM_CC_DIRECT	sarcoplasmic reticulum	RT		35	0.5	2.1E-4	1.6E-3
	GOTERM_CC_DIRECT	node of Ranvier	RT		14	0.2	2.1E-4	1.6E-3
	GOTERM_CC_DIRECT	cytoplasmic stress granule	RT		25	0.3	2.1E-4	1.6E-3
	GOTERM_CC_DIRECT	large ribosomal subunit	RT		15	0.2	2.7E-4	2.1E-3
	GOTERM_CC_DIRECT	protein phosphatase type 2A complex	RT		15	0.2	2.7E-4	2.1E-3
	GOTERM_CC_DIRECT	endoplasmic reticulum lumen	RT		55	0.8	3.0E-4	2.3E-3
	GOTERM_CC_DIRECT	phagocytic cup	RT		16	0.2	3.1E-4	2.4E-3
	GOTERM_CC_DIRECT	neuron projection terminus	RT		16	0.2	3.1E-4	2.4E-3
	GOTERM_CC_DIRECT	proteasome regulatory particle, base subcomplex	RT		12	0.2	3.5E-4	2.7E-3
	GOTERM_CC_DIRECT	axon initial segment	RT		12	0.2	3.5E-4	2.7E-3
	GOTERM_CC_DIRECT	U4/U6 x U5 tri-snRNP complex	RT		18	0.2	3.8E-4	2.9E-3
	GOTERM_CC_DIRECT	preribosome, large subunit precursor	RT		20	0.3	4.1E-4	3.1E-3
	GOTERM_CC_DIRECT	paranode region of axon	RT		13	0.2	4.8E-4	3.7E-3
	GOTERM_CC_DIRECT	apical plasma membrane	RT		145	2.0	5.1E-4	3.9E-3
	GOTERM_CC_DIRECT	proteasome regulatory particle	RT		10	0.1	5.5E-4	4.1E-3
	GOTERM_CC_DIRECT	ciliary rootlet	RT		10	0.1	5.5E-4	4.1E-3
	GOTERM_CC_DIRECT	mitochondrial respiratory chain complex III	RT		10	0.1	5.5E-4	4.1E-3
	GOTERM_CC_DIRECT	U5 snRNP	RT		14	0.2	6.0E-4	4.4E-3
	GOTERM_CC_DIRECT	peroxisomal matrix	RT		14	0.2	6.0E-4	4.4E-3
	GOTERM_CC_DIRECT	basal lamina	RT		15	0.2	6.8E-4	5.0E-3
	GOTERM_CC_DIRECT	apical dendrite	RT		15	0.2	6.8E-4	5.0E-3
	GOTERM_CC_DIRECT	clathrin adaptor complex	RT		15	0.2	6.8E-4	5.0E-3
	GOTERM_CC_DIRECT	exocyst	RT		15	0.2	6.8E-4	5.0E-3
	GOTERM_CC_DIRECT	secretory granule	RT		57	0.8	7.9E-4	5.8E-3
	GOTERM_CC_DIRECT	neuronal cell body membrane	RT		20	0.3	8.0E-4	5.8E-3
	GOTERM_CC_DIRECT	methylosome	RT		11	0.2	8.6E-4	6.2E-3
	GOTERM_CC_DIRECT	transcription export complex	RT		11	0.2	8.6E-4	6.2E-3
	GOTERM_CC_DIRECT	Golgi transport complex	RT		11	0.2	8.6E-4	6.2E-3
	GOTERM_CC_DIRECT	perinuclear endoplasmic reticulum	RT		12	0.2	1.1E-3	8.1E-3
	GOTERM_CC_DIRECT	nBAF complex	RT		12	0.2	1.1E-3	8.1E-3
	GOTERM_CC_DIRECT	spindle	RT		58	0.8	1.2E-3	8.7E-3
	GOTERM_CC_DIRECT	asymmetric synapse	RT		13	0.2	1.3E-3	9.4E-3
	GOTERM_CC_DIRECT	U2-type prespliceosome	RT		13	0.2	1.3E-3	9.4E-3
	GOTERM_CC_DIRECT	semaphorin receptor complex	RT		9	0.1	1.4E-3	1.0E-2
	GOTERM_CC_DIRECT	WASH complex	RT		9	0.1	1.4E-3	1.0E-2
	GOTERM_CC_DIRECT	filopodium membrane	RT		14	0.2	1.5E-3	1.0E-2
	GOTERM_CC_DIRECT	pre-autophagosomal structure	RT		15	0.2	1.5E-3	1.1E-2
	GOTERM_CC_DIRECT	secretory granule membrane	RT		18	0.2	1.6E-3	1.1E-2
	GOTERM_CC_DIRECT	dendrite membrane	RT		18	0.2	1.6E-3	1.1E-2
	GOTERM_CC_DIRECT	brush border	RT		41	0.6	1.7E-3	1.2E-2
	GOTERM_CC_DIRECT	kinetochore	RT		59	0.8	1.8E-3	1.3E-2
	GOTERM_CC_DIRECT	flotillin complex	RT		10	0.1	2.1E-3	1.4E-2
	GOTERM_CC_DIRECT	actomyosin	RT		10	0.1	2.1E-3	1.4E-2
	GOTERM_CC_DIRECT	endoplasmic reticulum tubular network	RT		10	0.1	2.1E-3	1.4E-2
	GOTERM_CC_DIRECT	cell periphery	RT		38	0.5	2.3E-3	1.6E-2
	GOTERM_CC_DIRECT	COPI-coated vesicle	RT		11	0.2	2.5E-3	1.7E-2
	GOTERM_CC_DIRECT	npBAF complex	RT		11	0.2	2.5E-3	1.7E-2
	GOTERM_CC_DIRECT	clathrin-coated vesicle membrane	RT		11	0.2	2.5E-3	1.7E-2
	GOTERM_CC_DIRECT	fascia adherens	RT		11	0.2	2.5E-3	1.7E-2
	GOTERM_CC_DIRECT	SWI/SNF complex	RT		11	0.2	2.5E-3	1.7E-2
	GOTERM_CC_DIRECT	vesicle membrane	RT		20	0.3	2.5E-3	1.7E-2
	GOTERM_CC_DIRECT	exosome (RNase complex)	RT		12	0.2	2.9E-3	1.9E-2
	GOTERM_CC_DIRECT	vacuolar membrane	RT		12	0.2	2.9E-3	1.9E-2
	GOTERM_CC_DIRECT	cell-cell contact zone	RT		13	0.2	3.0E-3	2.0E-2
	GOTERM_CC_DIRECT	retromer complex	RT		16	0.2	3.1E-3	2.0E-2
	GOTERM_CC_DIRECT	U1 snRNP	RT		14	0.2	3.1E-3	2.1E-2
	GOTERM_CC_DIRECT	exon-exon junction complex	RT		15	0.2	3.1E-3	2.0E-2
	GOTERM_CC_DIRECT	cleavage furrow	RT		28	0.4	3.2E-3	2.1E-2
	GOTERM_CC_DIRECT	SCAR complex	RT		8	0.1	3.6E-3	2.3E-2
	GOTERM_CC_DIRECT	growth cone membrane	RT		8	0.1	3.6E-3	2.3E-2
	GOTERM_CC_DIRECT	eukaryotic translation initiation factor 3 complex, eIF3m	RT		8	0.1	3.6E-3	2.3E-2
	GOTERM_CC_DIRECT	VCP-NPL4-UFD1 AAA ATPase complex	RT		8	0.1	3.6E-3	2.3E-2
	GOTERM_CC_DIRECT	pyruvate dehydrogenase complex	RT		8	0.1	3.6E-3	2.3E-2
	GOTERM_CC_DIRECT	nuclear proteasome complex	RT		8	0.1	3.6E-3	2.3E-2
	GOTERM_CC_DIRECT	tRNA-splicing ligase complex	RT		8	0.1	3.6E-3	2.3E-2
	GOTERM_CC_DIRECT	clathrin coat of coated pit	RT		8	0.1	3.6E-3	2.3E-2
	GOTERM_CC_DIRECT	nuclear pore outer ring	RT		9	0.1	4.9E-3	3.1E-2
	GOTERM_CC_DIRECT	U4 snRNP	RT		9	0.1	4.9E-3	3.1E-2
	GOTERM_CC_DIRECT	juxtaparanode region of axon	RT		9	0.1	4.9E-3	3.1E-2
	GOTERM_CC_DIRECT	oligosaccharyltransferase complex	RT		9	0.1	4.9E-3	3.1E-2
	GOTERM_CC_DIRECT	cytosolic proteasome complex	RT		9	0.1	4.9E-3	3.1E-2
	GOTERM_CC_DIRECT	filamentous actin	RT		22	0.3	5.6E-3	3.6E-2
	GOTERM_CC_DIRECT	chloride channel complex	RT		26	0.4	5.6E-3	3.6E-2
	GOTERM_CC_DIRECT	intrinsic component of the cytoplasmic side of the plasma membrane	RT		10	0.1	5.7E-3	3.6E-2
	GOTERM_CC_DIRECT	ER to Golgi transport vesicle membrane	RT		10	0.1	5.7E-3	3.6E-2
	GOTERM_CC_DIRECT	plasma membrane raft	RT		10	0.1	5.7E-3	3.6E-2
	GOTERM_CC_DIRECT	NMDA selective glutamate receptor complex	RT		10	0.1	5.7E-3	3.6E-2

Sublist	Category	Term	RT	Genes	Count	%	P-Value	Benjamini
	GOTERM_CC_DIRECT	endoplasmic reticulum chaperone complex	RT		10	0.1	5.7E-3	3.6E-2
	GOTERM_CC_DIRECT	integral component of mitochondrial outer membrane	RT		15	0.2	5.8E-3	3.7E-2
	GOTERM_CC_DIRECT	nuclear outer membrane	RT		15	0.2	5.8E-3	3.7E-2
	GOTERM_CC_DIRECT	nuclear exosome (RNase complex)	RT		11	0.2	6.1E-3	3.8E-2
	GOTERM_CC_DIRECT	Prp19 complex	RT		11	0.2	6.1E-3	3.8E-2
	GOTERM_CC_DIRECT	dense core granule	RT		11	0.2	6.1E-3	3.8E-2
	GOTERM_CC_DIRECT	pre-autophagosomal structure membrane	RT		12	0.2	6.3E-3	3.9E-2
	GOTERM_CC_DIRECT	U12-type spliceosomal complex	RT		17	0.2	8.7E-3	5.3E-2
	GOTERM_CC_DIRECT	cAMP-dependent protein kinase complex	RT		7	0.1	9.1E-3	5.5E-2
	GOTERM_CC_DIRECT	CORVET complex	RT		7	0.1	9.1E-3	5.5E-2
	GOTERM_CC_DIRECT	aminoacyl-tRNA synthetase multienzyme complex	RT		7	0.1	9.1E-3	5.5E-2
	GOTERM_CC_DIRECT	microvillus	RT		38	0.5	9.8E-3	5.9E-2
	GOTERM_CC_DIRECT	cytoplasmic microtubule	RT		30	0.4	1.0E-2	6.1E-2
	GOTERM_CC_DIRECT	chaperonin-containing T-complex	RT		8	0.1	1.1E-2	6.7E-2
	GOTERM_CC_DIRECT	AP-2 adaptor complex	RT		8	0.1	1.1E-2	6.7E-2
	GOTERM_CC_DIRECT	ESCRT III complex	RT		8	0.1	1.1E-2	6.7E-2
	GOTERM_CC_DIRECT	azurophil granule	RT		8	0.1	1.1E-2	6.7E-2
	GOTERM_CC_DIRECT	small nuclear ribonucleoprotein complex	RT		8	0.1	1.1E-2	6.7E-2
	GOTERM_CC_DIRECT	dynactin complex	RT		8	0.1	1.1E-2	6.7E-2
	GOTERM_CC_DIRECT	Golgi cis cisterna	RT		8	0.1	1.1E-2	6.7E-2
	GOTERM_CC_DIRECT	integral component of mitochondrial inner membrane	RT		13	0.2	1.2E-2	6.9E-2
	GOTERM_CC_DIRECT	lamellipodium membrane	RT		13	0.2	1.2E-2	6.9E-2
	GOTERM_CC_DIRECT	trans-Golgi network transport vesicle	RT		12	0.2	1.2E-2	7.2E-2
	GOTERM_CC_DIRECT	clathrin-coated endocytic vesicle	RT		9	0.1	1.2E-2	7.3E-2
	GOTERM_CC_DIRECT	Arp2/3 protein complex	RT		9	0.1	1.2E-2	7.3E-2
	GOTERM_CC_DIRECT	ionotropic glutamate receptor complex	RT		9	0.1	1.2E-2	7.3E-2
	GOTERM_CC_DIRECT	platelet alpha granule	RT		11	0.2	1.3E-2	7.3E-2
	GOTERM_CC_DIRECT	cell projection membrane	RT		10	0.1	1.3E-2	7.4E-2
	GOTERM_CC_DIRECT	intracellular organelle	RT		10	0.1	1.3E-2	7.4E-2
	GOTERM_CC_DIRECT	zona pellucida receptor complex	RT		10	0.1	1.3E-2	7.4E-2
	GOTERM_CC_DIRECT	cytoplasmic exosome (RNase complex)	RT		10	0.1	1.3E-2	7.4E-2
	GOTERM_CC_DIRECT	MLL1 complex	RT		17	0.2	1.4E-2	8.0E-2
	GOTERM_CC_DIRECT	extrinsic component of cytoplasmic side of plasma membrane	RT		34	0.5	1.5E-2	8.6E-2
	GOTERM_CC_DIRECT	postsynapse	RT		16	0.2	1.5E-2	8.6E-2
	GOTERM_CC_DIRECT	autophagosome membrane	RT		16	0.2	1.5E-2	8.6E-2
	GOTERM_CC_DIRECT	voltage-gated calcium channel complex	RT		15	0.2	1.7E-2	9.4E-2
	GOTERM_CC_DIRECT	lateral plasma membrane	RT		28	0.4	1.7E-2	9.5E-2
	GOTERM_CC_DIRECT	intermediate filament cytoskeleton	RT		27	0.4	1.9E-2	1.0E-1
	GOTERM_CC_DIRECT	cell tip	RT		6	0.1	2.2E-2	1.2E-1
	GOTERM_CC_DIRECT	spliceosomal tri-snRNP complex	RT		6	0.1	2.2E-2	1.2E-1
	GOTERM_CC_DIRECT	chromaffin granule membrane	RT		6	0.1	2.2E-2	1.2E-1
	GOTERM_CC_DIRECT	pICln-Sm protein complex	RT		6	0.1	2.2E-2	1.2E-1
	GOTERM_CC_DIRECT	eukaryotic translation initiation factor 2B complex	RT		6	0.1	2.2E-2	1.2E-1
	GOTERM_CC_DIRECT	myelin sheath adaxonal region	RT		6	0.1	2.2E-2	1.2E-1
	GOTERM_CC_DIRECT	MICOS complex	RT		6	0.1	2.2E-2	1.2E-1
	GOTERM_CC_DIRECT	SMN-Sm protein complex	RT		11	0.2	2.3E-2	1.3E-1
	GOTERM_CC_DIRECT	transport vesicle membrane	RT		16	0.2	2.3E-2	1.3E-1
	GOTERM_CC_DIRECT	mRNA cleavage and polyadenylation specificity factor complex	RT		10	0.1	2.5E-2	1.3E-1
	GOTERM_CC_DIRECT	Golgi cisterna	RT		10	0.1	2.5E-2	1.3E-1
	GOTERM_CC_DIRECT	COPII vesicle coat	RT		7	0.1	2.5E-2	1.4E-1
	GOTERM_CC_DIRECT	somatodendritic compartment	RT		7	0.1	2.5E-2	1.4E-1
	GOTERM_CC_DIRECT	neuron part	RT		7	0.1	2.5E-2	1.4E-1
	GOTERM_CC_DIRECT	signal recognition particle, endoplasmic reticulum targeting	RT		7	0.1	2.5E-2	1.4E-1
	GOTERM_CC_DIRECT	BBSome	RT		7	0.1	2.5E-2	1.4E-1
	GOTERM_CC_DIRECT	holo TFIIH complex	RT		9	0.1	2.6E-2	1.4E-1
	GOTERM_CC_DIRECT	proton-transporting ATP synthase complex, coupling factor F(o)	RT		9	0.1	2.6E-2	1.4E-1
	GOTERM_CC_DIRECT	extrinsic component of mitochondrial inner membrane	RT		9	0.1	2.6E-2	1.4E-1
	GOTERM_CC_DIRECT	Cul2-RING ubiquitin ligase complex	RT		9	0.1	2.6E-2	1.4E-1
	GOTERM_CC_DIRECT	messenger ribonucleoprotein complex	RT		8	0.1	2.6E-2	1.4E-1
	GOTERM_CC_DIRECT	ESCRT I complex	RT		8	0.1	2.6E-2	1.4E-1
	GOTERM_CC_DIRECT	autolysosome	RT		8	0.1	2.6E-2	1.4E-1
	GOTERM_CC_DIRECT	ER to Golgi transport vesicle	RT		14	0.2	2.9E-2	1.5E-1
	GOTERM_CC_DIRECT	telomerase holoenzyme complex	RT		13	0.2	3.2E-2	1.6E-1
	GOTERM_CC_DIRECT	trans-Golgi network membrane	RT		13	0.2	3.2E-2	1.6E-1
	GOTERM_CC_DIRECT	integral component of endoplasmic reticulum membrane	RT		43	0.6	3.3E-2	1.7E-1
	GOTERM_CC_DIRECT	intrinsic component of plasma membrane	RT		19	0.3	3.4E-2	1.8E-1
	GOTERM_CC_DIRECT	Cajal body	RT		24	0.3	3.5E-2	1.8E-1
	GOTERM_CC_DIRECT	costamere	RT		12	0.2	3.5E-2	1.8E-1
	GOTERM_CC_DIRECT	endoplasmic reticulum exit site	RT		12	0.2	3.5E-2	1.8E-1
	GOTERM_CC_DIRECT	apical junction complex	RT		12	0.2	3.5E-2	1.8E-1
	GOTERM_CC_DIRECT	endoplasmic reticulum-Golgi intermediate compartment membrane	RT		21	0.3	3.7E-2	1.9E-1
	GOTERM_CC_DIRECT	sarcoplasmic reticulum membrane	RT		18	0.2	3.8E-2	1.9E-1
	GOTERM_CC_DIRECT	filopodium tip	RT		10	0.1	4.3E-2	2.1E-1
	GOTERM_CC_DIRECT	mitochondrial respiratory chain complex IV	RT		10	0.1	4.3E-2	2.1E-1
	GOTERM_CC_DIRECT	transcriptionally active chromatin	RT		14	0.2	4.3E-2	2.1E-1
	GOTERM_CC_DIRECT	Golgi-associated vesicle	RT		9	0.1	4.7E-2	2.3E-1
	GOTERM_CC_DIRECT	mRNA cap binding complex	RT		9	0.1	4.7E-2	2.3E-1
	GOTERM_CC_DIRECT	beta-catenin destruction complex	RT		9	0.1	4.7E-2	2.3E-1
	GOTERM_CC_DIRECT	nuclear inclusion body	RT		9	0.1	4.7E-2	2.3E-1
	GOTERM_CC_DIRECT	calcium channel complex	RT		13	0.2	4.8E-2	2.3E-1
	GOTERM_CC_DIRECT	potassium channel complex	RT		8	0.1	5.0E-2	2.4E-1
	GOTERM_CC_DIRECT	ER membrane protein complex	RT		8	0.1	5.0E-2	2.4E-1
	GOTERM_CC_DIRECT	CD40 receptor complex	RT		8	0.1	5.0E-2	2.4E-1
	GOTERM_CC_DIRECT	RISC complex	RT		8	0.1	5.0E-2	2.4E-1
	GOTERM_CC_DIRECT	gamma-tubulin complex	RT		8	0.1	5.0E-2	2.4E-1
	GOTERM_CC_DIRECT	immunological synapse	RT		18	0.2	5.2E-2	2.5E-1

Sublist	Category	Term	RT	Genes	Count	%	P-Value	Benjamini
	GOTERM_CC_DIRECT	proteasome regulatory particle, lid subcomplex	RT		7	0.1	5.4E-2	2.5E-1
	GOTERM_CC_DIRECT	insulin-responsive compartment	RT		7	0.1	5.4E-2	2.5E-1
	GOTERM_CC_DIRECT	neurofilament	RT		7	0.1	5.4E-2	2.5E-1
	GOTERM_CC_DIRECT	myelin sheath abaxonal region	RT		7	0.1	5.4E-2	2.5E-1
	GOTERM_CC_DIRECT	fibrillar center	RT		7	0.1	5.4E-2	2.5E-1
	GOTERM_CC_DIRECT	ER-mitochondrion membrane contact site	RT		7	0.1	5.4E-2	2.5E-1
	GOTERM_CC_DIRECT	IkappaB kinase complex	RT		7	0.1	5.4E-2	2.5E-1
	GOTERM_CC_DIRECT	proteasome core complex, alpha-subunit complex	RT		7	0.1	5.4E-2	2.5E-1
	GOTERM_CC_DIRECT	catenin complex	RT		7	0.1	5.4E-2	2.5E-1
	GOTERM_CC_DIRECT	dendritic spine head	RT		7	0.1	5.4E-2	2.5E-1
	GOTERM_CC_DIRECT	MMXD complex	RT		5	0.1	5.4E-2	2.6E-1
	GOTERM_CC_DIRECT	spectrin	RT		5	0.1	5.4E-2	2.6E-1
	GOTERM_CC_DIRECT	proton-transporting ATP synthase complex, catalytic core F(1)	RT		5	0.1	5.4E-2	2.6E-1
	GOTERM_CC_DIRECT	synaptobrevin 2-SNAP-25-syntaxin-1a complex	RT		5	0.1	5.4E-2	2.6E-1
	GOTERM_CC_DIRECT	phosphorylase kinase complex	RT		5	0.1	5.4E-2	2.6E-1
	GOTERM_CC_DIRECT	nuclear pore nuclear basket	RT		5	0.1	5.4E-2	2.6E-1
	GOTERM_CC_DIRECT	extrinsic component of endosome membrane	RT		5	0.1	5.4E-2	2.6E-1
	GOTERM_CC_DIRECT	mitochondrial proton-transporting ATP synthase complex, catalytic core F(1)	RT		5	0.1	5.4E-2	2.6E-1
	GOTERM_CC_DIRECT	eukaryotic translation initiation factor 4F complex	RT		5	0.1	5.4E-2	2.6E-1
	GOTERM_CC_DIRECT	GPI-anchor transamidase complex	RT		5	0.1	5.4E-2	2.6E-1
	GOTERM_CC_DIRECT	septin complex	RT		5	0.1	5.4E-2	2.6E-1
	GOTERM_CC_DIRECT	membrane-bounded organelle	RT		5	0.1	5.4E-2	2.6E-1
	GOTERM_CC_DIRECT	FHF complex	RT		5	0.1	5.4E-2	2.6E-1
	GOTERM_CC_DIRECT	condensed chromosome kinetochore	RT		38	0.5	5.5E-2	2.6E-1
	GOTERM_CC_DIRECT	neuron spine	RT		6	0.1	5.6E-2	2.6E-1
	GOTERM_CC_DIRECT	invadopodium	RT		6	0.1	5.6E-2	2.6E-1
	GOTERM_CC_DIRECT	prefoldin complex	RT		6	0.1	5.6E-2	2.6E-1
	GOTERM_CC_DIRECT	TORC1 complex	RT		6	0.1	5.6E-2	2.6E-1
	GOTERM_CC_DIRECT	cerebellar mossy fiber	RT		6	0.1	5.6E-2	2.6E-1
	GOTERM_CC_DIRECT	fibrinogen complex	RT		6	0.1	5.6E-2	2.6E-1
	GOTERM_CC_DIRECT	paraspeckles	RT		6	0.1	5.6E-2	2.6E-1
	GOTERM_CC_DIRECT	U6 snRNP	RT		6	0.1	5.6E-2	2.6E-1
	GOTERM_CC_DIRECT	clathrin coat	RT		6	0.1	5.6E-2	2.6E-1
	GOTERM_CC_DIRECT	bicellular tight junction	RT		56	0.8	5.9E-2	2.7E-1
	GOTERM_CC_DIRECT	chromatin	RT		54	0.7	6.1E-2	2.8E-1
	GOTERM_CC_DIRECT	mitotic spindle	RT		23	0.3	6.4E-2	2.9E-1
	GOTERM_CC_DIRECT	kinesin complex	RT		25	0.3	6.4E-2	2.9E-1
	GOTERM_CC_DIRECT	nuclear euchromatin	RT		16	0.2	6.6E-2	3.0E-1
	GOTERM_CC_DIRECT	spindle pole	RT		46	0.6	6.8E-2	3.0E-1
	GOTERM_CC_DIRECT	cytoplasmic dynein complex	RT		13	0.2	6.9E-2	3.0E-1
	GOTERM_CC_DIRECT	nuclear inner membrane	RT		20	0.3	7.1E-2	3.1E-1
	GOTERM_CC_DIRECT	pseudopodium	RT		9	0.1	7.6E-2	3.3E-1
	GOTERM_CC_DIRECT	voltage-gated sodium channel complex	RT		9	0.1	7.6E-2	3.3E-1
	GOTERM_CC_DIRECT	vacuolar proton-transporting V-type ATPase complex	RT		9	0.1	7.6E-2	3.3E-1
	GOTERM_CC_DIRECT	Golgi lumen	RT		12	0.2	7.8E-2	3.4E-1
	GOTERM_CC_DIRECT	basement membrane	RT		42	0.6	8.4E-2	3.6E-1
	GOTERM_CC_DIRECT	specific granule	RT		8	0.1	8.5E-2	3.6E-1
	GOTERM_CC_DIRECT	mitochondrial proton-transporting ATP synthase complex, coupling factor F(o)	RT		8	0.1	8.5E-2	3.6E-1
	GOTERM_CC_DIRECT	microtubule plus-end	RT		11	0.2	8.8E-2	3.7E-1
	GOTERM_CC_DIRECT	cytosolic ribosome	RT		11	0.2	8.8E-2	3.7E-1
	GOTERM_CC_DIRECT	histone deacetylase complex	RT		18	0.2	8.9E-2	3.7E-1
	GOTERM_CC_DIRECT	rough endoplasmic reticulum	RT		31	0.4	9.1E-2	3.8E-1
	GOTERM_CC_DIRECT	anaphase-promoting complex	RT		13	0.2	9.5E-2	3.9E-1
	GOTERM_CC_DIRECT	nucleotide-activated protein kinase complex	RT		7	0.1	9.5E-2	3.9E-1
	GOTERM_CC_DIRECT	sodium:potassium-exchanging ATPase complex	RT		7	0.1	9.5E-2	3.9E-1
	GOTERM_CC_DIRECT	ribbon synapse	RT		7	0.1	9.5E-2	3.9E-1
	GOTERM_CC_DIRECT	Cul4A-RING E3 ubiquitin ligase complex	RT		7	0.1	9.5E-2	3.9E-1
	GOTERM_CC_DIRECT	dendritic spine membrane	RT		7	0.1	9.5E-2	3.9E-1
	GOTERM_CC_DIRECT	lysosomal lumen	RT		7	0.1	9.5E-2	3.9E-1

465 gene(s) from your list are not in the output.
