# Supplementary material for: Comparative proteomic profiling reveals mechanisms for early spinal cord vulnerability in CLN1 disease
Source: Sci Rep. 2020 Sep 16;10:15157. doi: 10.1038/s41598-020-72075-7 (PMC7495486; doi:10.1038/s41598-020-72075-7)

**Comparative proteomic profiling reveals mechanisms for early spinal cord vulnerability in CLN1 disease.**

Hemanth R Nelvagal[1,2], Maica Llavero Hurtado[3], Samantha L. Eaton[3], Rachel A. Kline[3], Douglas J Lamont[4], Mark S. Sands[4], Thomas M. Wishart[3], Jonathan D. Cooper[1].

**Contents:**

P2-11: Supplementary figures

P12-26: Supplementary tables

P27-34: Unedited Figures for Main Figure 4 - Disease and Cellular Function.

P35-41: Raw western blot membrane images from main figures

# SUPPLEMENTARY FIGURES

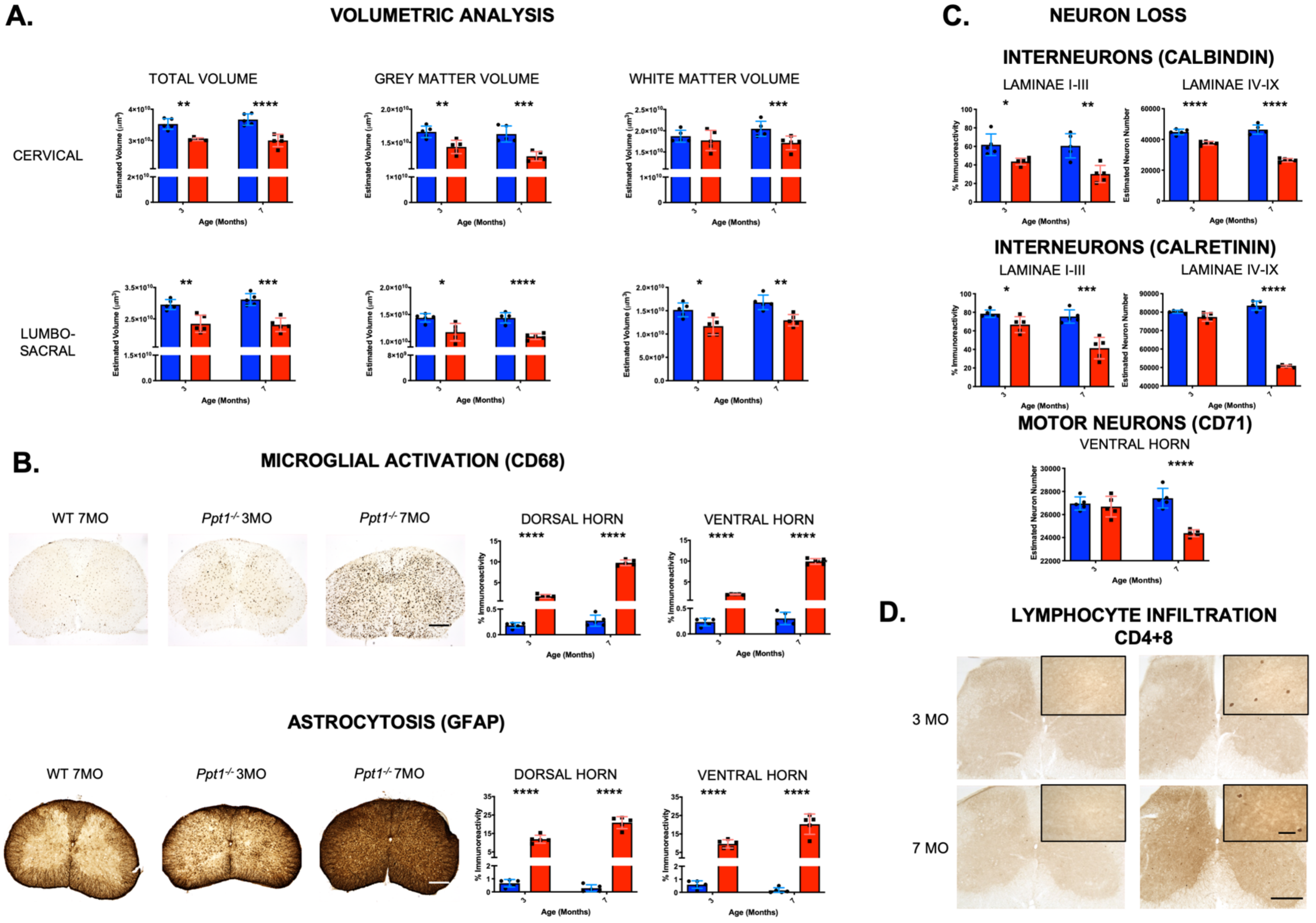

**SUPPLEMENTARY FIGURE 1 – Spinal cord pathology in *Ppt1*$^{-/-}$ mice.**

Stereological analysis of regional volumes (**A**) in the spinal cords reveals significant reduction in total, grey and white matter in the cervical and lumbo-sacral *Ppt1*$^{-/-}$ spinal cords at 3 and 7 months of age to a similar extent. Thresholding imaging analysis (**B**) of sections stained for microglia (CD68) and astrocytes (GFAP) show a significant increase in both markers in the dorsal and ventral horns of the cervical spinal cord at early (3MO) and late (7MO) disease stages in *Ppt1*$^{-/-}$ spinal cords compared to WT. Scale bars= 200µm. Counts of neuron number in the cervical spinal cord (**C**) revealed a significant loss of *Ppt1*$^{-/-}$ mouse spinal interneurons stained with calbindin and calretinin in all laminae [15] as early as 3 months of age, compared to spinal motor neurons (CD-71) which are only significantly lost at the 7 month timepoint. Representative images of spinal cords stained for peripheral lymphocyte markers CD4 and CD8 simultaneously (**D**) reveals increased infiltration of lymphocytes into the *Ppt1*$^{-/-}$ spinal cord as early as 3 months of age (black arrows), compared to WT controls. Scale bars = 200µm (25µm inserts). P-values - *p≤0.05, **p≤0.01, ***p≤0.001, ****p≤0.0001; multiple two-tailed, unpaired, parametric t-test with Bonferroni-Dunn correction Values shown are mean ± SEM. (n = 5 mice/group).

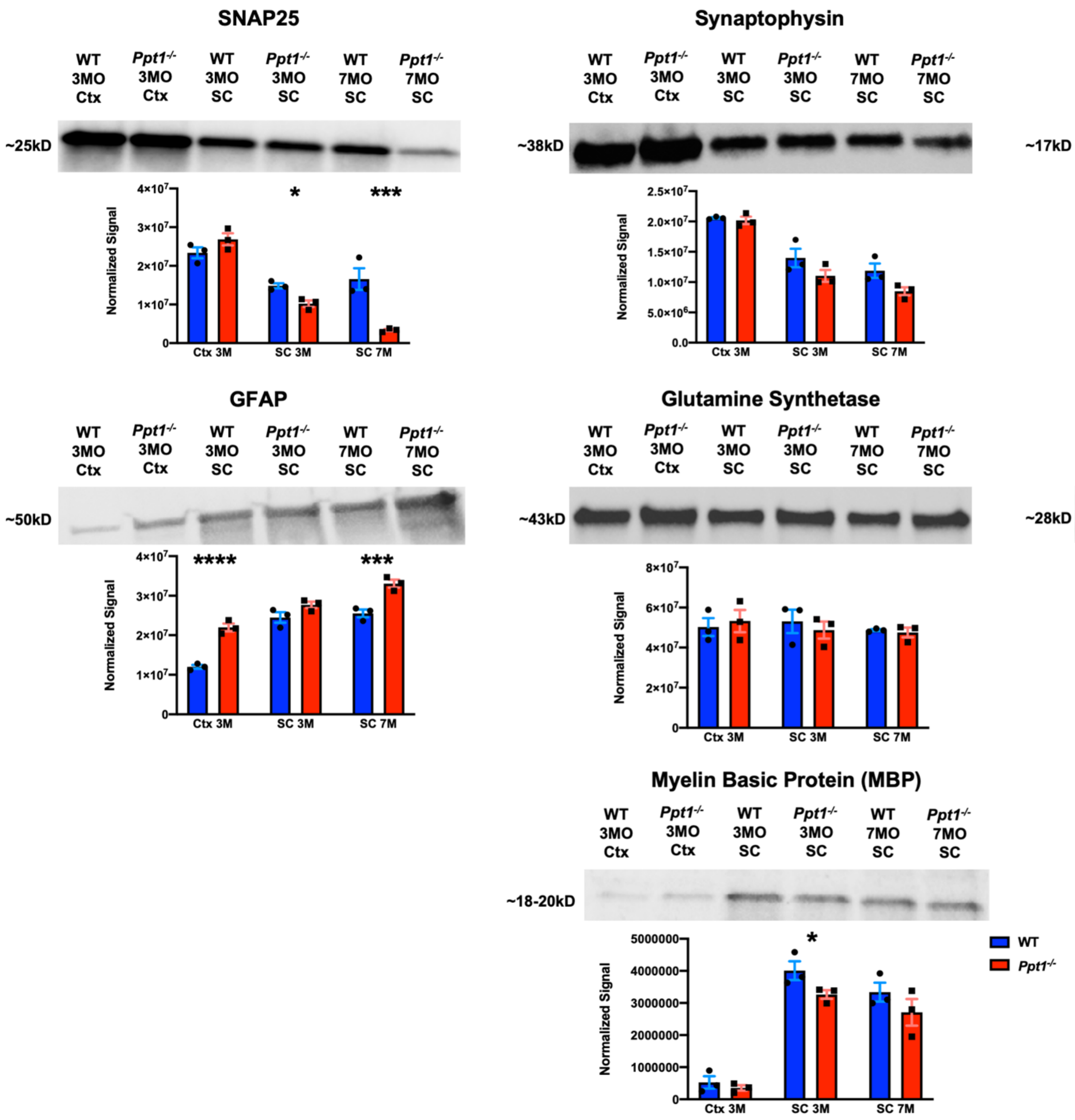

SNAP25
WT 3MO Ctx
Ppt1-/- 3MO Ctx
WT 3MO SC
Ppt1-/- 3MO SC
WT 7MO SC
Ppt1-/- 7MO SC
~25kD
Normalized Signal
Ctx 3M
SC 3M
SC 7M
*
***
Synaptophysin
~38kD
COX IV
~17kD
GFAP
~50kD
****
***
Glutamine Synthetase
~43kD
Calbindin
~28kD
Myelin Basic Protein (MBP)
~18-20kD
*
WT
Ppt1-/-

**SUPPLEMENTARY FIGURE 2 – Quantitative Fluorescent Western Blotting (QFWB) of proteins show similar trends to LC-MS/MS data.**

Representative bands and quantified statistical QFWB analysis of candidate proteins from cortical and spinal cord tissue homogenates predicted to be upregulated, downregulated or show no change between WT and Ppt1-/- tissue at 3 or 7 months respectively. Higher cortical signal for synaptic markers (SNAP25, Synaptophysin), Cytochrome Oxidase IV (COX IV), and Calbindin are due to higher cortical neuronal density, and higher spinal cord signal for MBP is due to greater proportion of white matter [15,32,38]. All probed proteins show similar trends to predicted LC-MS/MS ratios (Supplementary Table S1). All data were obtained from 3 blots with one lane per group. Only the 18-20kD band was analyzed for MBP, as per manufacturer's recommendations (EMD Millipore, MAB386). P-values - *$p \leq 0.05$, ***$p \leq 0.001$, ****$p \leq 0.0001$; multiple two-tailed, unpaired, parametric t-test with Bonferroni-Dunn correction Values shown are mean ± SEM. (n = 3 mice/group).

**SPINAL CORD 7MO *Ppt1*$^{-/-}$ vs. WT – 1.2 FOLD EXPRESSION CHANGE CANONICAL PATHWAYS**

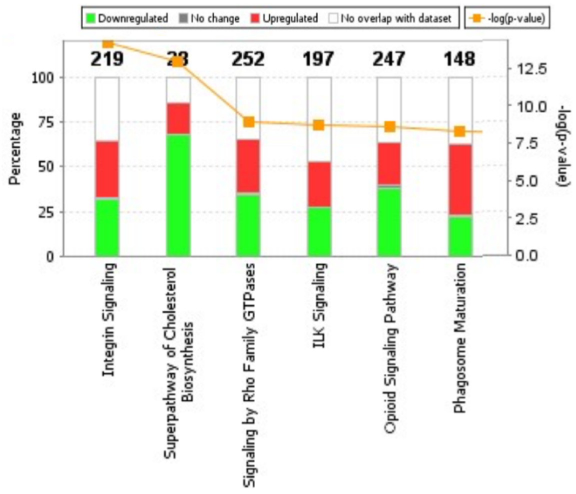


| Pathway | p-value |
|---|---|
| Integrin Signalling | 3.8E-11 |
| Cholesterol Biosynthesis | 3.21E-10 |
| Rho GTPase signalling | 2.83E-07 |
| ILK Signalling | 3.66E-07 |
| Opioid Signalling | 4.67 E-07 |

**SUPPLEMENTARY FIGURE 3 – Late Proteomic changes in the spinal cord of *Ppt1*$^{-/-}$ mice.**

Analysis of the differentially expressed proteins by 1.2 fold (20%) in the spinal cords of *Ppt1*$^{-/-}$ mice at 7 months of age, linked using Ingenuity Pathway Analysis (IPA) representing the top affected canonical pathways and their respective p-values.

# SPINAL CORD 3MO vs7MO - *Ppt1*$^{-/-}$ vs. WT

**A.** BIOLAYOUT 3D CLUSTERING ANALYSIS - AFFECTED NETWORKS

| Pathway | p-value | z-score |
|---|---|---|
| Organismal Death | 1.11E-12 | 3.644 |
| Organismal Mortality | 3.98E-12 | 3.565 |
| Dysfunction of neurons | 4.29E-05 | 2.164 |
| Benign Lesions | 0.000307 | 2.0 |
| Cytoplasm development decrease | 2.81E-12 | -3.095 |
| Cytoskeleton development decrease | 2.03E-09 | -2.85 |
| Formation of filaments | 2.02E-09 | -2.652 |
| Potentiation of synapse | 5.01E-08 | -2.521 |
| microtubule dynamics | 1.95E-14 | -2.124 |

**B.** BIOLAYOUT 3D CLUSTERING ANALYSIS – PROGRESSIVE OR SIMILAR CHANGES

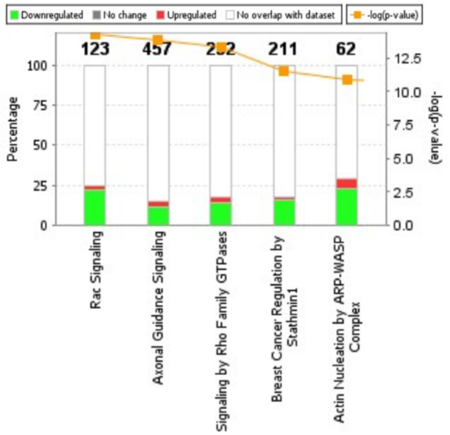


| Pathway | p-value |
|---|---|
| Rac Signaling | 7.09E-10 |
| Axonal Guidance Signaling | 1.19E-09 |
| Signaling by Rho Family GTPases | 2.91E-09 |
| Breast Cancer Regulation by Stathmin1 | 3.84E-08 |
| Actin Nucleation by ARP-WASP Complex 1 | 03E-07 |

**C.** BIOLAYOUT 3D CLUSTERING ANALYSIS – 7 MONTH SPINAL CORD CHANGES

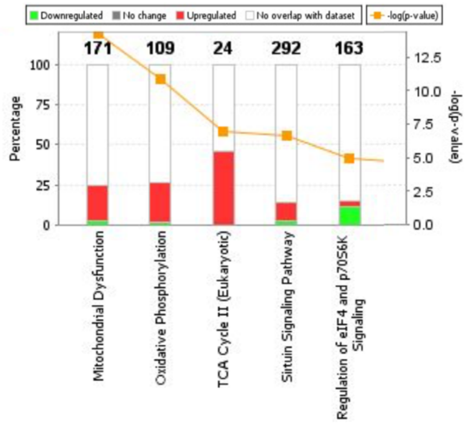


| Pathway | p-value |
|---|---|
| Integrin Signaling | 1.87E-17 |
| Signaling by Rho Family GTPases | 4.00E-12 |
| FAK Signaling | 1.26E-11 |
| RhoGDI Signaling | 1.91E-11 |
| Insulin Receptor Signaling | 2.00E-11 |

**SUPPLEMENTARY FIGURE 4 – *Biolayout express 3D* clustering analysis of changes in the spinal cord of *Ppt1*$^{-/-}$ mice at 3 and 7 months of age.**

Top affected cellular networks at 3 months of age in *Ppt1*$^{-/-}$ spinal cords (**A**) with their respective p-values and activation z-scores. Canonical pathways from the analysis of the differentially expressed protein clusters that show increased/similar changes (**B**) or greater change in expression at 7-months of age in *Ppt1*$^{-/-}$ spinal cords (**C**).

# CORTEX 3MO $Ppt1^{-/-}$ vs. WT

## CANONICAL PATHWAYS

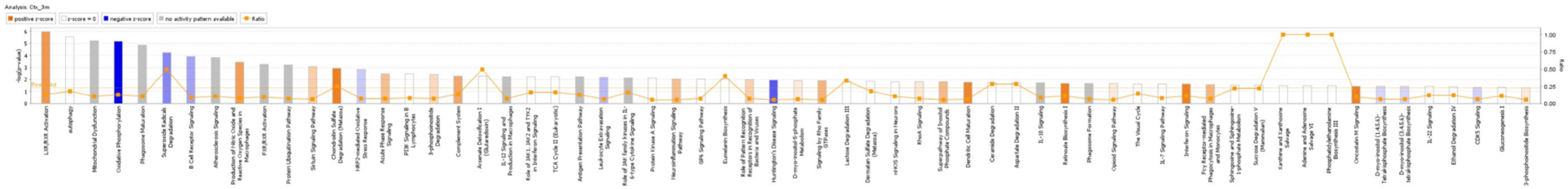

## AFFECTED NETWORKS

Cell Morphology, Cellular Assembly and Organization

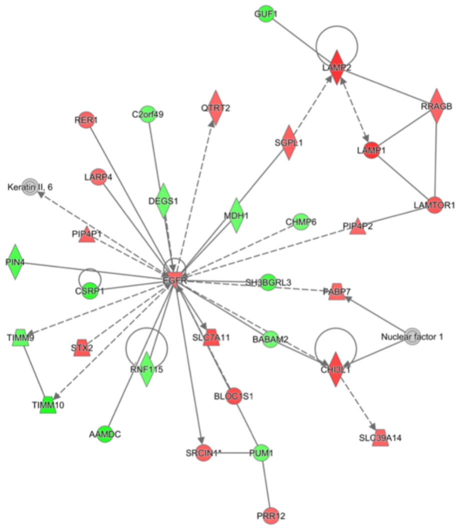


Metabolic Disease, Cellular Compromise, Embryonic Development

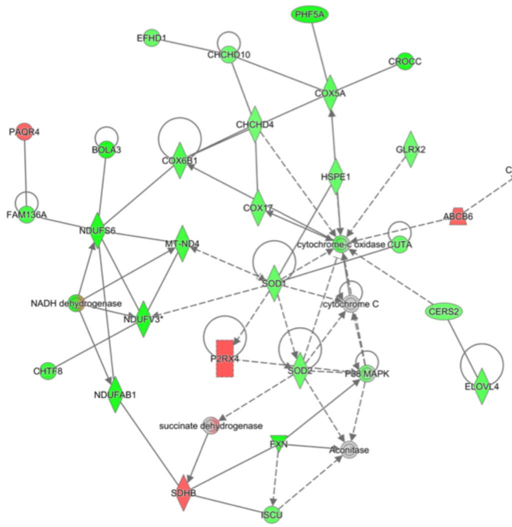


Energy Production, Molecular Transport, Nucleic Acid Metabolism

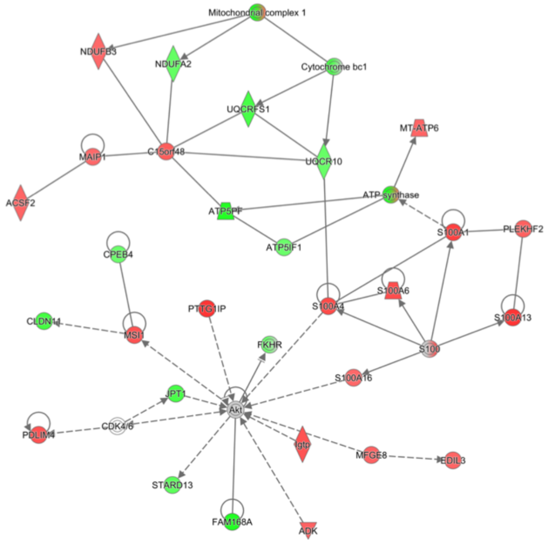

**SUPPLEMENTARY FIGURE 5- Early proteomic changes in the cortex of *Ppt1*$^{-/-}$ mice.** Analysis of the differentially expressed proteins by 1.2 fold (20%) in the cortex of *Ppt1*$^{-/-}$ mice at 3 months of age, linked using *Ingenuity Pathway Analysis* (*IPA*) representing the top affected canonical pathways and cellular networks including Cell Morphology, Cellular Assembly and Organization, Carbohydrate Metabolism (*IPA* score = 52), Metabolic Disease, Cellular Compromise, Embryonic Development (*IPA* score = 42), Energy Production, Molecular Transport, Nucleic Acid Metabolism (*IPA* score = 39).

**BIOLAYOUT 3D CLUSTERING ANALYSIS – SIMILAR CHANGES 3M – SC vs. CTX**

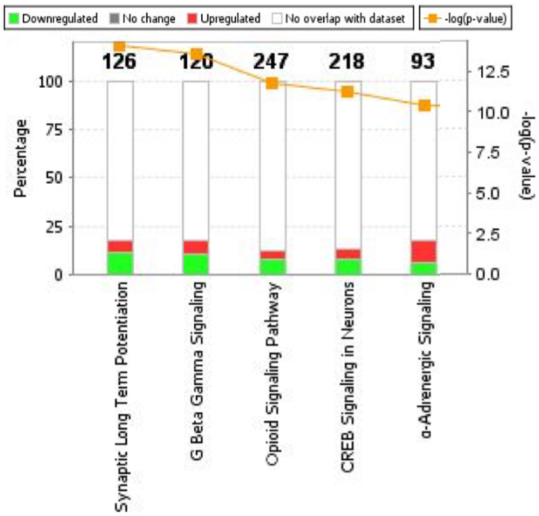


| Pathway | p-value |
|---|---|
| Synaptic Long Term Potentiation | 6.10E-07 |
| G Beta Gamma Signaling | 1.05E-06 |
| Opioid Signaling Pathway | 6.19E-06 |
| CREB Signaling in Neurons | 1.11E-05 |
| Adrenergic Signaling | 2.46E-05 |

**SUPPLEMENTARY FIGURE 6 –Similarly affected pathways in the cortex and spinal cord of 3-month-old *Ppt1*$^{-/-}$ mice.**

*Biolayout express 3D* clustering analysis reveals affected canonical pathways from the analysis of the differentially expressed protein clusters that show similar changes in the cortex and spinal cord of 3-month-old *Ppt1*$^{-/-}$ mice including Synaptic Long Term Potentiation (p=6.10E-07), G Beta Gamma Signalling (p=1.05E-06), Opioid, Signalling Pathway (p=6.19E-06), CREB Signalling in Neurons (p=1.11E-05) and adrenergic Signalling (p=2.46E-05).

# SUPPLEMENTARY TABLES

| **PROTEIN** (Validation Method) | ***Ppt1$^{-/-}$*/WT RATIO - LC-MS/MS** | | |
|---|---|---|---|
| | **3 MONTH SPINAL CORD Ppt1$^{-/-}$/WT RATIO** | **3 MONTH CORTEX Ppt1$^{-/-}$/WT RATIO** | **7 MONTH SPINAL CORD Ppt1$^{-/-}$/WT RATIO** |
| **CD68 / Macrosialin** (IHC) | 1.2397077 | 2 | 10.70342044 |
| **Glial Fibrillary Acidic Protein (GFAP)** (IHC & WB) | 1.125058485 | 2 | 1.484523571 |
| **Calbindin** (IHC & WB) | 1.079228237 | 0.952637998 | 0.920187651 |
| **Calretinin** (IHC) | 1.049716684 | 0.959264119 | 0.846745312 |
| **Synaptosomal-associated protein 25 (SNAP25)** (WB) | 0.986232704 | 1.148698355 | 0.933032992 |
| **Synaptophysin** (WB) | 0.965936329 | 1.035264924 | 0.876605721 |
| **Cytochrome c oxidase subunit 4 (COX IV)** (WB) | 1.086734863 | 0.946057647 | 0.926588062 |
| **Glutamine Synthetase** (WB) | 1.028113827 | 1.021012126 | 1.049716684 |
| **Myelin Basic Protein (MBP)** (WB) | 0.835087919 | 0.876605721 | 0.784584098 |

**SUPPLEMENTARY TABLE 1 - Relative LC-MS/MS expression ratios of proteins probed by immunohistochemistry (IHC) or Western Blotting (WB).**
Corresponding normalized abundance ratios between *Ppt1$^{-/-}$* vs. WT tissues, across all groups analysed for 9 proteins – showing similar trends between LC-MS/MS ratios and IHC and WB data (Figure 1, Supplementary Figures 1,2).

**SPINAL CORD 3M *Ppt1*$^{-/-}$ vs. WT 1.2-fold expression change**

**IPA ANALYSIS**

**<u>TOP 5 AFFECTED CANONICAL PATHWAYS</u>**

| INGENUITY CANONICAL PATHWAY | -log(p-value) | RATIO | MOLECULES |
|---|---|---|---|
| Phagosome Maturation | 6.1 | 0.0743 | B2M,CTSC,CTSD,CTSF,CTSH,CTSZ,HLA-A,LAMP1,LAMP2,TCIRG1,TUBA1A |
| autophagy | 5.27 | 0.113 | CTSC,CTSD,CTSF,CTSH,CTSZ,LAMP1,LAMP2 |
| Interferon Signaling | 4.35 | 0.139 | IFIT3,ISG15,PSMB8,STAT1,STAT2 |
| Antigen Presentation Pathway | 4.23 | 0.132 | B2M,HLA-A,PSMB8,PSMB9,TAPBP |
| Cytotoxic T Lymphocyte-mediated Apoptosis of Target Cells | 3.39 | 0.125 | B2M,DFFA,FCER1G,HLA-A |

**SUPPLEMENTARY TABLE 2 – SPINAL CORD 3M *Ppt1*$^{-/-}$ vs. WT 1.2-fold expression change IPA analysis top affected canonical pathways.**

Corresponding data for Figure 3. For full list see **Supplementary Excel File**.

## SPINAL CORD 3M *Ppt1*$^{-/-}$ vs. WT 1.2-fold expression change

## IPA ANALYSIS

## TOP 5 AFFECTED CELLULAR NETWORKS

| Network | Score | Focus Molecules | Molecules in Network |
|---|---|---|---|
| Humoral Immune Response, Inflammatory Response, Nutritional Disease | 52 | 28 | C1QA,Cathepsin,Cd59a,CROCC,CTSC,CTSD,CTSF,CTSH,CTSZ,DPH2,FABP5,FUCA1,FUCA2,HEXB,IgG,Igh (family),IGHG1,Ighg2b,Ighg2c,Igkv1-117,LAMP2,MHC II,MOG,Mt1,NFkB (complex),NKIRAS2,P-TEFb,PADI2,RALGAPA2,SATB1,SNX10,TCIRG1,TMEM106B,Vacuolar H+ ATPase,ZFAND5 |
| Cell Morphology, Cellular Assembly and Organization, Cellular Function and Maintenance | 42 | 24 | Alpha tubulin,calpain,CD3,CDK8,CLGN,CLN5,Cyclin E,DCLK1,DUSP22,EDIL3,EPHB6,FSH,GBP4,Histone h4,Hsp90,LAMP1,LAMTOR1,LAMTOR5,Mapk,MBP,MED23,NAAA,NAGLU,PLP1,POLD3,PTPRC,Serine Protease,SHANK2,SLC23A2,STAT1,TCR,Tgf beta,TPP1,TUBA1A,ZMYM2 |
| Cell-To-Cell Signaling and Interaction, Nervous System Development and Function, Cell Morphology | 35 | 21 | Ap1,BCAS1,Cg,CLDN11,Collagen type IV,Creb,DAPK2,GBP2,GBP7,GTPBP4,Keratin,KRT17,KRT24,KRT77,KRT78,KRT82,LGALS3BP,LYAR,Mek,MGST1,MYO6,Pdgf (complex),PDGF BB,PI3K (complex),PI3K (family),Ppp2c,PTPase,PTPN3,SCARB2,SCUBE1,SELENOF,Sos,THBS1,thyroid hormone receptor,Tpm1 |
| Molecular Transport, Lipid Metabolism, Small Molecule Biochemistry | 28 | 18 | A1BG,AMOT,AMPK,ASAH1,C5,c-Src,CERS2,DAB2IP,DHCR24,DSP,GRN,GTPase,HDL,hemoglobin,IL1,IL12 (complex),immune complex,Jnk,Krt2,MAC,mediator,NADPH oxidase,Nos,Nos1ap,P2RX4,PKP2,PRAG1,Pro-inflammatory Cytokine,PSAP,S100A1,S100A13,TCF,Tlr,TSH,UNC5B |
| Developmental Disorder, Hereditary Disorder, Immunological Disease | 26 | 17 | AIF1,Akt,Alpha 1 antitrypsin,Ap2,APOA2,C1q,C1QB,C1QC,CD63,CD68,chymotrypsin,COL4A2,Collagen(s),Complement,EEF2K,elastase,Fibrinogen,Gm-csf,GPIIB-IIIA,Igm,Igtp,Immunoglobulin,LDL,NPC1,PARM1,PPT1,PTGDS,PTTG1IP,SERPINA1,SERPINA3,SERPINA6,Sphk,Srebp,STAT5a/b,SYK/ZAP |

**SUPPLEMENTARY TABLE 3 – SPINAL CORD 3M *Ppt1*$^{-/-}$ vs. WT 1.2-fold expression change IPA analysis top 5 affected cellular networks.**

Corresponding data for Figure 3. For full list see **Supplementary Excel File**.

## SPINAL CORD 3M *Ppt1*$^{-/-}$ vs. WT 1.2-fold expression change

## IPA ANALYSIS

## AFFECTED DISEASE AND CELLULAR FUNCTION

| Diseases and Functions | Molecules in Network |
|---|---|
| Lysosomal storage disease | ASAH1,CLN5,CTSD,CTSF,FUCA1,GRN,HEXB,NAGLU,NPC1,NPC2,PLP1,PPT1,PSAP,TPP1 |
| Lipid Metabolism, Molecular Transport, Small Molecule Biochemistry | C5, CHRM1, GGT5, HEXB, IGHG1, LAMP1, LAMP2, LPAR1, NPC1, NPC2, NPY, PSAP, RENBP, SCARB2, YY1< ASAH1 |
| Immune Response | C5, CD68, CTSD, DDX58, FCER1G, GRN, HLA-A, IFIH1, IGHG, IL33, ISG15, IGHG2B, LYAR, NPY, PLP1, PRKCA, PSMB8, PTPRC, SPRY2, STAT1, TAPBP, TCIRG1, THBS1, VAV1, APOA2, C1QA |
| Glial Defects | MBP,MPZ,Mt1,Mt3,NCAM1,PLP1,PRX,PSAP,PTPRC,SCARB2,C5,MOG,PRKCA,PTGDS,STAT1,TNFRSF21,CERS2,GRN,LAMP1,PPT1,CTSF,B2M,CD59a,EPHB6,FCER1G,HLA-A,IGHG1,IGHG2b,IRGM1,NPY,STST2,CD9,CTSD,HEXB,KCNJ10,LPAR1 |
| Neurodegeneration and abnormal morphology of CNS | CHRM1,CLN5,DCLK1,DFFA,GRN,HEXB,KCNJ10,LRFN2,MARKSL,MBP,MT1,MT3,NAGLU,NCAM,PLP1,PPT1,PSAP,PTPRC,SHANK2,TPP1,CTSD,CTSF,LPAR1,MOG,MPZ,PRX,SCARB2,SERPINA3,SPRY2,CERS2,NPC1,NPC2,STAT1,B2M,C1QA,CCP110 |
| Movement disorders | B2M,BCAS1,C5,CD68,CHRM1,CTSD,CTSF,DNAJC6,HEXB,IGSF5,KCNH7,KCNJ10,LAMP1,LAMP2,MBP,MECR,MOG,MPZ,NCAM1,NPC1,NPC2,NPY,PDE4DIP,PLP1,PPM1B,PPT1,PRX,PSAP,PSMB8,PSMB9,PTPN3,SCARB2,SELENOF,SERPINA1,SERPINA3,SHANK2,TPP1,TUBA1A,TMSB4X,ADRA2A,ASAH1,ATP2B3 |
| Abnormal myelination | MPZ,PRX,PSAP,SCARB2,HEXB,KCNJ10,MBP,MOBP,PLP1,PTPRC,TNFRSF21,MOG,PTGDS,STS1,CD59A,TPP1,LPAR1 |
| Sensory system development | GRN,HEXB,MARCKSL1,MT1,NAGLU,NCAM1,PPT1,PSAP,PSMB10,PSMB8, RENBP,SELENOF,SPRY2,STAT1,THBS1,YY1,BCAR3,CTSD |

**SUPPLEMENTARY TABLE 4 – SPINAL CORD 3M *Ppt1*$^{-/-}$ vs. WT 1.2-fold expression change IPA analysis affected disease and cellular function.**

Corresponding data for Figure 4. For full list and individual p-values, z-score and predicted activation see **Supplementary Excel File**.

## SPINAL CORD 3M vs.7M *Ppt1*$^{-/-}$ vs. WT BIOLAYOUT 3D CLUSTERING

## IPA ANALYSIS of SC 3M CHANGES

## <u>TOP 5 AFFECTED CANONICAL PATHWAYS OVERALL</u>

| INGENUITY CANONICAL PATHWAY | -log(p-value) | RATIO | MOLECULES |
|---|---|---|---|
| Mitochondrial Dysfunction | 14.3 | 0.304 | ACO2,ATP5F1A,ATP5F1B,ATP5F1C,ATP5MG,ATP5PB,ATP5PD,BACE1,COX4I1,COX5A,COX6B1,Cox6c,CPT1C,DHODH,GPX4,GSR,HTRA2,LRRK2,MAOA,MAPK10,MAPK9,MT-CYB,MT-ND1,NDUFA1,NDUFA11,NDUFA12,NDUFA2,NDUFA3,NDUFA4,NDUFA5,NDUFA6,NDUFAF2,NDUFB10,NDUFB5,NDUFS1,NDUFS2,NDUFS7,NDUFS8,NDUFV1,NDUFV2,NDUFV3,PARK7,PRDX3,SNCA,SOD2,TRAK1,TXN2,UQCR10,UQCRB,UQCRC1,UQCRC2,UQCRQ |
| Protein Ubiquitination Pathway | 10.2 | 0.226 | AMFR,ANAPC1,ANAPC2,ANAPC4,ANAPC5,CBL,CDC23,CDC34,CUL1,DNAJB1,DNAJB11,DNAJB2,DNAJB4,DNAJC10,DNAJC15,DNAJC16,DNAJC17,HSPA9,HSPD1,HSPE1,NEDD4L,PSMA1,PSMA2,PSMA4,PSMA6,PSMB1,PSMB2,PSMB7,PSMC2,PSMC3,PSMC4,PSMD1,PSMD12,PSMD4,PSMD6,PSMD8,PSMD9,SUGT1,UBE2A,UBE2F,UBE2G2,UBE2H,UBE2K,UBE2N,UBE2R2,UBE4A,UBR1,USP12,USP14,USP15,USP20,USP22,USP25,USP30,USP33,USP34,USP39,USP47,USP9X,XIAP |
| Oxidative Phosphorylation | 9.9 | 0.312 | ATP5F1A,ATP5F1B,ATP5F1C,ATP5MG,ATP5PB,ATP5PD,COX4I1,COX5A,COX6B1,Cox6c,MT-CYB,MT-ND1,NDUFA1,NDUFA11,NDUFA12,NDUFA2,NDUFA3,NDUFA4,NDUFA5,NDUFA6,NDUFB10,NDUFB5,NDUFS1,NDUFS2,NDUFS7,NDUFS8,NDUFV1,NDUFV2,NDUFV3,UQCR10,UQCRB,UQCRC1,UQCRC2,UQCRQ |
| EIF2 Signaling | 8.65 | 0.225 | AKT1,EIF1AY,EIF2B2,EIF2B4,EIF2S2,EIF2S3,EIF3B,EIF3C,EIF3D,EIF3H,EIF3K,EIF3M,EIF4A3,EIF5,FGFR2,MAPK3,PABPC1,PAIP1,PIK3CA,PIK3R1,PPP1CB,RAP1B,RPL10,RPL13A,RPL14,RPL17,RPL18,RPL22,Rpl22l1,RPL23,RPL26,RPL27,RPL27A,RPL28,RPL3,RPL35A,RPL37A,RPL7,RPL7L1,RPL9,RPS11,RPS17,RPS19,RPS21,RPS23,RPS24,RPS29,RPS4Y1,SOS2,WARS1,XIAP |
| tRNA Charging | 6.85 | 0.41 | AARS1,CARS1,DARS2,GARS1,HARS1,HARS2,KARS1,LARS1,NARS1,RARS2,SARS1,SARS2,VARS1,VARS2,WARS1,WARS2 |

**SUPPLEMENTARY TABLE 5 – SPINAL CORD 3M *Ppt1*$^{-/-}$ vs. WT BIOLAYOUT 3D PROTEIN CLUSTERS IPA analysis top 5 affected canonical pathways in spinal cord at 3 months.**

Corresponding data for Figure 6A. For full list see **Supplementary Excel File**.

**SPINAL CORD 3M vs.7M *Ppt1*$^{-/-}$ vs. WT BIOLAYOUT 3D CLUSTERING IPA ANALYSIS OF INCREASED EXPRESSION CLUSTERS SC 3M**

**TOP 5 AFFECTED CANONICAL PATHWAYS**

| INGENUITY CANONICAL PATHWAY | -log(p-value) | RATIO | MOLECULES |
|---|---|---|---|
| Mitochondrial Dysfunction | 23.1 | 0.269 | ACO2,ATP5F1A,ATP5F1B,ATP5F1C,ATP5MG,ATP5PB,ATP5PD,COX4I1,COX5A,COX6B1,Cox6c,DHODH,GPX4,HTRA2,MAOA,MAPK10,MAPK9,MT-CYB,NDUFA1,NDUFA11,NDUFA12,NDUFA2,NDUFA3,NDUFA4,NDUFA5,NDUFA6,NDUFAF2,NDUFB10,NDUFB5,NDUFS1,NDUFS2,NDUFS7,NDUFS8,NDUFV1,NDUFV2,NDUFV3,PARK7,PRDX3,SNCA,SOD2,TRAK1,TXN2,UQCRB,UQCRC1,UQCRC2,UQCRQ |
| Oxidative Phosphorylation | 17.5 | 0.294 | ATP5F1A,ATP5F1B,ATP5F1C,ATP5MG,ATP5PB,ATP5PD,COX4I1,COX5A,COX6B1,Cox6c,MT-CYB,NDUFA1,NDUFA11,NDUFA12,NDUFA2,NDUFA3,NDUFA4,NDUFA5,NDUFA6,NDUFB10,NDUFB5,NDUFS1,NDUFS2,NDUFS7,NDUFS8,NDUFV1,NDUFV2,NDUFV3,UQCRB,UQCRC1,UQCRC2,UQCRQ |
| Sirtuin Signaling Pathway | 12.1 | 0.151 | AKT1,ATG3,ATG9A,ATP5F1A,ATP5F1B,ATP5F1C,ATP5PB,GLS,GLUD1,H1f4,HSF1,LDHB,MLYCD,MT-CYB,NDRG1,NDUFA1,NDUFA11,NDUFA12,NDUFA2,NDUFA3,NDUFA4,NDUFA5,NDUFA6,NDUFAF2,NDUFB10,NDUFB5,NDUFS1,NDUFS2,NDUFS7,NDUFS8,NDUFV1,NDUFV2,NDUFV3,PAM16,PGAM1,PPID,PPIF,SLC25A4,SOD2,TIMM17A,TIMM8A,TIMM8B,TUBA4A,UQCRC2 |
| TCA Cycle II (Eukaryotic) | 8.78 | 0.458 | ACO2,CS,DLD,DLST,FH,IDH3A,IDH3B,IDH3G,MDH2,OGDHL,SUCLA2 |
| Protein Ubiquitination Pathway | 6.62 | 0.121 | AMFR,ANAPC4,CDC34,CUL1,DNAJB1,DNAJC15,DNAJC17,HSPA9,HSPD1,HSPE1,PSMA1,PSMA4,PSMB1,PSMB7,PSMC3,PSMC4,PSMD6,PSMD8,PSMD9,SUGT1,UBE2A,UBE2F,UBE2H,UBE2K,UBE2N,UBE2R2,UBE4A,USP12,USP14,USP15,USP20,USP33 |

**SUPPLEMENTARY TABLE 6 – SPINAL CORD 3M *Ppt1*$^{-/-}$ vs. WT BIOLAYOUT 3D IPA analysis of increased expression clusters top 5 affected canonical pathways in spinal cord at 3 months.**

Corresponding data for Figure 6B. For full list see **Supplementary Excel File**.

## SPINAL CORD 3M vs.7M *Ppt1*$^{-/-}$ vs. WT BIOLAYOUT 3D CLUSTERING

## IPA ANALYSIS OF DECREASED EXPRESSION CLUSTERS SC 3M

## <u>TOP 5 AFFECTED CANONICAL PATHWAYS</u>

| INGENUITY CANONICAL PATHWAY | -log(p-value) | RATIO | MOLECULES |
|---|---|---|---|
| EIF2 Signaling | 9.87 | 0.163 | EIF2S2,EIF2S3,EIF3B,EIF3C,EIF3D,EIF3H,EIF3K,EIF3M,EIF4A3,FGFR2,MAPK3,PIK3CA,PIK3R1,PPP1CB,RPL10,RPL13A,RPL14,RPL17,RPL18,RPL22,Rpl22l1,RPL23,RPL27,RPL28,RPL3,RPL35A,RPL37A,RPL7,RPL7L1,RPL9,RPS17,RPS19,RPS21,RPS23,RPS24,SOS2,XIAP |
| Regulation of eIF4 and p70S6K Signaling | 5.81 | 0.147 | EIF2S2,EIF2S3,EIF3B,EIF3C,EIF3D,EIF3H,EIF3K,EIF3M,EIF4A3,FGFR2,MAPK3,MKNK1,PIK3CA,PIK3R1,PPM1L,PPP2R1B,PPP2R2A,PRKCZ,RPS17,RPS19,RPS21,RPS23,RPS24,SOS2 |
| Protein Kinase A Signaling | 5.07 | 0.102 | ACP1,ADCY2,ADCY5,AKAP1,AKAP5,ANAPC1,ANAPC2,ANAPC5,ATF2,CAMK2A,CDC16,CDC23,CDC27,DCC,GNAS,GNG12,H1-2,IKBKG,LIPE,MAPK3,MYH10,PDE10A,PDE4B,PDE7B,PHKB,PPP1CB,PPP1R1B,PRKCD,PRKCI,PRKCQ,PRKCZ,PTK2,PTPDC1,PTPN2,Ptprd,PTPRE,PTPRG,PTPRT,SMPDL3A,TCF4,YWHAE |
| Mitotic Roles of Polo-Like Kinase | 4.77 | 0.197 | ANAPC1,ANAPC2,ANAPC5,CDC16,CDC23,CDC27,PPM1L,PPP2R1B,PPP2R2A,RAD21,SMC1A,SMC3,STAG2 |
| mTOR Signaling | 4.04 | 0.116 | EIF3B,EIF3C,EIF3D,EIF3H,EIF3K,EIF3M,EIF4A3,FGFR2,MAPK3,PIK3CA,PIK3R1,PPM1L,PPP2R1B,PPP2R2A,PRKCD,PRKCI,PRKCQ,PRKCZ,RND2,RPS17,RPS19,RPS21,RPS23,RPS24 |

**SUPPLEMENTARY TABLE 7 – SPINAL CORD 3M *Ppt1*$^{-/-}$ vs. WT BIOLAYOUT 3D IPA analysis of decreased expression clusters top 5 affected canonical pathways in spinal cord at 3 months.**

Corresponding data for Figure 6C. For full list see **Supplementary Excel File**.

## SPINAL CORD vs. CTX 3M *Ppt1*$^{-/-}$ vs. WT BIOLAYOUT 3D CLUSTERING

## IPA ANALYSIS OF CORTEX CHANGES

## TOP 5 AFFECTED CANONICAL PATHWAYS

| INGENUITY CANONICAL PATHWAY | -log(p-value) | RATIO | MOLECULES |
|---|---|---|---|
| Caveolar-mediated Endocytosis Signaling | 10.9 | 0.423 | ABL1,ACTB,ACTC1,ARCN1,CAVIN1,COPA,COPB1,COPB2,COPE,COPG1,COPG2,DNM2,FLNA,FLNB,FLOT2,INSR,ITGA1,ITGA3,ITGA5,ITGA6,ITGA7,ITGAM,ITGAV,ITGB1,ITGB2,ITGB5,MAP3K2,PTPN1,RAB5C,SRC |
| Integrin Signaling | 10.1 | 0.265 | ABL1,ACTB,ACTC1,ACTN2,ACTN4,AKT2,ARF5,ARHGAP5,ARHGEF7,ARPC1B,ARPC3,ARPC5,BCAR3,CAPN2,CAPNS1,CRK,DOCK1,FGFR2,ILK,ITGA1,ITGA3,ITGA5,ITGA6,ITGA7,ITGAM,ITGAV,ITGB1,ITGB2,ITGB5,LIMS1,MAPK1,MPRIP,MRAS,MYL9,MYLK,NRAS,PAK2,PAK4,PAK6,PARVA,PARVB,PFN1,PIK3C2A,PIKFYVE,PPP1R12A,Ppp1r12b,PXN,RAP2B,RAPGEF1,RHOC,RND2,ROCK1,RRAS,RRAS2,SRC,TLN1,TSPAN6,VCL |
| mTOR Signaling | 9.15 | 0.261 | AKT1S1,AKT2,EIF3A,EIF3E,EIF3F,EIF3G,EIF3H,EIF3I,EIF3K,EIF3L,EIF4A1,EIF4G2,FGFR2,HMOX1,INSR,MAPK1,MAPKAP1,MLST8,MRAS,MTOR,NAPEPLD,NRAS,PIK3C2A,PLD2,PLD3,PPM1L,PPP2R1B,PPP2R5A,PRKAA2,PRKAG2,RAP2B,RHOC,RND2,RPS10,RPS14,RPS15A,RPS16,RPS17,RPS18,RPS19,RPS2,RPS20,RPS24,RPS25,RPS28,RPS3,RPS6KA3,RPS6KC1,RPS8,RPSA,RPTOR,RRAS,RRAS2,TSC2 |
| Regulation of eIF4 and p70S6K Signaling | 9.08 | 0.282 | AGO3,AKT2,EIF2A,EIF2S1,EIF2S2,EIF3A,EIF3E,EIF3F,EIF3G,EIF3H,EIF3I,EIF3K,EIF3L,EIF4A1,EIF4G2,FGFR2,ITGA3,ITGA5,ITGB1,MAPK1,MAPK14,MRAS,MTOR,NRAS,PIK3C2A,PPM1L,PPP2R1B,PPP2R5A,RAP2B,RPS10,RPS14,RPS15A,RPS16,RPS17,RPS18,RPS19,RPS2,RPS20,RPS24,RPS25,RPS28,RPS3,RPS8,RPSA,RRAS,RRAS2 |
| EIF2 Signaling | 8.49 | 0.247 | ACTB,ACTC1,AGO3,AKT2,EIF2A,EIF2AK4,EIF2S1,EIF2S2,EIF3A,EIF3E,EIF3F,EIF3G,EIF3H,EIF3I,EIF3K,EIF3L,EIF4A1,EIF4G2,EIF5B,FGFR2,HSPA5,IGF1R,INSR,MAPK1,MRAS,NRAS,PIK3C2A,Ppp1cc,PTBP1,RAP2B,RPL12,RPL17,RPL18A,RPL24,RPL27,RPL30,RPL5,RPL9,RPLP0,RPS10,RPS14,RPS15A,RPS16,RPS17,RPS18,RPS19,RPS2,RPS20,RPS24,RPS25,RPS28,RPS3,RPS8,RPSA,RRAS,RRAS2 |

**SUPPLEMENTARY TABLE 8 – SPINAL CORD vs. CTX 3M *Ppt1*$^{-/-}$ vs. WT BIOLAYOUT 3D CLUSTERING IPA analysis of top 5 affected canonical pathways in the cortex.** Corresponding data for Figure 7B. For full list see **Supplementary Excel File**.

**SPINAL CORD vs. CTX 3M *Ppt1*$^{-/-}$ vs. WT BIOLAYOUT 3D CLUSTERING**

**IPA ANALYSIS OF SPINAL CORD CHANGES**

**TOP 5 AFFECTED CANONICAL PATHWAYS**

| INGENUITY CANONICAL PATHWAY | -log(p-value) | RATIO | MOLECULES |
|---|---|---|---|
| Dopamine-DARPP32 Feedback in cAMP Signaling | 9.1 | 0.274 | ADCY3,ADCY5,ADCY8,CACNA1A,CACNA1C,CACNA1E,Calm1 (includes others),CAMKK2,CREM,CSNK1D,CSNK1E,CSNK1G2,GNAI2,GNAI3,GRIN2A,GRIN2B,GRIN2D,ITPR1,KCNJ10,KCNJ11,KCNJ16,KCNJ4,NOS1,PLCB4,PLCL1,PPM1J,PPP1CA,PPP1CB,PPP1R11,PPP1R3D,PPP1R7,PPP2CA,PPP2CB,PPP2R1A,PPP2R1B,PPP2R5B,PPP2R5D,PPP3CB,PRKAG2,PRKAR2B,PRKCA,PRKCB,PRKCD,PRKCE,PRKG2 |
| Breast Cancer Regulation by Stathmin1 | 7.69 | 0.237 | ADCY3,ADCY5,ADCY8,ARHGEF1,ARHGEF17,ARHGEF18,ARHGEF2,ARHGEF9,Calm1 (includes others),GAB1,GNA13,GNAI2,GNAI3,GNB1,GNB4,GNG11,GNG5,GRB2,ITPR1,MAP2K1,MAPK3,PIK3C2B,PIK3CA,PIK3CB,PIK3R2,PLCB4,PPM1J,PPP1CA,PPP1CB,PPP1R11,PPP1R3D,PPP1R7,PPP2CA,PPP2CB,PPP2R1A,PPP2R1B,PPP2R5B,PPP2R5D,PRKAG2,PRKAR2B,PRKCA,PRKCB,PRKCD,PRKCE,RALA,RALB,RAP2B,RRAS2,TUBA1A,TUBB3 |
| CREB Signaling in Neurons | 7.63 | 0.234 | ADCY3,ADCY5,ADCY8,AKT1,CACNA1A,CACNA1C,CACNA1E,CACNA1I,CACNB2,CACNB3,CACNG8,Calm1 (includes others),GAB1,GNA11,GNA13,GNAI2,GNAI3,GNB1,GNB4,GNG11,GNG5,GRB2,GRIK3,GRIK5,GRIN2A,GRIN2B,GRIN2D,GRM4,GRM5,ITPR1,MAP2K1,MAPK3,PIK3C2B,PIK3CA,PIK3CB,PIK3R2,PLCB4,PLCL1,POLR2B,POLR2E,PRKAG2,PRKAR2B,PRKCA,PRKCB,PRKCD,PRKCE,RALA,RALB,RAP2B,RPS6KA1,RRAS2 |
| CDK5 Signaling | 7.28 | 0.295 | ADCY3,ADCY5,ADCY8,CACNA1A,CAPN1,CDK5R1,LAMB1,MAP2K1,MAPK11,MAPK3,MAPK8,MAPK9,MAPT,PPM1J,PPP1CA,PPP1CB,PPP1R11,PPP1R3D,PPP1R7,PPP2CA,PPP2CB,PPP2R1A,PPP2R1B,PPP2R5B,PPP2R5D,PRKAG2,PRKAR2B,RALA,RALB,RAP2B,RRAS2 |
| GNRH Signaling | 7.03 | 0.246 | ADCY3,ADCY5,ADCY8,CACNA1A,CACNA1C,CACNA1E,CACNA1I,CACNB2,CACNB3,CACNG8,Calm1 (includes others),GNA11,GNAI2,GNAI3,GNB1,GNG11,GNG5,GRB2,ITPR1,MAP2K1,MAP2K3,MAP3K4,MAP3K5,MAP3K9,MAPK11,MAPK3,MAPK8,MAPK9,PAK6,PLCB4,PRKAG2,PRKAR2B,PRKCA,PRKCB,PRKCD,PRKCE,PTK2,PTK2B,RALA,RALB,RAP2B,RRAS2 |

**SUPPLEMENTARY TABLE 9 – SPINAL CORD vs. CTX 3M *Ppt1*$^{-/-}$ vs. WT BIOLAYOUT 3D CLUSTERING IPA analysis of top 5 affected canonical pathways in the cord.** Corresponding data for Figure 7C. For full list see **Supplementary Excel File**.

**SPINAL CORD 7M *Ppt1*$^{-/-}$ vs. WT 1.2-fold expression change**

**IPA ANALYSIS**

**<u>TOP 5 AFFECTED CANONICAL PATHWAYS</u>**

| INGENUITY CANONICAL PATHWAY | -log(p-value) | RATIO | MOLECULES |
|---|---|---|---|
| Integrin Signaling | 10.4 | 0.196 | ABL1,ACTN4,AKT2,AKT3,ARF3,ARPC1B,BCAR3,CAPN2,CAPNS1,DOCK1,GIT1,ILK,ITGA3,ITGA5,ITGA6,ITGA7,ITGAM,ITGB1,ITGB2,ITGB5,LIMS1,MAP2K4,MYLK,PAK1,PAK4,PARVA,PARVB,PFN2,PXN,RAC3,RAP1A,RHOB,RHOC,RHOF,RHOG,RRAS,RRAS2,TLN1,TNK2,TSPAN2,TSPAN6,TTN,VCL |
| Superpathway of Cholesterol Biosynthesis | 9.49 | 0.5 | ACAT2,CYP51A1,DHCR24,DHCR7,FDPS,HADHA,HADHB,HMGCS1,IDI1,MSMO1,MVD,MVK,NSDHL,TM7SF2 |
| Signaling by Rho Family GTPases | 6.55 | 0.155 | ARHGEF4,ARPC1B,CDC42EP2,CDH12,CDH2,CDH6,CDH8,CIT,CYFIP1,DES,EZR,GFAP,GNAI1,GNAL,GNAO1,GNB4,GNG2,GNG3,GNG4,IQGAP1,ITGA3,ITGA5,ITGB1,MAP2K4,MAP3K12,MSN,MYLK,NCF2,NFKB2,PAK1,PAK4,RELA,RHOB,RHOC,RHOF,RHOG,SLC9A1,STMN1,VIM |
| ILK Signaling | 6.44 | 0.168 | ACTN4,AKT2,AKT3,CASP3,DOCK1,DSP,FLNA,FLNB,FN1,GSK3A,ILK,ITGB1,ITGB2,ITGB5,LIMS1,MAP2K4,MAP2K6,MYH9,NFKB2,PARVA,PARVB,PPP2CA,PPP2R2C,PPP2R5A,PXN,RELA,RHOB,RHOC,RHOF,RHOG,RSU1,VCL,VIM |
| Opioid Signaling Pathway | 6.33 | 0.154 | ADCY6,AKT2,AKT3,ARRB2,CACNA1A,CACNA2D2,CACNB4,CACNG2,CAMK1D,GNAI1,GNAL,GNAO1,GNG2,GRIN3A,ITPR2,KCNJ9,LYN,MAP2K4,MAP2K6,MAPK4,NFKB2,OPRM1,PDE1B,PENK,PLD2,PNOC,PRKCB,PRKCE,RAC3,RAP1A,RGS12,RGS7,RPS6KA2,RRAS,RRAS2,RYR3,SCN7A,SLC12A5 |

**SUPPLEMENTARY TABLE 10 – SPINAL CORD 7M *Ppt1*$^{-/-}$ vs. WT 1.2-fold expression change IPA analysis top 5 affected canonical pathways.**

Corresponding data for Supplementary Figure 3. For full list see **Supplementary Excel**

**SPINAL CORD 3M vs.7M *Ppt1*$^{-/-}$ vs. WT BIOLAYOUT 3D CLUSTERING**

**IPA ANALYSIS of SC PROGRESSIVE OR SIMILAR CHANGES**

**TOP 5 AFFECTED CANONICAL PATHWAYS OVERALL**

| INGENUITY CANONICAL PATHWAY | -log(p-value) | RATIO | MOLECULES |
|---|---|---|---|
| Rac Signaling | 9.15 | 0.244 | ABI2,ACTR2,ACTR3,ARFIP2,ARPC2,BAIAP2,CDK5R1,CYFIP2,GRB2,HRAS,KRAS,MAP2K1,MAP2K7,MCF2L,PAK5,PIK3C2B,PIK3C3,PIP4K2A,PIP4K2B,PIP4K2C,PIP5K1A,PIP5K1C,PLD1,RAF1,RALB,RAP1A,RAP2A,SH3RF1,TIAM1,WASF1 |
| Axonal Guidance Signaling | 8.92 | 0.149 | ABLIM3,ACTR2,ACTR3,ADAM11,ARPC2,BAIAP2,DPYSL2,ECEL1,EIF4E,EPHA5,EPHA7,GIT1,GNA11,GNA12,GNA13,GNAI1,GNAZ,GNB4,GNB5,GNG11,GNG13,GRB2,HRAS,ITSN1,KRAS,MAG,MAP2K1,MYL12A,MYL4,MYL6,NCK1,NGEF,NTNG1,NTNG2,NTRK2,NTRK3,PAK5,PIK3C2B,PIK3C3,PITRM1,PLCB1,PLCL1,PLXNA2,PPP3CB,PRKAR1A,PRKAR2B,PRKCB,PRKCE,PSMD14,RAC3,RAF1,RALB,RAP1A,RAP2A,RTN4,RTN4R,SDCBP,SEMA4B,SEMA4F,SHANK2,SLIT1,SRGAP2,SRGAP3,TUBA8,TUBB1,TUBB6,UNC5A,VASP |
| Signaling by Rho Family GTPases | 8.54 | 0.179 | ACTR2,ACTR3,ARFIP2,ARHGEF18,ARHGEF9,ARPC2,BAIAP2,CDC42EP4,CDH6,CDH8,CDH9,GNA11,GNA12,GNA13,GNAI1,GNAZ,GNB4,GNB5,GNG11,GNG13,GRB2,MAP2K1,MAP2K7,MAP3K12,MYL12A,MYL4,MYL6,PAK5,PIK3C2B,PIK3C3,PIP4K2A,PIP4K2B,PIP4K2C,PIP5K1A,PIP5K1C,PLD1,RAF1,RHOB,RHOG,RHOT1,RHOT2,SEPTIN3,SEPTIN6,STMN1,WASF1 |
| Breast Cancer Regulation by Stathmin1 | 7.42 | 0.18 | ADCY3,ADCY8,ARHGEF18,ARHGEF9,GNA13,GNAI1,GNB4,GNB5,GNG11,GNG13,GRB2,HRAS,KRAS,MAP2K1,PIK3C2B,PIK3C3,PLCB1,PPM1J,PPP1R11,PPP1R14A,PPP1R3D,PPP2CA,PPP2R1A,PPP2R2C,PPP2R5B,PRKAR1A,PRKAR2B,PRKCB,PRKCE,RAF1,RALB,RAP1A,RAP2A,RB1CC1,STMN1,TUBA8,TUBB1,TUBB6 |
| Actin Nucleation by ARP-WASP Complex | 6.99 | 0.29 | ACTR2,ACTR3,ARPC2,BAIAP2,GNA12,GRB2,HRAS,KRAS,NCK1,RALB,RAP1A,RAP2A,RHOB,RHOG,RHOT1,RHOT2,VASP,WASF1 |

**SUPPLEMENTARY TABLE 11 – SPINAL CORD 3M vs. 7M *Ppt1*$^{-/-}$ vs. WT BIOLAYOUT 3D PROTEIN CLUSTERS IPA analysis top 5 similarly or progressively affected canonical pathways in spinal cord between 3&7 months.**

Corresponding data for Supplementary Figure 4B. For full list see **Supplementary Excel File**.

## SPINAL CORD 3M vs.7M *Ppt1*$^{-/-}$ vs. WT BIOLAYOUT 3D CLUSTERING

## IPA ANALYSIS of SC 7M CHANGES

## TOP 5 AFFECTED CANONICAL PATHWAYS OVERALL

| INGENUITY CANONICAL PATHWAY | -log(p-value) | RATIO | MOLECULES |
|---|---|---|---|
| Integrin Signaling | 16.7 | 0.37 | ABL1,ACTB,ACTC1,ACTN4,AKT2,AKT3,Arf2,ARF5,ARHGAP26,ARHGAP5,ARHGEF7,ARPC1B,ARPC3,ARPC5,BCAR3,CAPN1,CAPN2,CAPN7,CAPNS1,CRK,CTTN,DOCK1,FGFR3,FNBP1,FYN,GSK3B,ILK,IRS2,ITGA1,ITGA3,ITGA5,ITGA6,ITGA7,ITGAM,ITGB1,ITGB2,ITGB5,LIMS1,MAP2K2,MAP2K4,MAPK1,MAPK8,MPRIP,MRAS,MYL9,MYLK,NRAS,PAK1,PAK2,PAK4,PAK6,PARVA,PARVB,PFN1,PFN2,PIK3C2A,PIK3CB,PIK3R2,PIK3R4,PIKFYVE,PPP1R12A,Ppp1r12b,PTEN,PXN,RALA,RAP2B,RAPGEF1,RHOA,RHOC,RHOF,ROCK1,RRAS,RRAS2,SOS1,SRC,TLN1,TLN2,TSPAN6,TTN,VCL,Wasl |
| Signaling by Rho Family GTPases | 11.4 | 0.31 | ACTB,ACTC1,ARHGEF10,ARHGEF12,ARHGEF4,ARHGEF7,ARPC1B,ARPC3,ARPC5,CDC42EP2,CDH10,CDH11,CDH18,CDH2,CDH20,CIT,CLIP1,CYFIP1,DES,EZR,FGFR3,FNBP1,GFAP,GNAI2,GNAI3,GNAL,GNAO1,GNAQ,GNG10,GNG2,GNG3,GNG4,GNG5,IQGAP1,IRS2,ITGA3,ITGA5,ITGB1,LIMK1,MAP2K2,MAP2K4,MAP3K9,MAPK1,MAPK8,MRAS,MSN,MYL6B,MYL9,MYLK,NCF2,NFKB1,NFKB2,PAK1,PAK2,PAK4,PAK6,PARD3,PI4KA,PIK3C2A,PIK3CB,PIK3R2,PIK3R4,PIKFYVE,PKN1,PPP1R12A,RACK1,RDX,RELA,RHOA,RHOC,RHOF,ROCK1,ROCK2,SEPTIN10,SEPTIN5,SEPTIN9,SLC9A1,VIM |
| FAK Signaling | 10.9 | 0.41 | ACTB,ACTC1,AKT2,AKT3,ARHGAP26,ARHGEF7,CAPN1,CAPN2,CAPN7,CAPNS1,CRK,DOCK1,FGFR3,FYN,GIT2,IRS2,ITGA3,ITGA5,ITGB1,MAP2K2,MAPK1,MRAS,NRAS,PAK1,PAK2,PAK4,PAK6,PIK3C2A,PIK3CB,PIK3R2,PIK3R4,PTEN,PXN,RALA,RAP2B,RRAS,RRAS2,SOS1,SRC,TLN1,TLN2,TNS1,VCL |
| RhoGDI Signaling | 10.7 | 0.339 | ACTB,ACTC1,ARHGAP1,ARHGAP12,ARHGAP35,ARHGAP5,ARHGDIA,ARHGDIB,ARHGEF10,ARHGEF12,ARHGEF4,ARHGEF7,ARPC1B,ARPC3,ARPC5,CD44,CDH10,CDH11,CDH18,CDH2,CDH20,CREBBP,EZR,FNBP1,GDI2,GNAI2,GNAI3,GNAL,GNAO1,GNAQ,GNG10,GNG2,GNG3,GNG4,GNG5,GRIP1,ITGA3,ITGA5,ITGB1,LIMK1,MRAS,MSN,MYL6B,MYL9,PAK1,PAK2,PAK4,PAK6,PI4KA,PIKFYVE,PPP1R12A,RACK1,RDX,RHOA,RHOC,RHOF,ROCK1,ROCK2,SRC,WASF2 |
| Insulin Receptor Signaling | 10.7 | 0.361 | AKT2,AKT3,ASIC1,BAD,CRK,EIF2B3,EIF2B5,FGFR3,FOXO1,FYN,GRB10,GSK3A,GSK3B,GYS1,INPP5D,INPPL1,INSR,IRS2,JAK2,MAP2K2,MAPK1,MAPK8,MRAS,MTOR,NRAS,PIK3C2A,PIK3CB,PIK3R2,PIK3R4,Ppp1cc,PPP1R10,PPP1R12A,PPP1R7,PRKACB,PRKAG1,PRKAG2,PRKAR2A,PTEN,PTPN1,PTPRF,RALA,RAP2B,RAPGEF1,RPS6KB1,RPTOR,RRAS,RRAS2,SOS1,STX4,SYNJ1,TRIP10,TSC1,TSC2 |

**SUPPLEMENTARY TABLE 12 – SPINAL CORD 3M *Ppt1*$^{-/-}$ vs. WT BIOLAYOUT 3D PROTEIN CLUSTERS IPA analysis top 5 affected canonical pathways in spinal cord at 7 months.**

Corresponding data for Supplementary Figure 4C. For full list see **Supplementary Excel File**.

**CORTEX 3M *Ppt1*$^{-/-}$ vs. WT 1.2-fold expression change**

**IPA ANALYSIS**

**<u>TOP 5 AFFECTED CANONICAL PATHWAYS</u>**

| INGENUITY CANONICAL PATHWAY | -log(p-value) | RATIO | MOLECULES |
|---|---|---|---|
| LXR/RXR Activation | 5.98 | 0.132 | A1BG,ABCA1,APOA1,APOE,C4A/C4B,CLU,FDFT1,HPX,IL33,LYZ,NCOR1,NFKB2,PON1,PON3,SERPINA1,VTN |
| autophagy | 5.56 | 0.177 | CTSA,CTSB,CTSC,CTSD,CTSF,CTSH,CTSO,CTSS,CTSZ,LAMP1,LAMP2 |
| Mitochondrial Dysfunction | 5.2 | 0.105 | ATP5PF,COX17,COX5A,COX6B1,GLRX2,MT-ATP6,MT-ND4,NDUFA2,NDUFAB1,NDUFB3,NDUFS6,NDUFV3,SDHB,SNCA,SOD2,TXN2,UQCR10,UQCRFS1 |
| Oxidative Phosphorylation | 5.16 | 0.128 | ATP5PF,COX17,COX5A,COX6B1,MT-ATP6,MT-ND4,NDUFA2,NDUFAB1,NDUFB3,NDUFS6,NDUFV3,SDHB,UQCR10,UQCRFS1 |
| Phagosome Maturation | 4.84 | 0.108 | CTSA,CTSB,CTSC,CTSD,CTSF,CTSH,CTSO,CTSS,CTSZ,DYNLL1,HLA-A,LAMP1,LAMP2,NCF2,TCIRG1,TUBA8 |

**SUPPLEMENTARY TABLE 13 – SPINAL CORD 3M *Ppt1*$^{-/-}$ vs. WT 1.2-fold expression change IPA analysis top affected canonical pathways.**

Corresponding data for Supplementary Figure 5. For full list see **Supplementary Excel File**.

## CORTEX 3M *Ppt1*$^{-/-}$ vs. WT 1.2-fold expression change

## IPA ANALYSIS

## TOP 5 AFFECTED CELLULAR NETWORKS

| Network | Score | Focus Molecules | Molecules in Network |
|---|---|---|---|
| Cell Morphology, Cellular Assembly and Organization, Carbohydrate Metabolism | 52 | 33 | AAMDC,BABAM2,BLOC1S1,C2orf49,CHI3L1,CHMP6,CSRP1,DEGS1,EGFR,FABP7,GUF1,Keratin II, 6,LAMP1,LAMP2,LAMTOR1,LARP4,MDH1,Nuclear factor 1,PIN4,PIP4P1,PIP4P2,PRR12,PUM1,QTRT2,RER1,RNF115,RRAGB,SGPL1,SH3BGRL3,SLC39A14,SLC7A11,SRCIN1,STX2,TIMM9,TIMM10 |
| Metabolic Disease, Cellular Compromise, Embryonic Development | 42 | 29 | ABCB6,Aconitase,BOLA3,CERS2,CHCHD4,CHCHD10,CHTF8,COX17,COX5A,COX6B1,CROCC,CUTA,CYP20A1,cytochrome C,cytochrome-c oxidase,EFHD1,ELOVL4,FAM136A,FXN,GLRX2,HSPE1,ISCU,MT-ND4,NADH dehydrogenase,NDUFAB1,NDUFS6,NDUFV3,P2RX4,P38 MAPK,PAQR4,PHF5A,SDHB,SOD1,SOD2,succinate dehydrogenase |
| Energy Production, Molecular Transport, Nucleic Acid Metabolism | 39 | 28 | ACSF2,ADK,Akt,ATP synthase,ATP5IF1,ATP5PF,C15orf48,CDK4/6,CLDN11,CPEB4,Cytochrome bc1,EDIL3,FAM168A,FKHR,Igtp,JPT1,MAIP1,MFGE8,Mitochondrial complex 1,MSI1,MT-ATP6,NDUFA2,NDUFB3,PDLIM4,PLEKHF2,PTTG1IP,S100,S100A1,S100A4,S100A6,S100A13,S100A16,STARD13,UQCR10,UQCRFS1 |
| Cardiovascular System Development and Function, Embryonic Development, Organ Development | 39 | 28 | 14-3-3,ACAT2,ADD1,APC/APC2,ATP1B1,CALU,CEP97,CEP104,DERL1,EPB41L3,FAM107B,FCGRT,FXYD7,GADD45,Gsk3,HNRNPA0,Importin alpha,JPT2,Macf1,MAPRE1,MCC,MDH2,MLC1,MYH10,Nfat (family),PDCD5,Snrpa (includes others),SNX3,Spectrin,TAGLN2,TMA7,TSC22D2,TTN,U2AF2,VIRMA |
| Cellular Development, Post-Translational Modification, Hair and Skin Development and Function | 39 | 28 | 26s Proteasome,AREL1,CD3,CLCN7,HOMER3,IKK (complex),Interferon alpha,LSM6,MITF,NAGA,NCBP2,p85 (pik3r),PHLDA3,PLEKHM2,PPM1B,PRKCB,PRPF18,snRNP,SNRPF,STARD3NL,STOM,STX3,SUMO1,SUMO2,SUMO3,TMPO,TXNL1,TXNL4A,UBE2,UBE2A,UBE2D2,UBE2L3,UBE2V1,UBQLN2,UQCC3 |

**SUPPLEMENTARY TABLE 14 – CORTEX 3M *Ppt1*$^{-/-}$ vs. WT 1.2-fold expression change IPA analysis top 5 affected cellular networks.**

Corresponding data for Supplementary Figure 5. For full list see **Supplementary Excel File**.

## SPINAL CORD vs. CTX 3M *Ppt1*$^{-/-}$ vs. WT BIOLAYOUT 3D CLUSTERING

## IPA ANALYSIS of SIMILAR CHANGES

## TOP 5 AFFECTED CANONICAL PATHWAYS OVERALL

| INGENUITY CANONICAL PATHWAY | -log(p-value) | RATIO | MOLECULES |
|---|---|---|---|
| Synaptic Long Term Potentiation | 6.21 | 0.175 | ADCY1,CACNA1C,GNA11,GRIA2,GRM1,GRM4,KRAS,PLCB1,PLCG1,PPP1CA,PPP1R1A,PPP1R3D,PPP3CB,PPP3CC,PPP3R1,PRKACA,PRKAR1A,PRKAR1B,PRKCA,PRKCG,RAF1,RAP1B |
| G Beta Gamma Signaling | 5.98 | 0.175 | ADCY1,CACNA1B,CACNA1C,CACNB1,CACNG8,GNA11,GNAZ,GNB1,GNB2,GNG7,KCNJ3,KRAS,PDPK1,PLCG1,PRKACA,PRKAR1A,PRKAR1B,PRKCA,PRKCG,RAF1,RAP1B |
| Opioid Signaling Pathway | 5.21 | 0.126 | ADCY1,ADCY3,BRAF,CACNA1B,CACNA1C,CACNB1,CACNG8,CDKN1B,GNB1,GNG7,KCNJ3,KRAS,MAP2K7,PDE1A,PDE1C,PLCB1,PPP3CB,PPP3CC,PPP3R1,PRKACA,PRKAR1A,PRKAR1B,PRKCA,PRKCG,RAF1,RAP1B,RGS3,RGS8,RPS6KA4,RPS6KA5,SCN7A |
| CREB Signaling in Neurons | 4.95 | 0.128 | ADCY1,ADCY3,CACNA1B,CACNA1C,CACNB1,CACNG8,GAB1,GNA11,GNAZ,GNB1,GNB2,GNG7,GRIA2,GRID2,GRIK2,GRIK5,GRM1,GRM4,KRAS,PLCB1,PLCG1,PRKACA,PRKAR1A,PRKAR1B,PRKCA,PRKCG,RAF1,RAP1B |
| α-Adrenergic Signaling | 4.61 | 0.172 | ADCY1,ADCY3,ADRA2A,GNB1,GNB2,GNG7,KRAS,PLCG1,PRKACA,PRKAR1A,PRKAR1B,PRKCA,PRKCG,PYGB,RAF1,RAP1B |

**SUPPLEMENTARY TABLE 15 – SPINAL CORD vs. CTX 3M *Ppt1*$^{-/-}$ vs. WT BIOLAYOUT 3D PROTEIN CLUSTERS IPA analysis top 5 similarly or progressively affected canonical pathways between spinal cord and cortex at 3 months.** Corresponding data for Supplementary Figure 6. For full list see **Supplementary Excel File**.

**Unedited Figures for Main Figure 4 - Disease and Cellular Function**

**LYSOSOMAL DEFECTS**

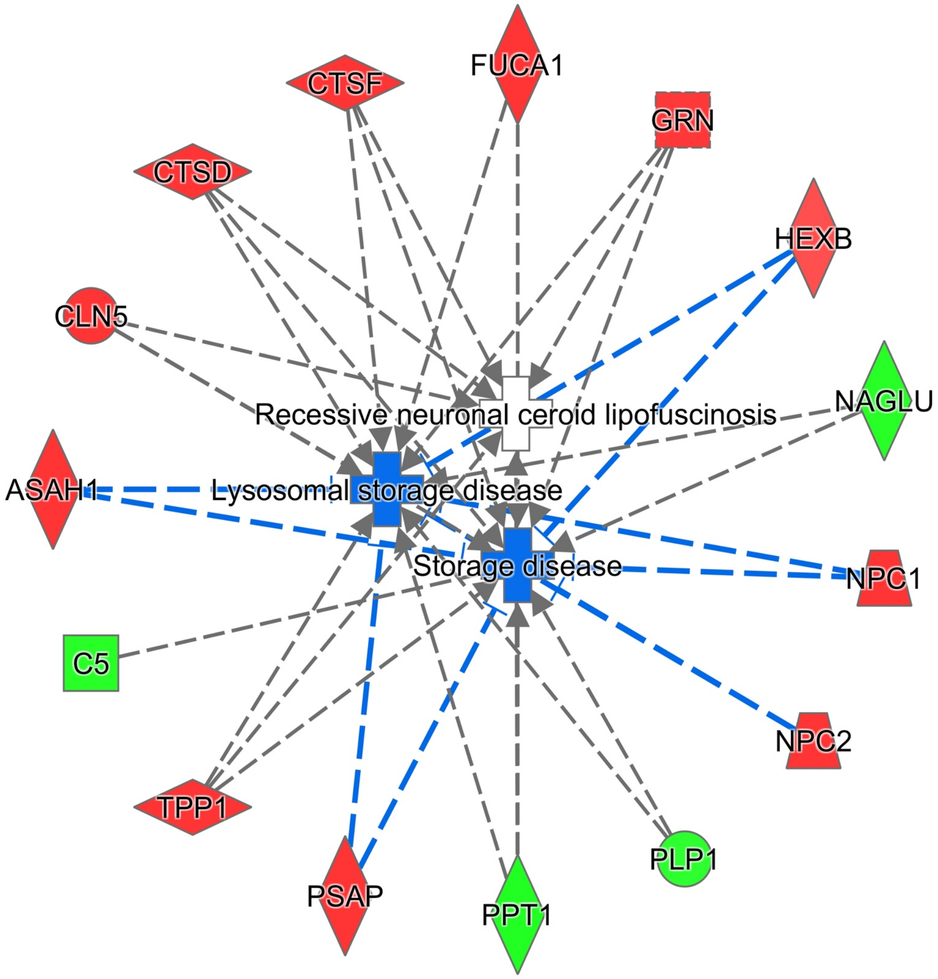

**Unedited Figures for Main Figure 4 - Disease and Cellular Function**

**LIPID METABOLISM**

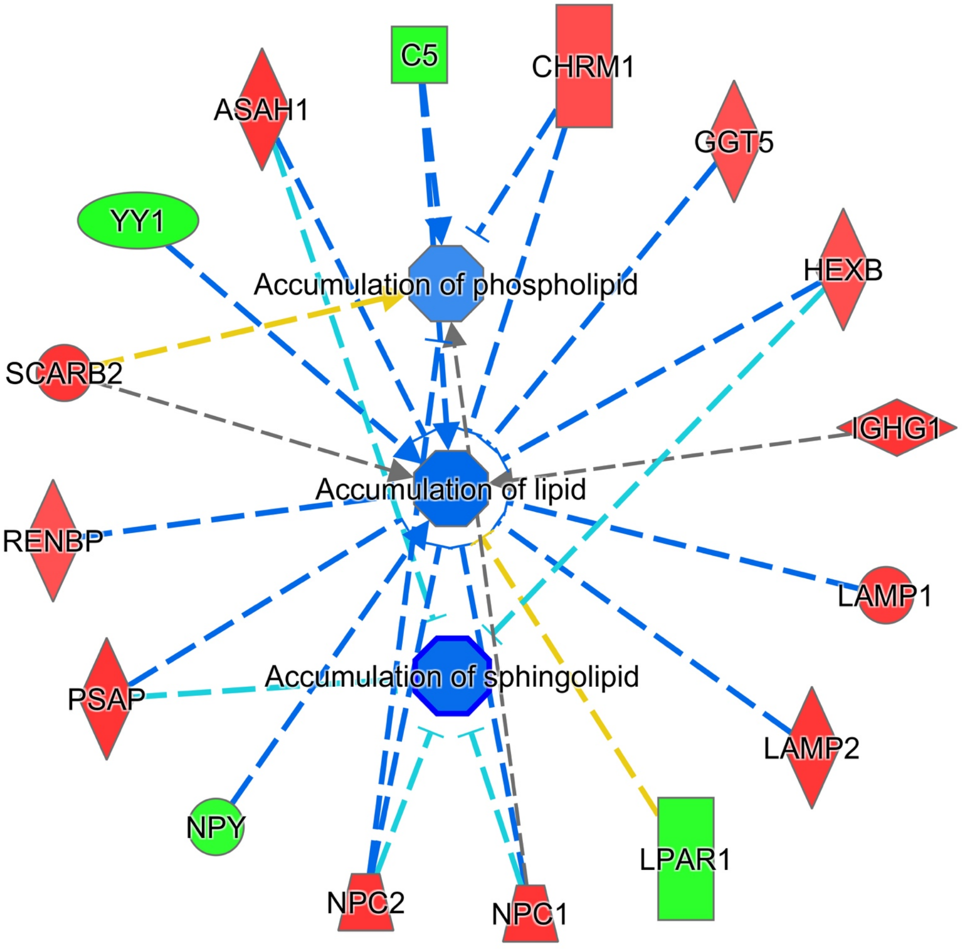

# Unedited Figures for Main Figure 4 - Disease and Cellular Function

## IMMUNE RESPONSE

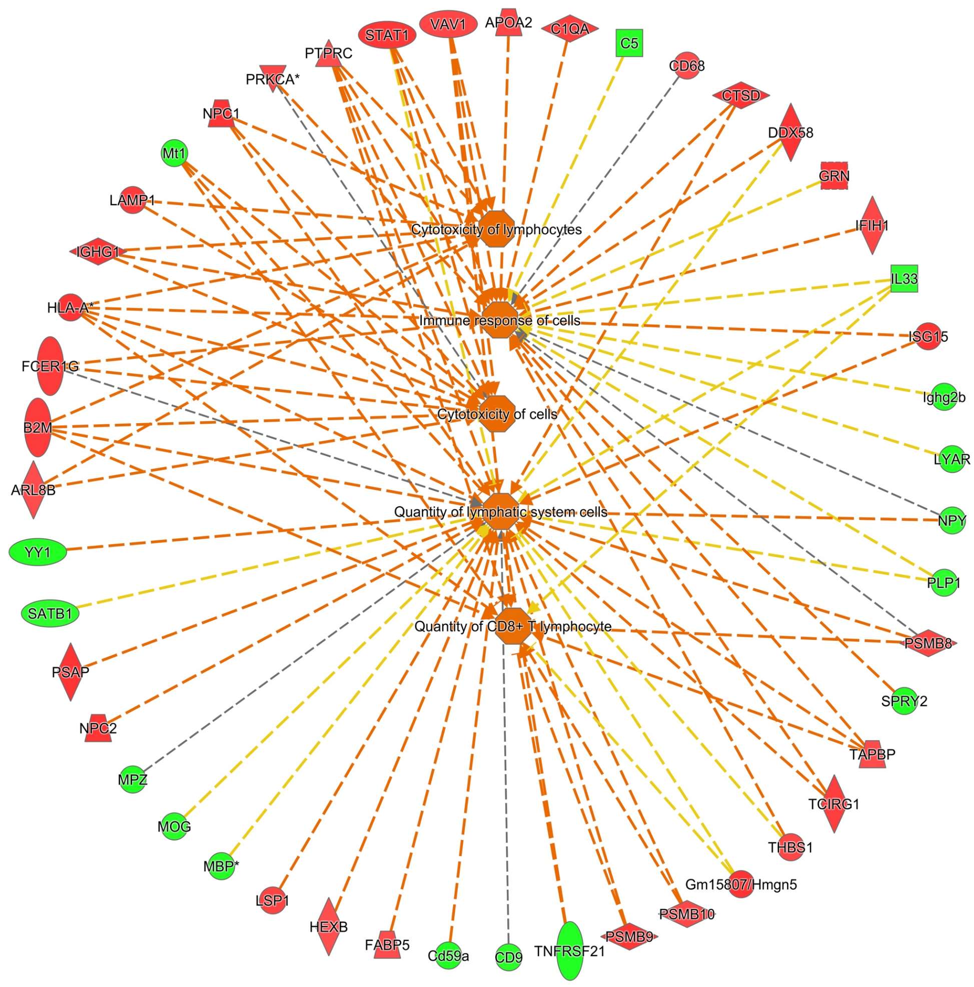

<u>**Unedited Figures for Main Figure 4 - Disease and Cellular Function**</u>

**GLIAL DEFECTS**

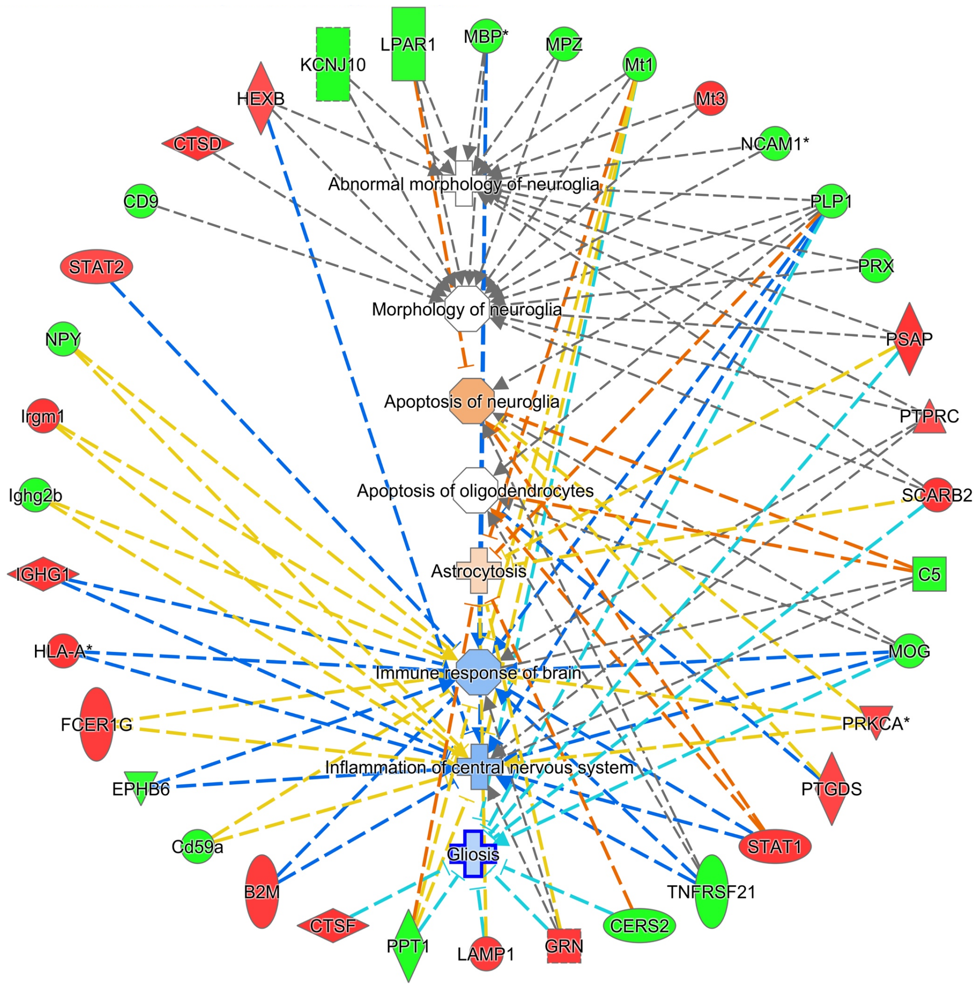

# Unedited Figures for Main Figure 4 - Disease and Cellular Function

## NEURODEGENERATION

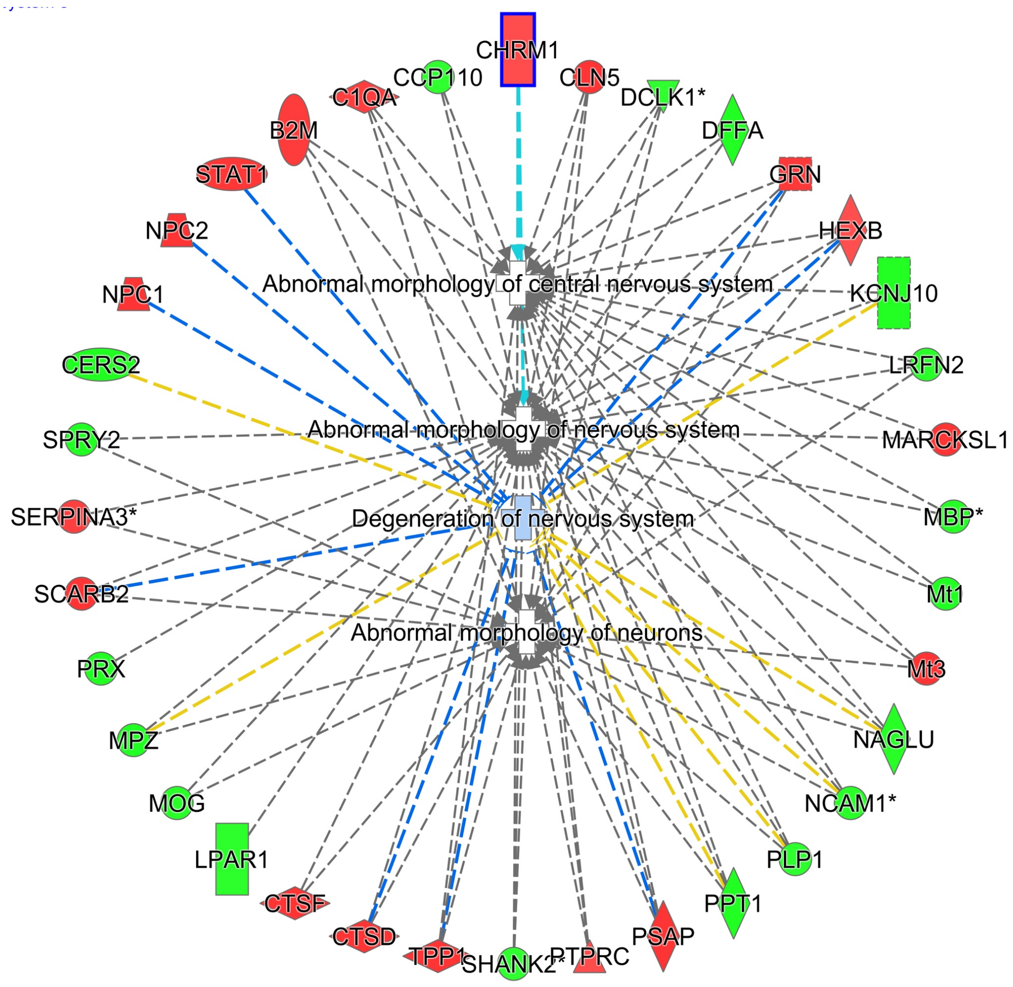

# Unedited Figures for Main Figure 4 - Disease and Cellular Function

## MOVEMENT DISORDERS

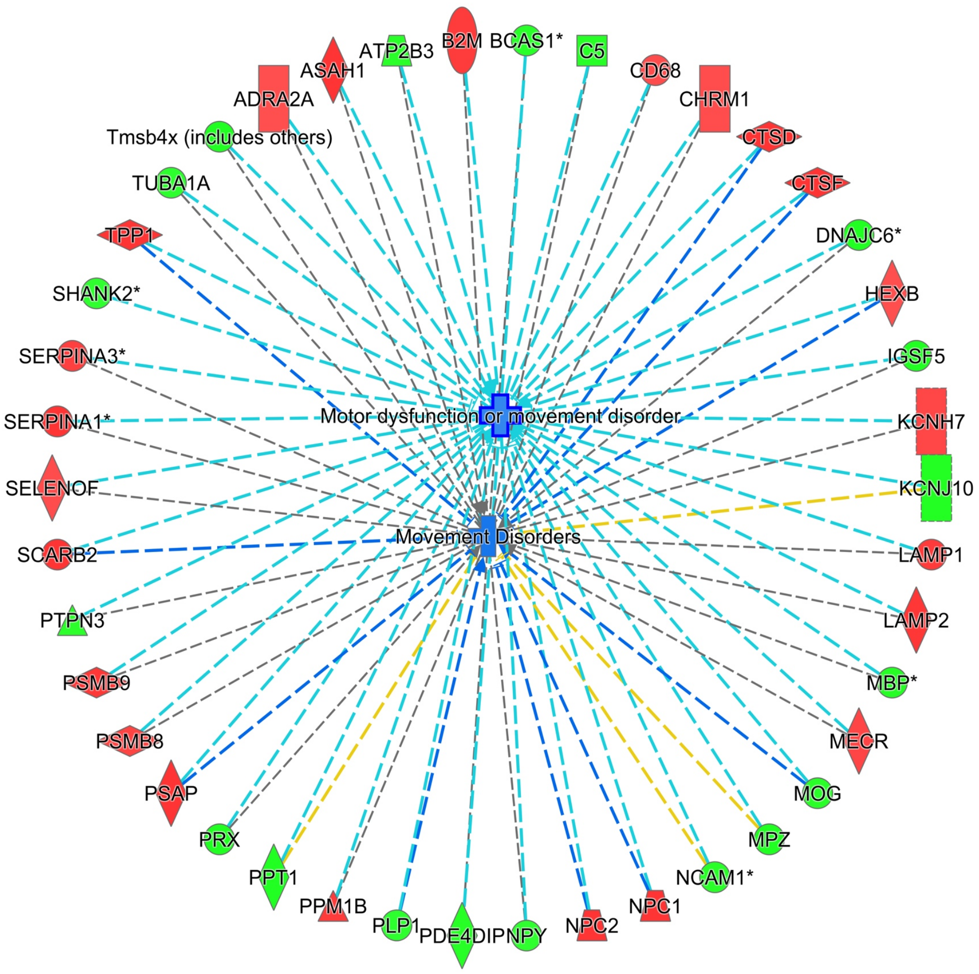

**Unedited Figures for Main Figure 4 - Disease and Cellular Function**

**ABNORMAL MYELINATION**

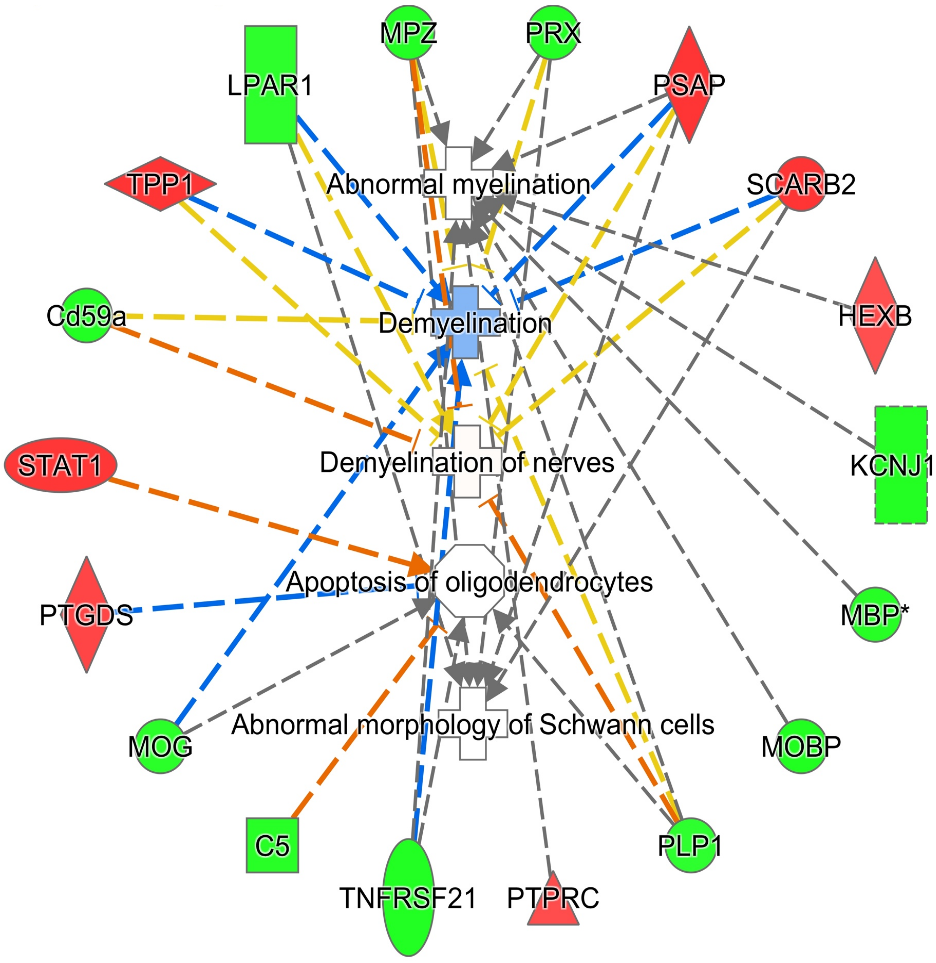

## Unedited Figures for Main Figure 4 - Disease and Cellular Function

### SENSORY SYSTEM DEVELOPMENT

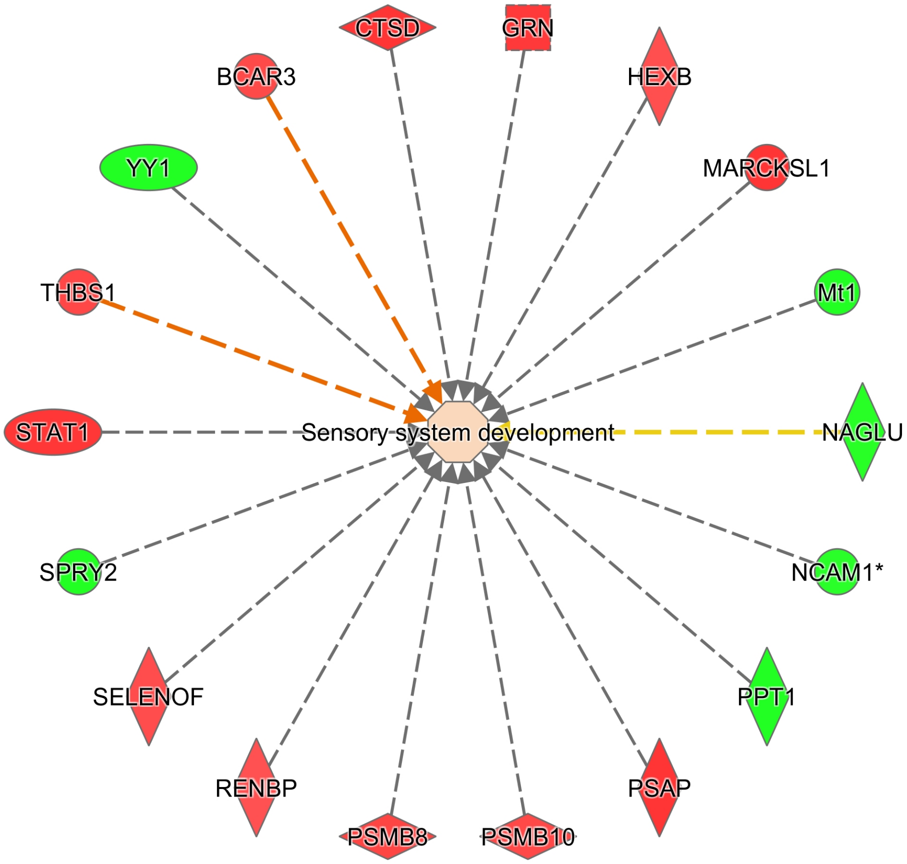

<u>**Raw western blot membrane images for cropped bands in figures**</u>

Red arrows indicate approximate molecular weight of bands analyzed and regions represented in **Supplementary Figure 2** are denoted by the red dotted box.

## SUPPLEMENTARY FIGURE 2 - SNAP25

**GEL1**

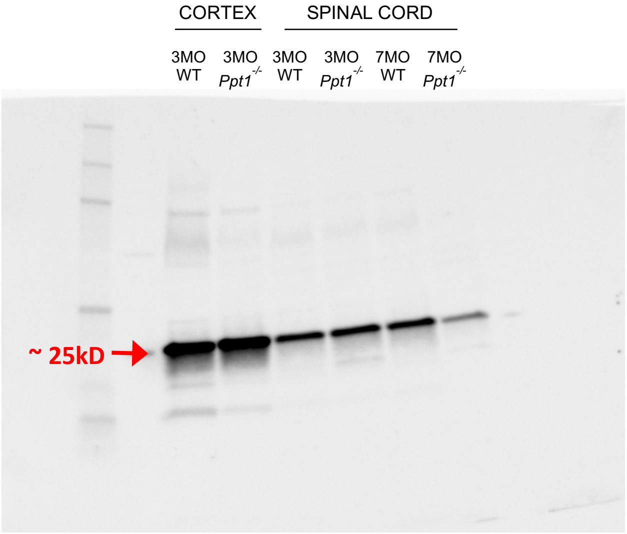


**GEL2**

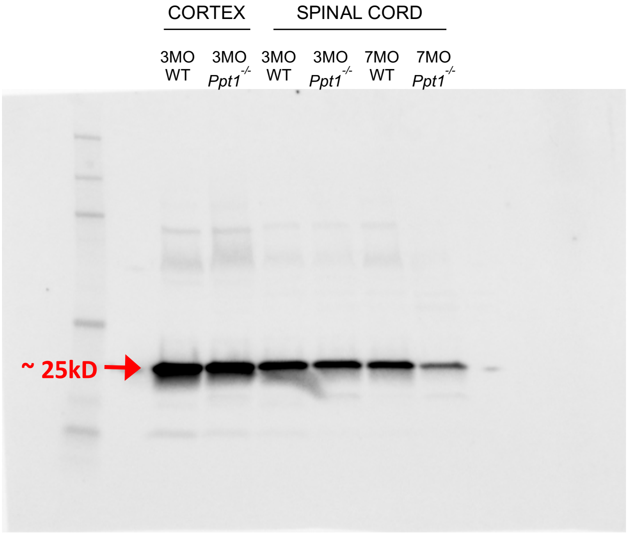


**GEL3**

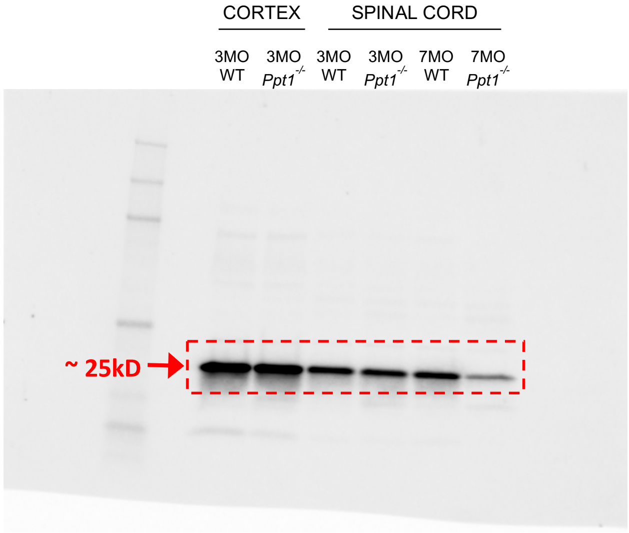

# SUPPLEMENTARY FIGURE 2 – SYNAPTOPHYSIN

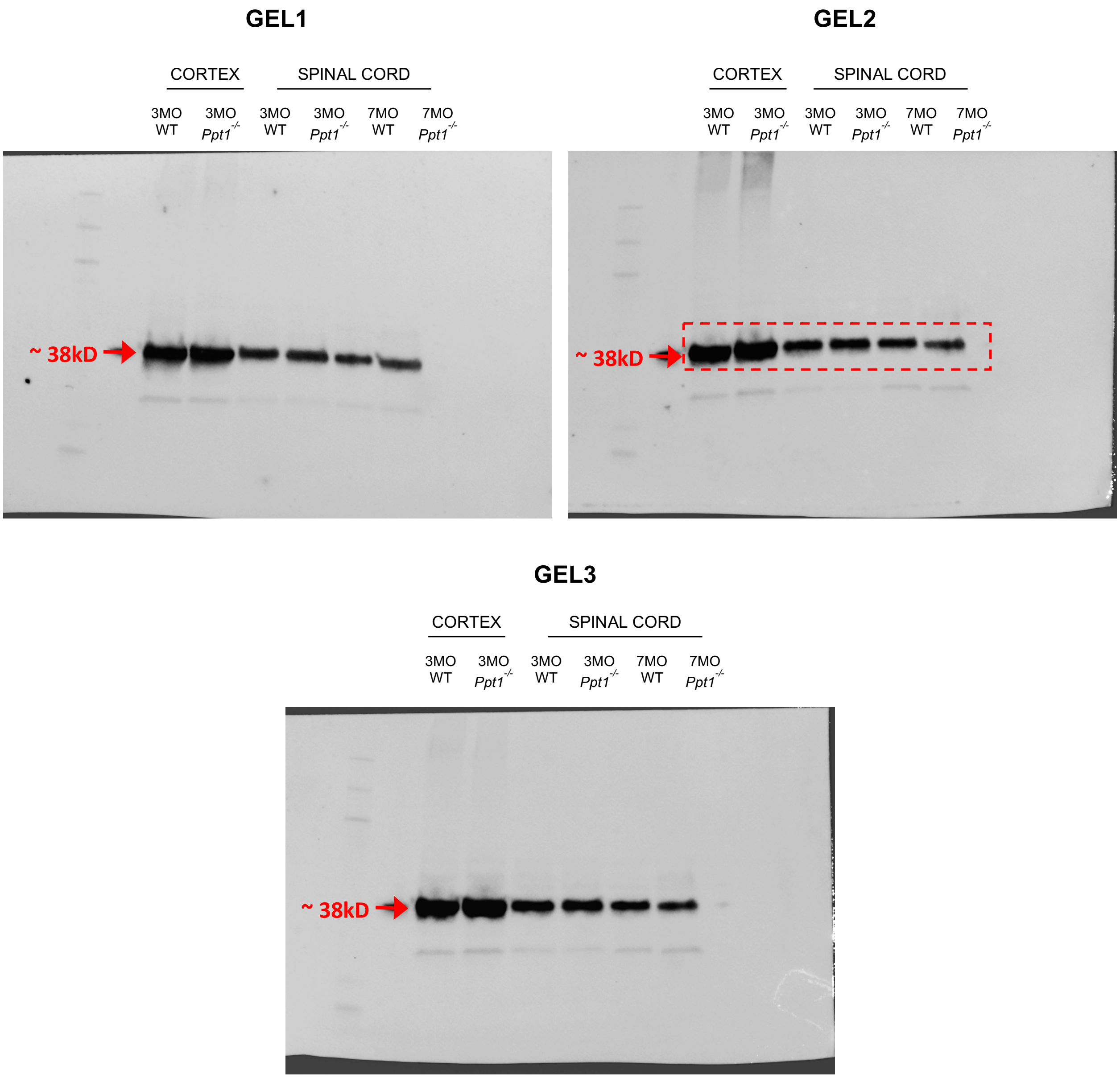

# SUPPLEMENTARY FIGURE 2 – COX IV

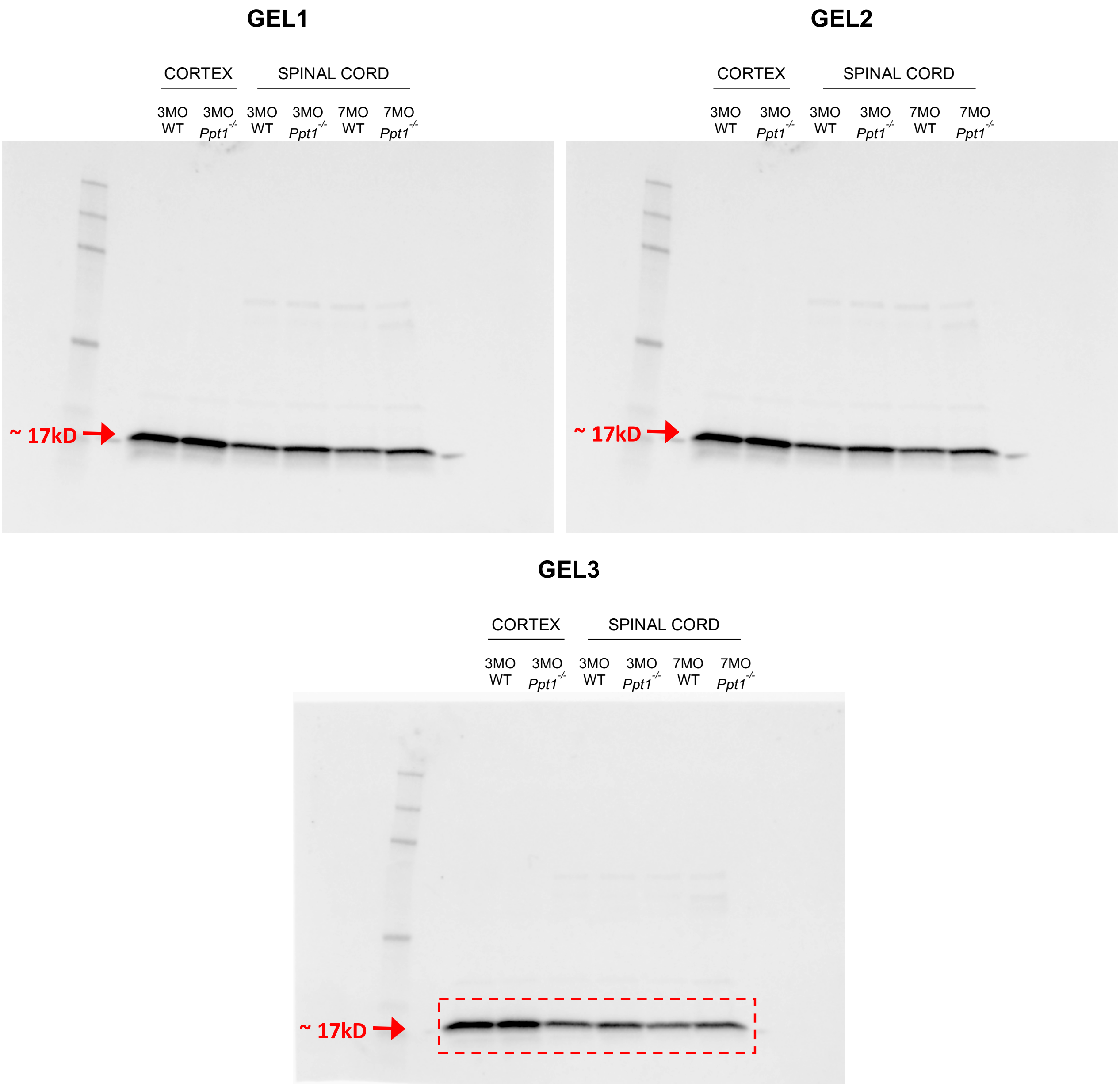

# SUPPLEMENTARY FIGURE 2 – GFAP

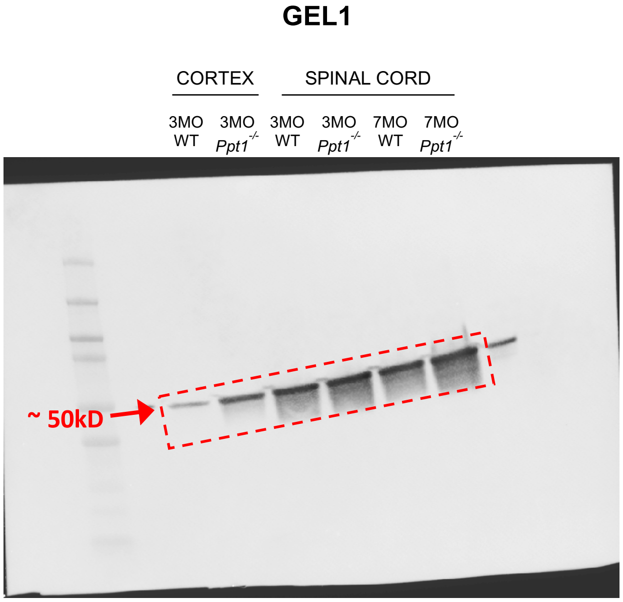


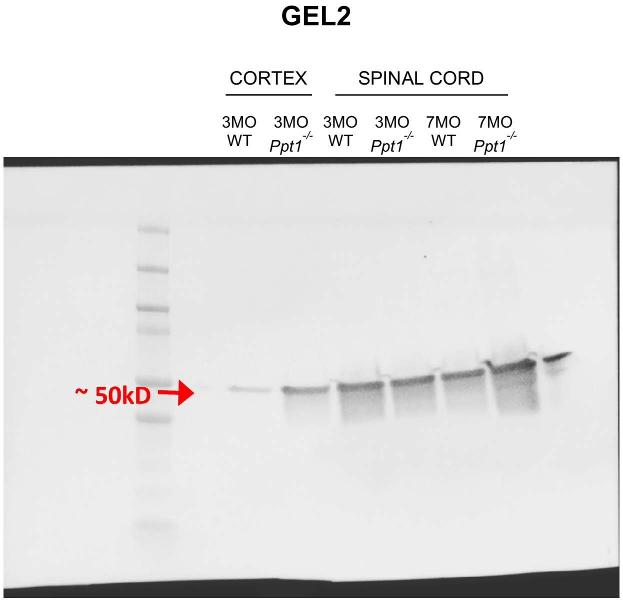


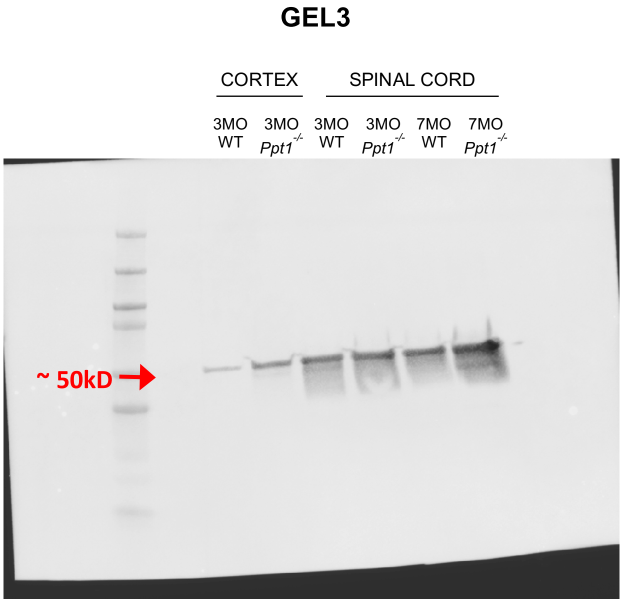

# SUPPLEMENTARY FIGURE 2 – GLUTAMINE SYNTHETASE

## GEL1

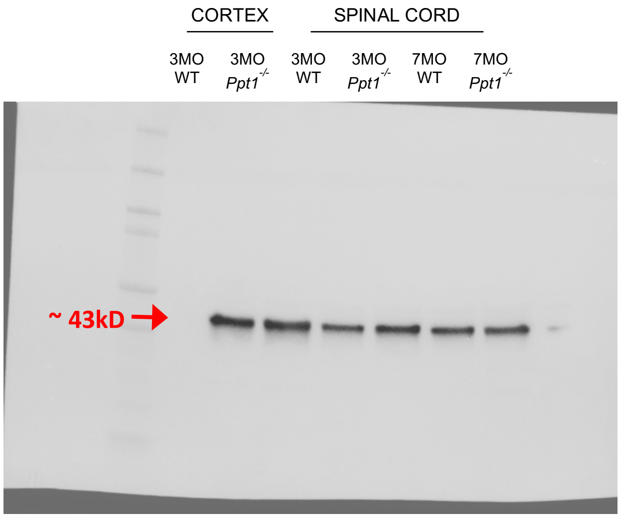


## GEL2

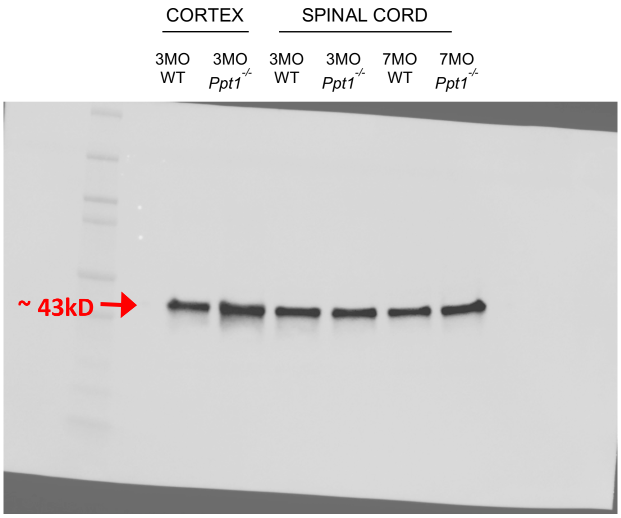


## GEL3

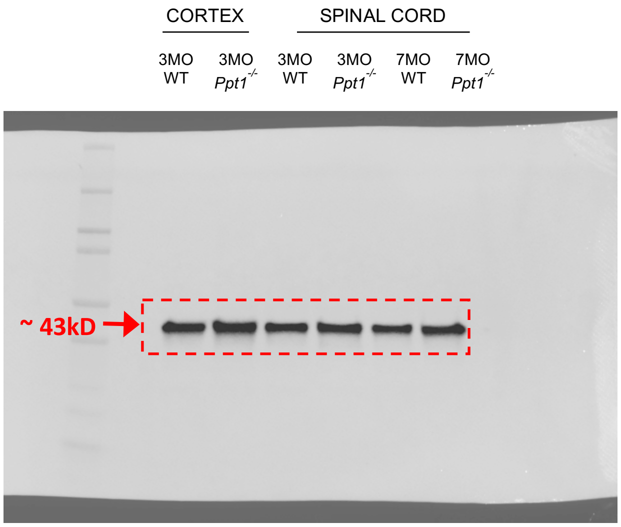

# SUPPLEMENTARY FIGURE 2 – CALBINDIN

## GEL1

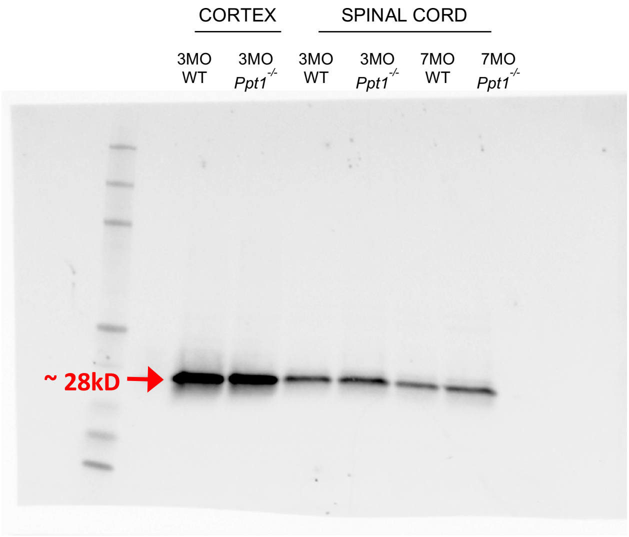


## GEL2

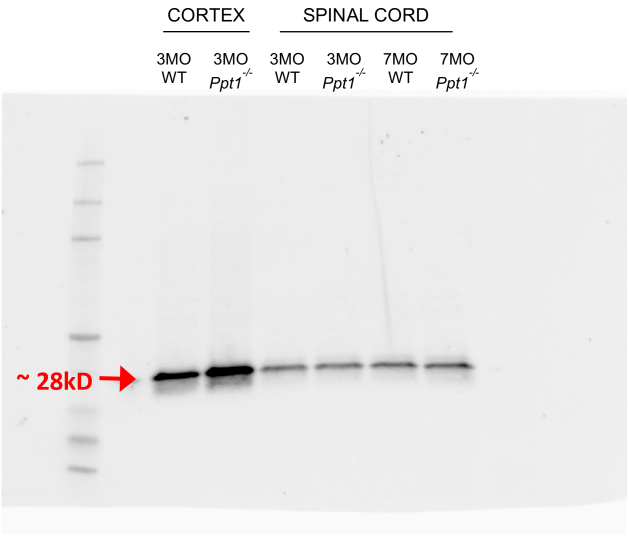


## GEL3

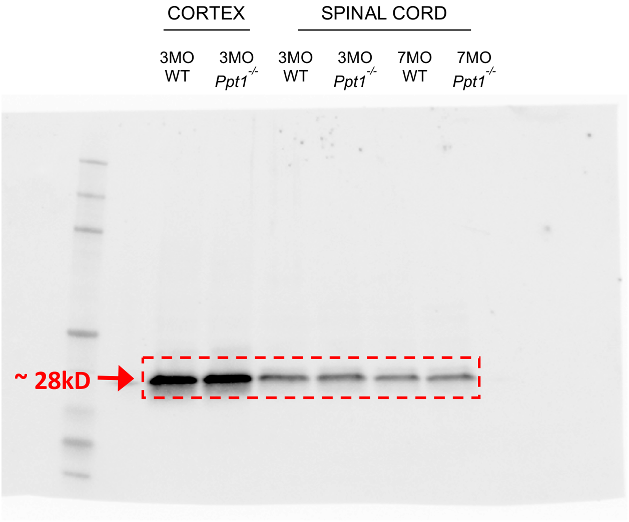

# SUPPLEMENTARY FIGURE 2 – MYELIN BASIC PROTEIN

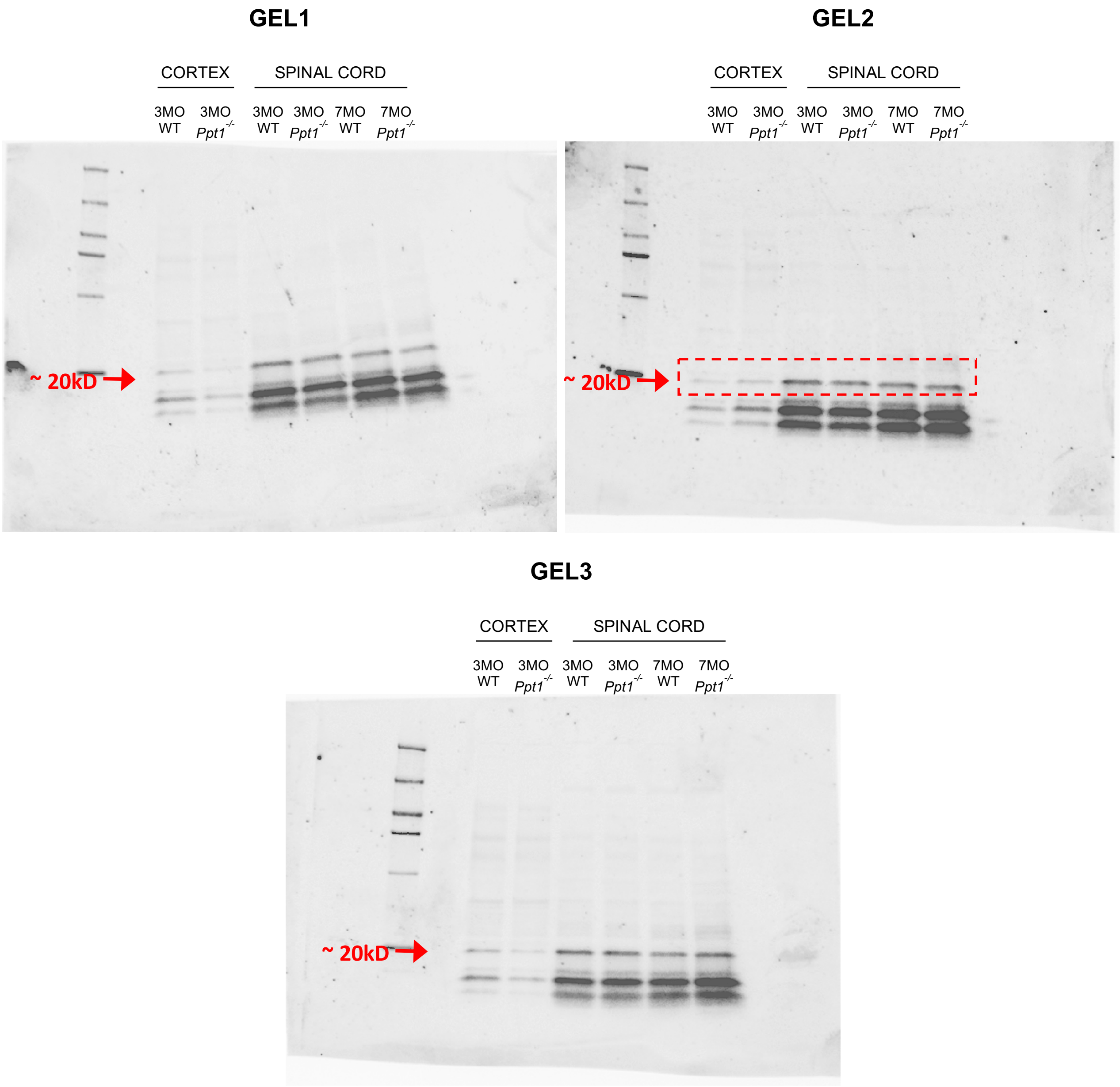

Supplement: Supplementary file 3 — Supplementary file3 [file 41598_2020_72075_MOESM3_ESM.pdf]
